# Recent advances in the design of SERS substrates and sensing systems for (bio)sensing applications: Systems from single cell to single molecule detection

**DOI:** 10.12688/f1000research.149263.3

**Published:** 2025-06-24

**Authors:** Sai Ratnakar Tadi, Ashwini G Shenoy, Anirudh Bharadwaj, Sreelakshmi C S, Chiranjay Mukhopadhyay, Kapil Sadani, Pooja Nag

**Affiliations:** 1 Department of Mechatronics, Manipal Institute of Technology, Manipal Academy of Higher Education, Manipal, Karnataka, 576104, India; 2 Department of Microbiology, Kasturba Medical College, Manipal Academy of Higher Education, Manipal, Karnataka, 576104, India; 3 Department of Instrumentation and Control Engineering, Manipal Institute of Technology, Manipal Academy of Higher Education, Manipal, Karnataka, 576104, India

**Keywords:** Raman effect, Surface Enhanced Raman Spectroscopy, Disposable substrates, Point-of-use, Single-molecule sensing, Bioreceptor.

## Abstract

The Raman effect originates from spontaneous inelastic scattering of photons by matter. These photons provide a characteristic fingerprint of this matter, and are extensively utilized for chemical and biological sensing. However, the phenomenon do not hold potential for its direct use in sensing applications, since the generation of the Raman scattered photons is inherently low. Surface enhanced Raman spectroscopy (SERS) overcomes the low sensitivity associated with Raman spectroscopy and assists the sensing of diverse analytes, including ions, small molecules, inorganics, organics, radionucleotides, and cells. Plasmonic nanoparticles exhibit localized surface plasmon resonance (LSPR) and when they are closely spaced, they create hotspots where the electromagnetic field is significantly enhanced. This amplifies the Raman signal and may offer up to a 10 ^14^-fold SERS signal enhancement. The development of SERS active substrates requires further consideration and optimization of several critical features such as surface periodicity, hotspot density, mitigation of sample or surface autofluorescence, tuning of surface hydrophilicities, use of specific (bio) recognition elements with suitable linkers and bioconjugation chemistries, and use of appropriate optics to obtain relevant sensing outcomes in terms of sensitivity, cross-sensitivity, limit of detection, signal-to-noise ratio (SNR), stability, shelf-life, and disposability. This article comprehensively reviews the recent advancements on the use of disposable materials such as commercial grades of paper, textiles, glasses, polymers, and some specific substrates such as blue-ray digital versatile discs (DVDs) for use as SERS-active substrates for point-of-use (POU) sensing applications. The advancements in these technologies have been reviewed and critiqued for analyte detection in resource-limited settings, highlighting the prospects of applications ranging from single-molecule to single-cell detection. We conclude by highlighting the prospects and possible avenues for developing viable field deployable sensors holding immense potential in environmental monitoring, food safety and biomedical diagnostics.

## 1. Introduction to Raman effect for sensing

Raman spectroscopy is an analytical technique used to sense a diverse range of analytes by providing insights into their molecular structure and bonding. When illuminated with a suitable light source, the intra-and intermolecular vibrations of the sample result in a wavenumber shift of the scattered photons, generating a unique Raman fingerprint spectrum. In contrast to near-infrared (NIR) spectroscopy, Raman spectroscopy generates a unique fingerprint that is insensitive to the bulk properties of the matrix and masking effects of water in the aqueous phase. Raman spectroscopy relies on Raman scattering, experimentally discovered as a modified scattered radiation by Raman and Krishnan in 1928, initially annotated as “a new type of secondary radiation’ ( Raman & Krishnan, 1928). Further studies indicated that Raman scattering of the incident light resulted in scattered radiation of a lower frequency than that of the incident light. Therefore, Raman scattering is mathematically calculated as a measure of the phase shift between incident and Raman-scattered photons.

Raman spectroscopy is extensively used in research for industrial quality control applications, assessment of environmental safety, healthcare as diagnostics, and nutritional security owing to its characteristic fingerprinting ability for diverse analytes. Its use in healthcare is predominantly directed towards metabolite sensing ( F. Hu et al., 2014; L. Wei & Vikesland, 2015), diagnosis of infectious diseases and carcinoma ( Tipping et al., 2017), mapping of drug distribution ( Tipping et al., 2016), and whole cell detection ( Crawford et al., 2012) in other allied areas of bioscience ( Benevides et al., 2005). Other prominent domains of application include pharmaceuticals ( Choo-Smith et al., 2002), nanostructure characterization ( Bensebaa et al., 1999), forensic studies, analytical chemistry ( Doty et al., 2015), phase transitions ( Elleuch et al., 2006), solid-state physics ( Jawhari, 2000), and archaeology ( Kiefer, 2007).

Most asymmetric molecules display a weak Raman effect, thus requiring the use of strong illumination, such as lasers, to obtain measurable Raman-scattered photons. The use of appropriate optics such as lenses, notch filters, and monochromators as spectral filters, differently cooled detector arrays comprising charge-coupled devices (CCDs), complementary metal-oxide-semiconductor (CMOS) or avalanche photodiodes (APDs), and silicon photomultipliers (SiPMs) as detectors improves the sensitivity by several orders of magnitude ( Mukhopadhyay, 2007). However, as the Raman effect is a weak phenomenon, its direct use in trace-level analysis of analytes is limited, and technologies for its amplification have been extensively studied.

### 1.1 Mechanism of Raman scattering

**Figure 1.**
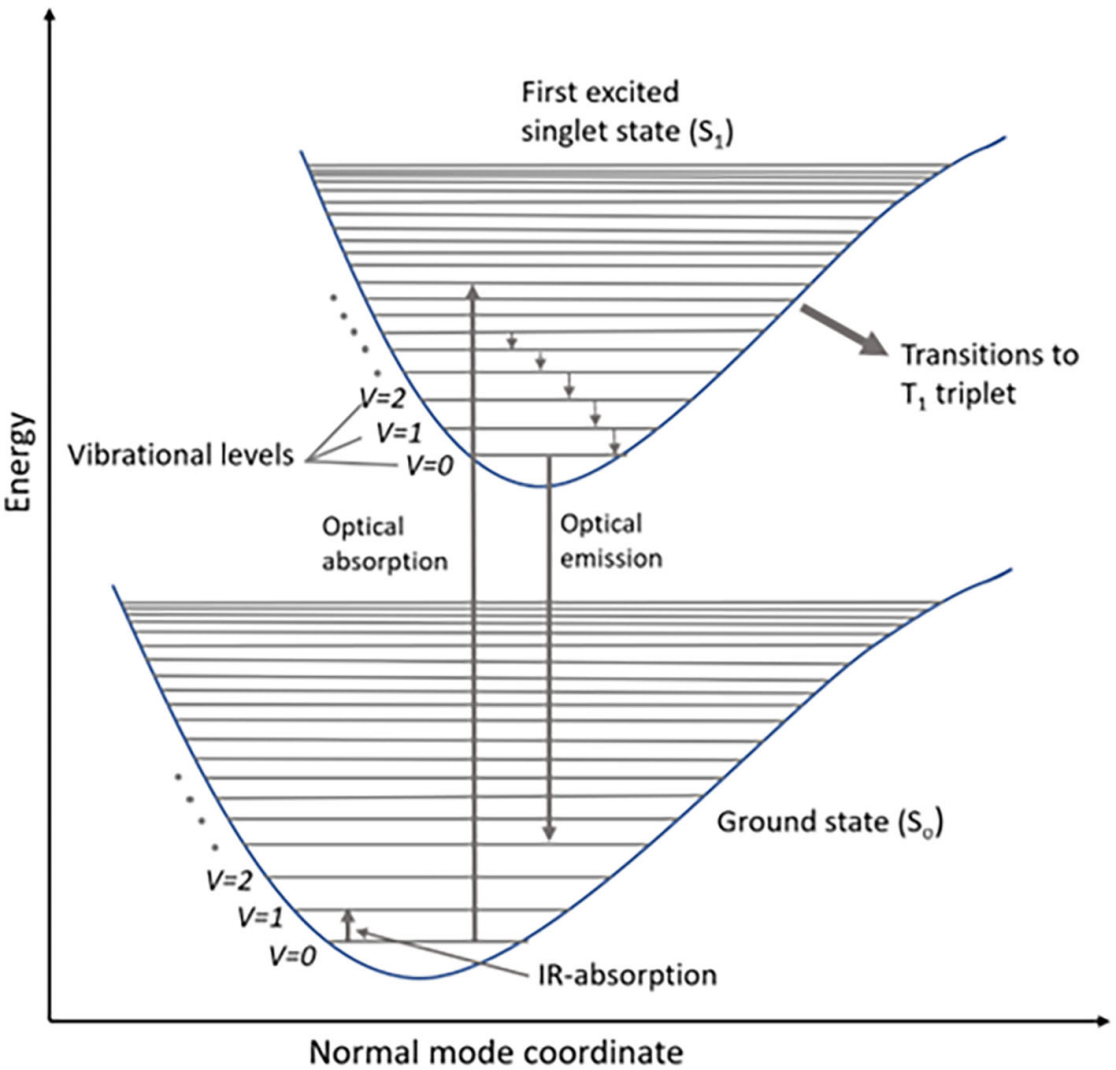
Schematic representation of energy levels of a molecule in a Jablonski diagram.

Raman scattering involves the inelastic transfer of momentum from photons of the incident light to molecules in the sample. The interaction of the external electromagnetic field with the electron subsystems of the molecule results in light scattering. These molecular energy transitions during scattering are shown in the Jablonski diagram, which illustrates the electronic states: ground state, singlet state (S _o_), and triplet state (T), as shown in Figure 1. A typical Jablonski diagram contains vertically stacked electronic states and horizontally grouped vibrational energy states, according to their spin multiplicity. Each energy state contains sublevels based on the vibrational energy of the molecule ( Demtröder, 2015). According to Pauli’s exclusion principle, all electrons are paired in the ground state of the molecule, while a change in the spin of the energy state is observed in the singlet state (S _0_) due to half-filled molecular orbitals. The electronic transitions to higher singlet energy states are caused by photon energy absorption from the incident electromagnetic radiation, whereas the emission is a two-photon process, with single-photon emission to the lowest vibrational state (S _1_). A triplet state (T _1_) is a virtual triple-degenerate excited state for the relaxation of excited photons to the ground state by intersystem crossing ( Prochazka, 2016). The standard expression for the energy of the photon transition is illustrated in ( Equation 1), where h = Planck’s constant = ~6.626 x10 ^−34^ J/s and c = speed of light = ~3 × 10 ^8^ m/s.

E=hυ=hc/λ=hω=hcṽ (1)

**Figure 2.**
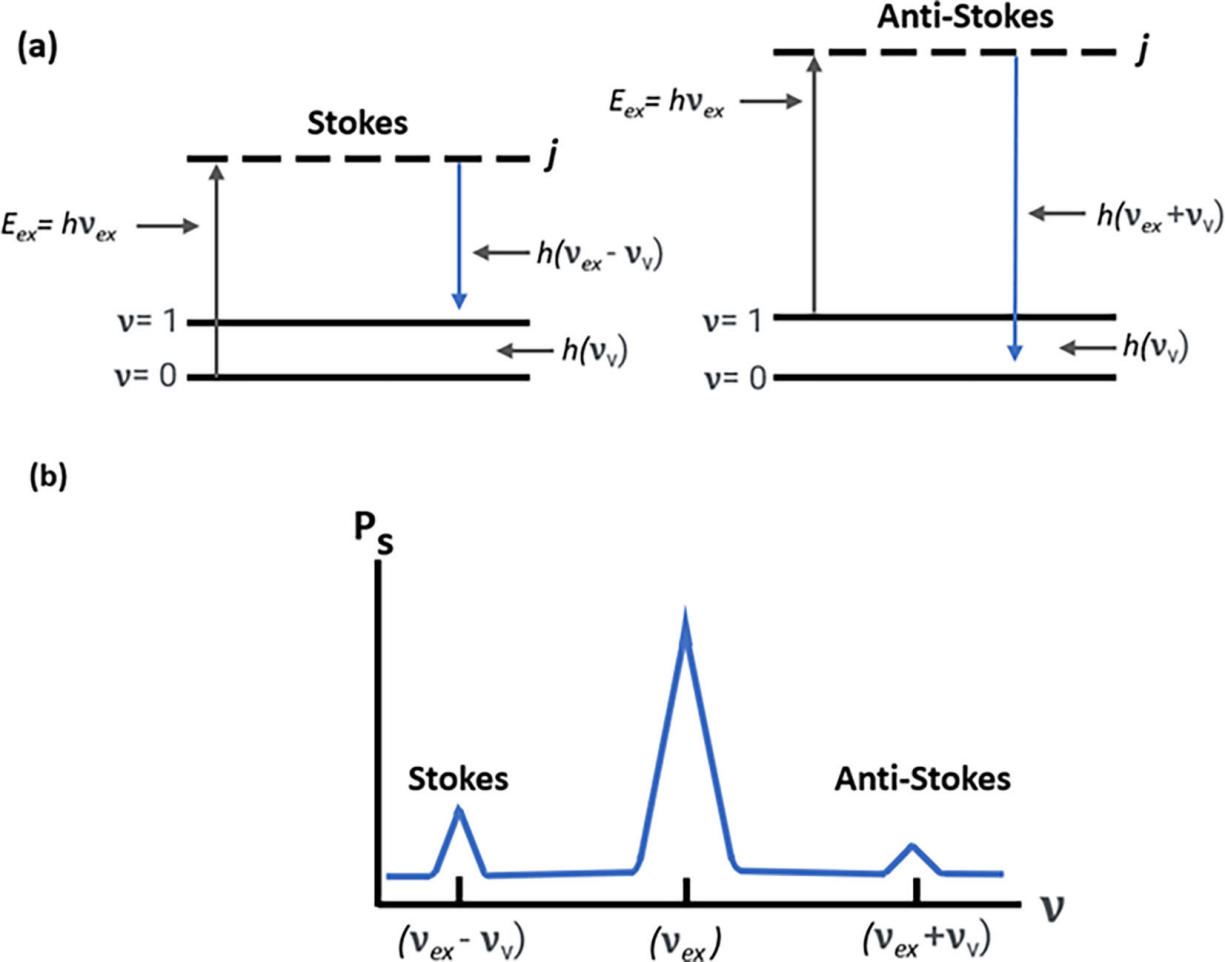
(a) Stokes and anti-stokes scattering due to energy changes in electronic excitation and emission. (b) Raman spectrum depicting Stokes scattering and anti-stokes scattering.

However, in addition to optical absorption and emission, another process termed as scattering is often observed in the emission of a molecular photon owing to the absorption of an incident light photon. This photon scattering may be elastic or inelastic. Rayleigh scattering, an elastic scattering, is associated with energy conservation during photon emission, whereas the inelastic scattering associated with the transfer of energy between photons and molecular subsystems is termed Raman scattering. Furthermore, any Raman active molecule in the lowest vibrational state that absorbs the photon energy causes a decreased frequency and higher wavelength (λ _S_ > λ _L_) of the scattered photon, resulting in a lower energy of the scattered photon (E _S_ < E _L_), termed as the Stokes process, as shown in Figure 2(a). In contrast, the anti-Stokes process occurs because of the emission of excessive energy from the molecule in the excited vibrational state with a lower wavelength (λ _S_ < λ _L_) and a higher energy (E _S_ > E _L_) of the scattered photons ( Eric Le Ru, 2009). The Raman shift is only dependent on the material subjected to the Raman effect; a negative shift is associated with the anti-Stokes process, and a positive shift with the Stokes process. Because of the lower energy associated, the anti-Stokes Raman scattering process is weaker than the Stokes process ( Blackie et al., 2009).

$$ṽ=1/\lambda =1/c\;\left(\omega_{\text{incident}}-\omega_{\text{scattered}}\right)$$ (2)

Nonetheless, the abundance of photons involved in Stokes and anti-Stokes processes is significantly lower. A comprehensive study by D.A. Long stated that one out of 10 ^7^ photons incident on a sample may be scattered, resulting in the Stokes or anti-Stokes process. The Raman effect is also significantly affected by the components of the optical system, as it can limit the sensitivity with some major design considerations, such as the wavelength and power of the laser ( Glass, 1967), spectral resolution ( Meier et al., 1988), collection optics ( Greenler & Slager, 1973), range and sensitivity of the detector ( Allemand, 1970), and minimizing Rayleigh scattering ( Cutler et al., 1980). Typically, near-infrared (NIR) laser-equipped Raman systems are employed to test organic ( Košek et al., 2020) and biological specimens ( Synytsya et al., 2014) in order to minimize fluorescence and facilitate easier penetration into the sample matrix. Efficient Raman scattering can be achieved only with optimum laser power. A lower power generates weak Raman signals, while high power can generate fluorescence, which in most cases may lead to sample degradation. In addition, spectrometers with a lower spectral resolution cannot distinguish between closely-spaced Raman peaks. The light collection efficiency of a spectrometer is significantly affected by collection optics; hence, high-numerical-aperture (NA) lenses are commonly used in commercial Raman systems. Rayleigh scattered light is a significant impediment to producing better Raman signals, which can be mitigated with notch filters. Other considerations include the choice of optical components for minimal autofluorescence and the stable alignment of the optical components for the highest light throughput.

Notwithstanding these challenges, definitive fingerprinting ability can assist in the development of Raman-based optical sensing systems and their use in sensing diverse inorganic molecules ( Mycroft et al., 1990), biomolecules ( Larsson & Rand, 1973), and bacterial cells ( Layne & Bigio, 1986). Most of the Raman spectrometers before the 1990s used low-energy argon ion lasers with a high laser power of 200–500 mW and silicon photodiodes as detectors, thereby producing fluorescence and a low signal-to-noise ratio (SNR). The use of plasmonic nanoparticle-modified substrates may amplify Raman scattering at low laser power, which enhances the Raman effect. The Raman shift observed in the presence of resonating electronic clouds around noble metal surfaces enhances molecular scattering, and this surface enhancement is called Surface-Enhanced Raman Scattering (SERS) ( D.A. Long, 1977).

## 2. Surface Enhanced Raman Spectroscopy (SERS)

SERS involves the amplification of the Raman scattered signal of target analyte molecules in the presence of a resonating plasmonic field. The Raman effect states that the photon-molecule interactions may result in photon scattering, which generates a dipole moment (μ _ind_) that is directly proportional to the polarizability (α _m_) of the molecule, as shown in ( Equation 3).

$$\mu_{\mathrm{ind}}=E_{\text{incident}}.\alpha_{m}$$ (3)

where, E _incident_ is the incident electric field generated by the external electric field on the system.

As discussed in Section 1, Raman-based sensing systems require high laser power and longer exposure durations for analyte sensing at lower concentrations. In contrast, SERS-based sensing systems detect target analytes in complex samples at low laser powers and with minimal exposure durations. Typically, SERS systems use metal-based plasmonic nanoparticles to synthesize SERS-active substrates. The Raman scattering of the plasmonic nanoparticles was amplified using a laser with a similar range of excitation wavelengths. During the mid-1970s, Jeanmarie *et. al.,* identified the SERS effect while studying the Raman effect of pyridine on a roughened silver electrode surface ( Jeanmaire & Van Duyne, 1977), further widening its application in various fields of study up to single-molecule detection. Commonly seen SERS-active substrates are noble metal nanostructures, such as colloidal gold and silver nanostructures with sizes range from to 10-150 nm. These noble metal-based substrates were observed to improve the Limit of Detection (LoD) by amplifying the Raman signals; however, the mechanism of enhancement is not clearly understood ( Fleischmann et al., 1974). Later studies indicated that silver (Ag), gold (Au), and copper (Cu) nanostructures can enhance signals by generating larger localized surface plasmons (LSPs) in the field of lasers. The SERS effect was identified as a dual effect, that is, chemical enhancement (CE) and electromagnetic enhancement (EM) of the molecule-metal nanostructure interaction. The physical basis of EM is clear and was successfully altered to obtain an enhancement of six to eight orders of magnitude, while the basis for CE is still not clear.

SERS systems offer some potential advantages over conventional Raman systems, such as (i) higher signal enhancement owing to higher Raman scattering ( Pérez-Jiménez et al., 2020), (ii) better signal-to-noise (SNR) ratio that detects weak Raman signals despite any background fluorescence ( Pérez-Jiménez et al., 2020), (iii) distinction of spectra for multiplex detection of analytes, ( Vo-Dinh, 1998) (iv) rapid real-time monitoring owing to greater Raman scattering ( Etchegoin et al., 2003), and (v) imaging and chemical mapping by integration with microscopy techniques ( McGuire et al., 2001). However, there are some important design considerations for the development of SERS systems for commercial purposes. For example: (i) selection of an appropriate wavelength laser; shorter wavelength lasers require higher power, in contrast to common NIR lasers that possess better SNR, (ii) use of additional optical components, such as a high NA lens and mirrors for better collection of scattered light, (iii) anti-reflective coatings to avoid undesirable reflections and better SNR, (iv) use of edge filters to avoid transmittance of Rayleigh scattering to the detector, (v) use of a low-noise, high-sensitivity photodetector with minimal thermal noise during prolonged use of the laser, and (vi) alignment of the optical components to reduce the vibrations. The optimization of a SERS system with all aforementioned considerations are essential to develop commercial or point-of-use SERS sensing systems.

### 2.1 Chemical enhancement

Chemical enhancement (CE) is defined as the amplified Raman scatter signal of a target analyte resulting from the interaction between the adsorbed analyte molecule and the plasmonic nanostructure. It is calculated as the sum of differences in Raman polarizability due to the molecule adsorption onto the metal surface and the charge transfer mechanism, given by Equation (4). However, the basis of CE has been the subject of debate for decades, particularly the science that governs the SERS chemical enhancement factor (EF) ( Eric C. Le Ru & Pablo G. Etchegoin, 2009). Molecular adsorption can be physisorption or chemisorption with a bond energy of ~40 kJ/mol ( Aroca, 2006). Charge transfer in the metal-molecule complex is due to the incident light energy corresponding to the electronic transitions of the molecule with the underlying phenomenon of Resonance Raman Scattering.

$$I_{\text{Raman}}\propto 1/\lambda^{4}\;{\left|\partial \alpha /\partial Q\right|}^{2}$$ (4)

where I _Raman_ is the intensity of the Raman scattering, λ is the wavelength of the incident laser used, α is the polarizability of the molecule, and Q is the normal mode coordinate.

One of the widely accepted theories for chemical enhancement is the Charge Transfer (CT) mechanism proposed by the SERS pioneer Andreas Otto, where an adsorbed molecule subsequently changes the molecular polarizability, thereby enhancing the Raman scattering ( Otto et al., 1992). For example, an incident photon with a frequency ν _inc_, in resonance with the surface-adsorbate complex, causes excitation and return of the metal electron to its ground state because of the CT mechanism. However, if the excited electron resides in the lowest unoccupied molecular orbital (LUMO) for a time period shorter than the absorbed photons, it will be scattered with dissimilar energy levels than the incident photons (ω _s_ = ω _inc_ − ω _vib_).

Chemical enhancement can be due to intramolecular resonance ( Creighton, 1983), ground-state charge transfer, ( Lippitsch, 1984) or resonant charge transfer ( Lombardi et al., 1986). Intramolecular resonance assists in improving Raman scattering due to coherence of analyte’s molecular vibrations with the frequency of the selected excitation wavelength. However, intramolecular resonance is affected by the area of the Raman cross-section, as larger cross-sections assist in the greater scattering of some weak scattering molecules ( Tauber & Mathies, 2002). Other key parameters include the chemical structure of the analyte ( Ryder, 2005) and the shape, size, and dielectric properties of the plasmonic nanostructure ( Jensen et al., 2000) ( Notingher & Elfick, 2005). Another cause of chemical enhancement is ground-state charge transfer, a process of electron transfer between the metal surface and the adsorbed molecules to generate charged species, which typically assist the formation of coordination compounds ( Flamigni et al., 2007) and redox reactions ( Fukuzumi, 1997). However, the ground state charge transfer is associated with specific limitations, such as the selective enhancement of molecular vibrations of the analyte molecule, ( Rurack et al., 2000) thereby limiting the uniform enhancement. Additionally, the charge transfer dynamics are affected by environmental factors ( Fleming et al., 1988), the spectral overlap with other vibrational modes of the analyte ( Blandamer & Fox, 1970) can limit the selectivity, and ground state saturation with the electrons of the analyte can limit any further enhancement. In the other chemical enhancement mode, resonant charge transfer was observed to overcome the limitations of ground-state charge transfer. This is the electron transfer from the resonating energy levels between the plasmonic metal and analyte molecule by charge transfer between the highest occupied molecular orbital (HOMO) and lowest unoccupied molecular orbital (LUMO), which alters the local electromagnetic field of the analyte molecule, resulting in greater Raman scattering. The improvement in resonant excitation enhances the plasmonic metal nanostructure and analyte interaction ( McNay et al., 2011) and facilitates enhanced Localized Surface Plasmon Resonance (LSPR) of the plasmonic substrates ( Kleinman et al., 2013), thereby maximizing the enhancement effect. Despite these modes of signal improvement associated with chemical enhancement, their significance to the overall average enhancement factor was marginal. Hence, the use of noble metal substrates is also being explored to achieve better signal enhancement by altering the surface chemistry of substrates and modifiers.

However, the contribution of the CE to the average SERS enhancement was not significant. Therefore, the choice of materials used for SERS substrate synthesis requires meticulous consideration of the nanostructure morphology, light absorbance range, stability, ( Yamada et al., 1987) and hydrophobicity, which may be tailored to improve plasmonic resonance and electromagnetic enhancement.

### 2.2 Electromagnetic enhancement

**Figure 3.**
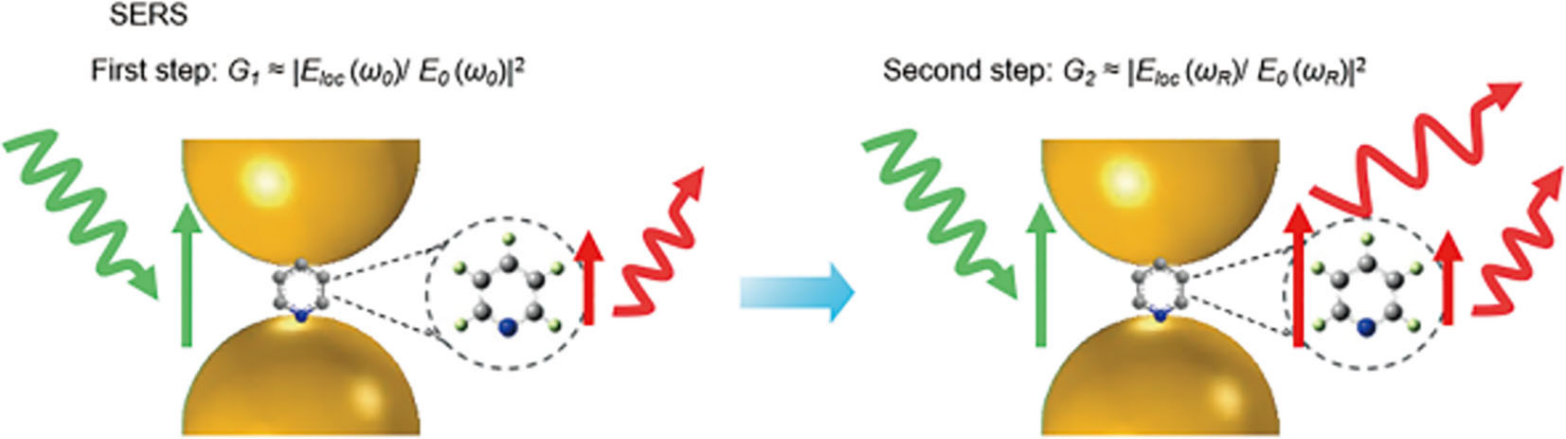
Schematic illustration of electromagnetic (EM) enhancement with specifics of the two-step enhancement mechanism (This figure has been reproduced with permission from ( Ding et al., 2016) Copyright (2016) Nature Reviews Materials).

Electromagnetic (EM) enhancement refers to the amplification of Raman-scattered photons in the proximity of a resonating electron cloud at the metal-dielectric interface. Surface plasmon resonance (SPR) is generated by the cumulative resonance on the metal surface by the coherence of the electron cloud oscillation frequency at the metal-dielectric interface with the frequency of the excitation laser. In contrast, resonance localized at a position with plasmonic nanoparticles is termed LSPR. The LSPR effect can absorb or scatter the incident laser and potentially enhance the local electromagnetic field. Local field enhancement requires the molecule to be in proximity (within ~ 100 nm) to the metal surface, that is physisorption or chemisorption. The field enhancement factor is also prominently dependent on the laser power and Stokes and anti-Stokes effects ( Ding et al., 2016). Similarly, EM enhancement is a coupling effect of the local field and the re-radiated (Raman) field of the SERS substrate, as shown in Figure 3. As previously discussed, the localized electromagnetic field for metals is higher if the excitation wavelength (λ _L_) is close to the electromagnetic resonance of the system. Hence, the localized electric field (E _loc_) is dependent on light polarization and its excitation wavelength. SERS hotspots are generated if the magnitude of │E _loc_│ is greater than │E _inc_│. The local field intensity enhancement factor (M _loc_ (w _L_)) would be increased by a factor of:

$$M_{\mathrm{loc}}\left(\omega_{L}\right)=\frac{{\left|E_{\mathrm{loc}}\left(\omega_{L}\right)\right|}^{2}}{{\left|E_{\mathrm{loc}}\right|}^{2}}$$ (5)

where, (ω _L_) is the frequency of the local electric field, M _Loc_ (ω _L_) is the local field enhancement, │E _loc_ (ω _L_)│ ^2^ is the magnitude of the local electric field amplitude, and │E _loc_│ ^2^ is the magnitude of the incident electric field amplitude.

In addition to local field enhancement, re-radiated field enhancement is also prominent for EM enhancement. In the electromagnetic model, the molecule is considered a dipole that responds to a greater local field near the surface ( Kerker, 1984). The interaction of a metal nanostructure with light generates an LSPR effect on the nanostructure, which amplifies the incident EM field and scattered Raman field. Under SERS conditions, the radiation of the Raman dipole of a molecule in proximity to the metal surface modifies the exciting field, termed modified spontaneous emission (MSE). The enhancement of the EM field is a combination of the excitation field and the Raman scattered field, which is proportional to the fourth power of the field enhancement given by equation (7), where M _Loc_ (ω _L_) is the local field enhancement, M _Loc_ (ω _R_) is the re-radiated field enhancement, │E _loc_ (ω _L_)│ ^4^ is the magnitude of the local electric field amplitude, and │E _loc_│ ^4^ is the magnitude of the incident electric field amplitude.

$$M_{\mathrm{Loc}}\left(\omega_{L}\right).M_{\mathrm{Loc}}\left(\omega_{R}\right)=\frac{{\left|E_{\mathrm{Loc}}\;\left(\omega_{L}\right)\right|}^{4}}{{\left|E_{\mathrm{inc}}\right|}^{4}}$$ (6)

The maximum EM enhancement for isolated silver or gold nanostructures/nanoparticles was observed in the range of 10 ^9^-10 ^10^ ( Le Ru et al., 2007). This was further improved to approximately 10 ^11^ by roughening the substrate surface ( Camden et al., 2008). However, some significant limitations associated with electromagnetic enhancement include the near-field effect of isolated plasmonic nanomaterials that require close presence of the analyte on the substrate ( Chou et al., 2012), heterogeneous signal enhancement ( Schlücker, 2014) on the substrate surface that can alter the reproducibility, and amplification of background signals that obscure the weak Raman signals ( Larmour et al., 2012), thereby reducing the signal-to-noise ratio (SNR) of the SERS system. A low SNR was associated with low sensitivity, poor selectivity, fluorescence, higher Rayleigh signal scattering, and low reproducibility and reliability. Thus, some considerations for transcending the low SNR of a SERS system include the synthesis of plasmonic nanoparticles with uniform shape, size, and morphology to ensure sharp absorption peaks for signal enhancement ( Berciaud et al., 2005), use of pulsed lasers with short duration that minimize photobleaching of the sample ( H. F. Zhang et al., 2007), use of longer-wavelength lasers ( Liu et al., 2020) and spectral filters ( T. Murphy et al., 2000) to reduce fluorescence of biological samples, and minimizing photobleaching effects by optimizing the laser exposure time and laser power. Ongoing studies suggest that the use of these strategies can effectively improve the SNR of SERS systems and their applicability for on-site sensing in environmental monitoring, characterization, and diagnostics. SERS systems provide significant amplification of the scattered Raman signal, thus allowing trace-level fingerprinting in complex samples with a relatively simpler optical system that allows for point-of-use (POU). Hence, the basic building blocks of the SERS system warrants a discussion.

## 3. Optical assemblies for POU-SERS

The building blocks of a SERS system are similar to those of a Raman system with simpler optics, allowing for the construction of portable point-of-use (POU) sensing setups. However, the optimization and alignment of the elements used in the assembly of a POU SERS sensing system, which includes a laser, sample illumination system, photodetector and its associated electronics, and allied optics, is often tailored to a specific substrate of interest.

### 3.1 Sample illumination setups and associated optics

Lasers are commonly employed in modern Raman spectrometry because of the high coherence necessary to produce efficient Raman scattering with high SNR. However, the design of POU SERS systems mostly uses laser photodiodes because of their small footprints. Table I presents a list of laser diodes along with their corresponding emission wavelengths and samples that are typically tested using the respective SERS systems. Laser diodes are a common choice for light sources in SERS systems because of their high stability, electronic tunability, and wavelength precision. However, the choice of a laser diode in a POU-SERS system requires a comprehensive understanding of the plasmonics of the nanostructure or nanocomposite. Additionally, the physical and chemical properties of the substrate and the optical properties of the substrate material can determine the required laser power, laser diodes with low fluorescence, minimal photodamage to the target analyte, and match the substrate plasmonic resonance with the excitation laser. Hence, SERS systems with laser diodes can generate accurate and reproducible measurements.

**Table I.** List of commonly used laser diodes, their emission wavelengths, and the commonly detected target analytes.

Sl. No.	Type of photodiode	Emission wavelength (nm)	Typical power used	Remarks	References
1.	Blue photodiode	488	10-100 mW	Commonly detected analytes are florescent dye or biomarkers tagged nucleic acids and proteins. These diodes possess lesser penetration depth and photodegrade biological samples.	( B. Zhang et al., 2011) ( Pauchard et al., 1999)
2.	Green photodiode	532	10-300 mW	Commonly detected analytes include organic molecules, dyes, and pesticides. It can photodamage biological and non-biological samples.	( Kerwin & Remmele, 2007)
3.	Red photodiode	633	0.5-50 mW	Commonly detected analytes are biological samples, such as nucleic acids and proteins. It is an excellent choice for biological samples, due to low fluorescence.	( W.-Y. Lu et al., 2024) ( Buschmann et al., 2000)
4.	Near infra-red (NIR) photodiode	785	50-500 mW	Commonly detected analytes include inorganic materials and organic molecules of biological and non-biological origin. Higher wavelength visible diodes offer excellent Raman scattering with minimal fluorescence in biological samples.	( N. Li et al., 2023) ( Weinstain et al., 2020)
5.	Near infra-red (NIR) diode pumped solid-state laser	1064	50-500 mW	Commonly detected analytes include minerals, inorganic materials, and carbon-based materials. These lasers do not fluoresce, but generate poor Raman scattering than visible lasers.	( J.-J. Wu et al., 2019) ( Jiang et al., 2020)

The choice of excitation wavelength is a key consideration, as some solvents and colored sample matrices absorb the incident light or the Raman scattered radiation, thus requiring multiple wavelength laser sources. As discussed in Table I, the laser diodes are wavelength-specific, and higher-wavelength lasers can produce a different color with doubled frequency, such as the NIR (1064 nm) laser diode, which can produce green light at 532 nm with doubled frequency. Other considerations for the choice of laser are (i) absorbance and autofluorescence of the sample matrix, (ii) optimized laser power to avoid the generation of fluorescence and sample degradation, and (iii) inexpensive, compact, and easy to integrate with the sensing system.

SERS sample illumination systems are equipped with laser diodes, as in the case of Raman systems; however, the choice of diode is based on the plasmonic absorbance wavelength of the nanostructure used in SERS systems. The sample illumination system also includes other optical components such as a beam expander to regulate the laser beam divergence, collimation optics to ensure a constant diameter of the laser beam, a dichroic mirror that separates laser light from the Raman-scattered light, and a few optical filters that ensure specific transmission of Raman-scattered light to the photodetector. The use of sample illumination systems for POU-SERS sensing applications requires device miniaturization by integration with nanostructure specific laser diodes or fiber optics probes coated with nanostructures, or integration with microfluidic channels for efficient transport of samples to the SERS-active substrate. In addition, the miniaturization of all optical components, such as mirrors, lenses, and spectral filters for specific applications, can also ease device miniaturization. They differ from conventional sample illumination systems in terms of compactness and portability, ease of integration with mobile electronic devices, ease of sample handling and transport to the laser transmission path, and the use of tailored SERS-active substrates for improved sensitivity and specificity.

**Figure 4.**
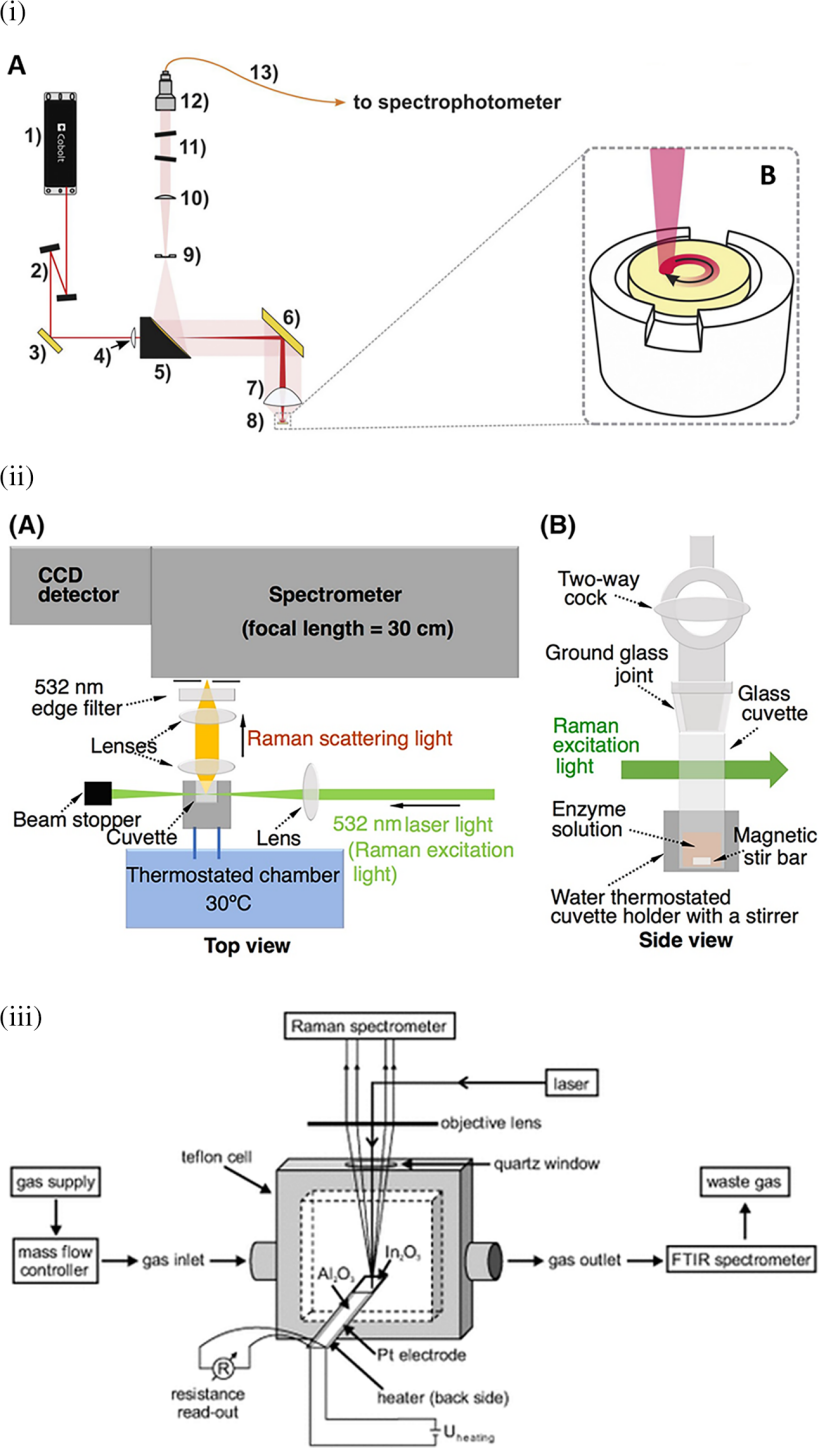
Sample illumination systems used in point-of-use (POU)- SERS devices (i) (A) Optical configuration: 1) 785 nm laser, 2) amplified spontaneous emission (ASE) filters, 3) dichroic mirror, 4) −100 mm meniscus lens, 5) 2″ silver-coated parabolic mirror with 4″ of focus and central 3.2 mm hole in the parallel direction, 6) silver mirror, 7) 2″ aspheric sample lens, 8) sample pellet, 9) 600 μm slit, 10) collimator lens, 11) notch filters, 12) focusing lens, 13) 100 μm optical fiber. (B) Sample holder coupled to the stepper motor for rotating the pellet. (This figure has been reproduced with permission from ( Paiva et al., 2020) Copyright (2020) Spectrochimica Acta Part A: Molecular and Biomolecular Spectroscopy), (ii) Schematic representation of the assay system using Raman spectroscopy for sensing in liquid samples (This figure has been reproduced with permission from ( Kawahara‐Nakagawa et al., 2019) Copyright (2019) Protein Science), (iii) Raman-FTIR device for continuous measurement of the sensor output (DC conductivity) (This figure has been reproduced with permission from ( Sänze et al., 2013) Copyright (2013) Angewandte Chemie).

Raman spectroscopy is widely applied for the analysis of all forms of matter, including the liquid and gaseous phases. Sensing target analytes in solid samples may require manual preparation for accessible target analyte detection, sampling by swabbing or pressing the samples on the substrate, and appropriate instrumentation, such as sample holders or stages integrated with fiber optics or microscopes. Figure 4(i) depicts the optical configuration used for the analysis of solid samples and the staged sample holder attached to the stepper motor that facilitates continuous solid sample rotation ( Paiva et al., 2020). Commonly tested solid samples include nanomaterials, pharmaceuticals, semiconductors, metals, alloys, food, biological samples, forensic analysis, and environmental samples. In contrast, liquid samples are typically sealed in capillaries, glass tubes, or ampoules for samples using volatile solvents or detected in standard cuvettes for aqueous samples, as shown in Figure 4(ii) ( Kawahara-Nakagawa et al., 2019). Typically, in liquid samples, collection optics are placed perpendicular to the sample position for maximum light collection and transmission to the photodetector. Commonly tested liquid samples include organic solvents, acids, biological fluids, petroleum products, chemical reagents, food, and beverages. Consequently, gaseous samples were tested by filling a capillary or small cavity with a fine ground sample. As shown in Figure 4(iii) The gaseous samples are typically passed through specialized optically transparent chambers, where scattering is observed perpendicular to the sample chamber ( Sänze et al., 2013). Some commonly tested gaseous samples include pollutants NO _x_ and SO _x_, molecules that are IR inactive with zero dipole moment, such as H _2_, and hydrocarbons, such as methane, ethane, propane, and butane.

### 3.2 Waveguides for SERS

**Figure 5.**
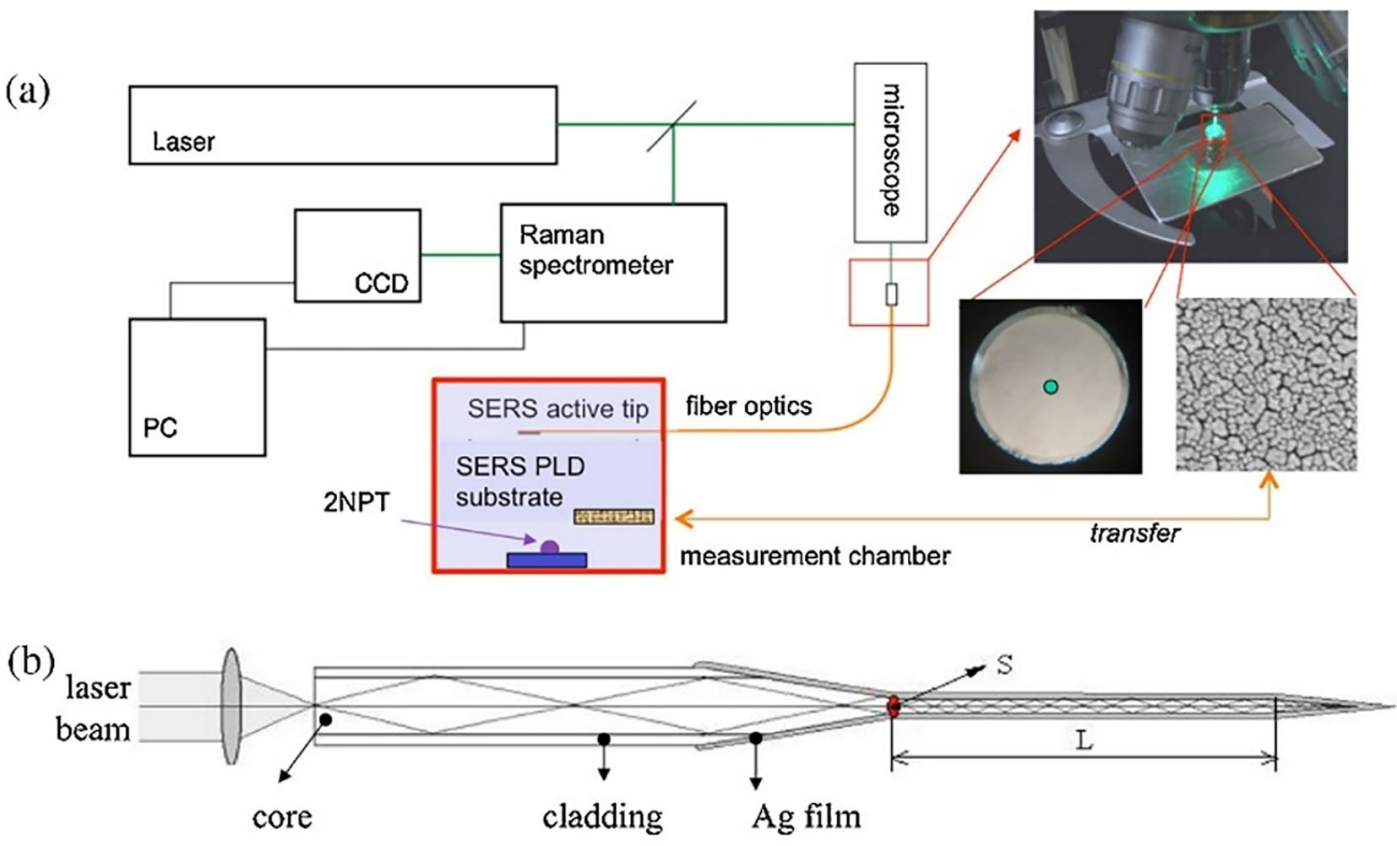
(a) Block diagram of the experimental design of the fiber-optic SERS probe and the Au substrate in the measurement chamber, (b) Schematic illustration of the light transmission through the Ag nanoparticle layered fiber-optic SERS probe (This figure has been reproduced with permission from ( Agarwal et al., 2016) Copyright (2016) Sensors and Actuators B: Chemical).

Optic fiber bundles may be used to guide the laser and collect scattered Raman signals with minimal collimating optics, allowing the construction of simpler and more robust optical systems. The integration of fiber bundles allows for flexible optical transmission; hence, devising systems with SERS-enabled cartridges becomes easy ( Milenko et al., 2020). Typically, a fiber optic probe integrated with a micro-Raman setup comprises an objective lens focused on a laser beam that brings excitation radiation towards the sample. This excitation fiber can be used to illuminate solid samples or immersed in liquid samples. The other terminal collects the scattered radiation from the aperture of the spectrometer. For example, as illustrated in Figure 5, the fiber-optic SERS probe was etched and modified using Ag nanostructures. The SERS-active probe was connected to a microscope objective and the other end was connected to a chamber with nanostructured Au substrates. Evaporation of a 2-napththalenethiol (2-NP) sample with Au nanostructures results in analyte adsorption onto the Ag film-coated SERS-active probe tip, which determines the analyte concentration. Time-based spectral measurements can determine the variation in sample matrix evaporation and thus facilitate the efficient detection of 2-NP ( Agarwal et al., 2016).

### 3.3 Photodetectors

**Figure 6.**
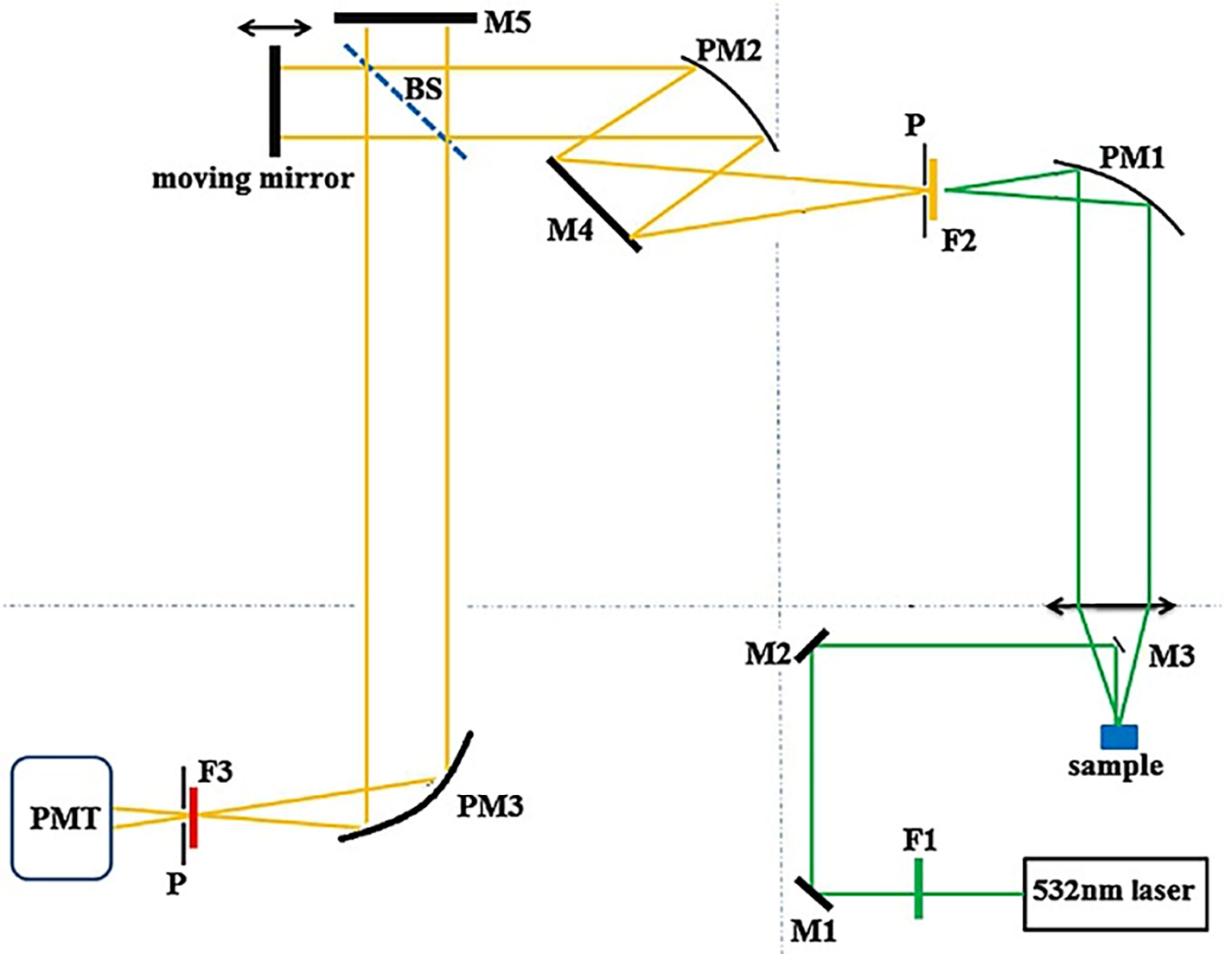
Schematic of the visible laser-based FT-Raman spectrometer (P-Pinhole 2mm diameter, PM1, PM2 and PM3- Parabolic mirrors, M1, M2, M3, M4, and M5- Dielectric mirrors, F1 and F2- Long-pass filters, BS-Quartz beam splitter, PMT-Photo-multiplier tube) (This figure has been reproduced with permission from ( Dzsaber et al., 2015) Copyright (2015) Journal of Raman Spectroscopy).

Photodetectors used in optical systems include CCDs, CMOS, APDs, SiPMTs, and laser diodes, which are comprised of photoresponsive semiconductor materials. They are commonly used because of their spectral range, cost, and rapid capture and analysis. Photodiode arrays are the most commonly used photodetectors for the design of POU systems owing to their scalability and footprint. They offer high sensitivity and rapid responses that are detected in specific visible or near-infrared (NIR) regions. Semiconductor materials with customized bandgaps allow photodetectors to detect predefined wavelengths of light, thereby aiding in specific optical applications. These semiconductors operate based on the difference in bandgap energy, which determines the emitted light absorbance wavelength of the photodetector. Table II. discusses the types of photodiodes, detection ranges, specific characteristics, and commonly detected analytes. However, the tunability of photodiodes for specific applications requires the use of spectral filters for high SNR, optimizing the spectral response with signal processing tools, and miniaturization of photodiodes and allied electronics. Fourier Transform (FT) Raman spectrometers facilitate signal processing of the obtained spectral data by splitting the fundamental constituent frequencies of the signal. It also assists in high-frequency precision, enhancement of spectral resolution, data processing, image reconstruction, and the quantitative analysis of samples. Figure 6 illustrates a visible laser-based FT-Raman system, with optical elements such as parabolic mirrors to focus the excitation light, dielectric mirrors to collect the scattered light, long-pass filters to allow a specific wavelength of scattered light to reach the photodetector, a quartz beam splitter to simultaneously measure the reference and the sample, a pinhole to restrict unwanted light, and a photomultiplier tube that acts as a photodetector. The integration of this Raman system with FT assists in the classification of spectral patterns ( Baeten et al., 1998) and multiplex analyte sensing from complex specimens.

**Table II.** List of commonly used photodiodes, their detection range, characteristics and commonly detected target analytes.

Sl. No.	Type of photodetector	Detection range	Characteristics	Commonly detected analytes	References
1	Silicon photodiode	400-1100 nm	High SNR, excellent sensitivity, and rapid response.	Pharmaceuticals, organic molecules of biological origin.	( Fry, 1975) ( Lozovoy et al., 2023)
2	Indium Gallium Arsenic (InGaAs) photodiode	800-1700 nm	High sensitivity in the NIR region, thermal stability, and minimal dark current.	Food contaminants, biological specimens.	( Moreira et al., 2016) ( Y. Wang et al., 2018)
3	Avalanche photodiode (APDs)	400-1700 nm	Low noise, wide detection range, and high internal gain.	Trace chemical agents and metal ions, biomolecules and quantum dots.	( J. Wu et al., 2012) ( Campbell, 2016)
4	Charge-coupled device (CCD)	400-1000 nm	High SNR, large detection range, and high resolution.	Environmental pollutants, Food contaminants, polymers, and nanoparticles.	( Tsai et al., 2015) ( Q. Zhang et al., 2025)
5	Complementary Metal-Oxide-Semiconductor (CMOS)	400-1000 nm	Low noise, integrated signal processing, and lower power consumption.	Drug formulations, heavy metal ions, chemicals and pesticides.	( K.-W. Li & Yen, 2019) ( Hierlemann et al., 2000)

A significant challenge in developing a POU-SERS system lies in the intermittent cooling of the photodetector to reduce thermal noise and operate at longer wavelengths. The use of spectral filters, such as notch filters or bandpass filters, can prevent heating, and anti-reflective optics may minimize the laser light absorption and thus the heating of the photodetector. At higher temperatures, thermally generated noise can affect the performance of semiconductor materials used as photodetectors, which require intermittent cooling to ensure stability and high spectral resolution. The cooling of the photodetector in a Raman system reduces the dark noise, which originates from the dark current ( Stiff-Roberts, 2011). As these dark currents influenced by the photodetector temperature, which requires intermediate cooling it can aid in reducing background noise and improving the SNR of the sensing system. Most photodetectors employ thermoelectric coolers (TECs) or cryogenic cooling using liquid nitrogen. TECs utilize the Peltier effect, in which a voltage applied across two dissimilar semiconductors creates a temperature gradient. One side serves as a heat sink (at a higher temperature), while the other end cools where the photodetector is attached ( Lundgaard & Sigmund, 2019). In the case of cryogenic cooling mechanism, liquid nitrogen at -196° C is used. A double-walled dewar flask with a vacuum between the two walls is filled with liquid nitrogen on the outer surface of the flask, and the photodetector was placed inside the chamber. The outer surface absorbs heat from the inner chamber until equilibrium was achieved. At equilibrium, the photodetector can operate efficiently at lower temperatures, resulting in reduced dark noise and improved SNR ( Hubbs, 2000), which are crucial for achieving high-quality measurements in Raman spectroscopy and other applications.

### 3.4 Allied optical components

The key components and alignment in Raman spectrometers can be optimized and miniaturized on the basis of the type of sample matrix, sensing application, and deployability. Some essential characteristic features of POU-SERS systems include portability, rapid analysis, multiplex detection, user-friendly interfaces, and customization and integration with intelligence-enabled technologies. Other considerations include the choice of stable excitation with high laser power and minimal photobleaching, the selection of optimal spectral filters, lenses and mirrors with higher NA, and a dispersion component, such as a prism. Spectral filters, such as bandpass filters and notch filters, are employed for light transmission of specific wavelengths. In POU-SERS systems, spectral filters are crucial for blocking Rayleigh scattered light, reducing background noise, and reliable detection. In addition, the high NA of the mirrors and lens assists in efficient light collection and precise focusing owing to their parabolic structure that minimizes the loss of scattered light. Furthermore, dispersion components, such as diffraction gratings and prisms, are used to split the incident light into its spectral components to obtain accurate and high-resolution Raman spectra to differentiate the Raman shift. Precise consideration of these challenges and optimization of the systems can assist in reliable, high-performance, and on-site sensing applications.

## 4. SERS systems for point-of-use sensing

Conventional sensing strategies, such as mass spectrometry (MS), high-performance liquid chromatography (HPLC), gas chromatography (GC), enzyme-linked immunosorbent assay (ELISA), and polymerase chain reaction (PCR), are associated with significant limitations such as high cost, long assay time, need for sophisticated instrumentation, and trained personnel. Hence, there is an immediate need to develop SERS-based POU systems integrated with SERS to aid in rapid sample processing, ease of use, and on-site detection ( Perumal et al., 2021). POU technologies are promising for the detection of whole analytes or their residual form, above the maximum residual limit (MRL) or the lethal dose (LD _50_), which by integration with SERS can provide a typical enhancement of 10 ^6^-10 ^8^ and aid in single-molecule detection. However, designing SERS-based POU systems requires the following considerations: (i) system compatibility and portability, (ii) user-friendly sample handling, (iii) integration with optical accessories and ensuring their compatibility, (iv) maintenance and recalibration without the need for extensive expertise or tools, (v) real-time analysis with rapid data acquisition and results, and (vi) robustness for deployability. A few other considerations include cost-effectiveness, data connectivity with cloud-based platforms, component-durability, and application-specific features. A few recent novel SERS-based POU systems are discussed in Table III, with some critical insights for improvement.

**Table III.** List of SERS-based POU sensing systems for specific analyte detection.

Sl. No.	Analyte	Method	(Bio) receptor	Linker chemistry	Sample	LOD	Remarks	Ref
**Whole cells**								
1	*N. meningitides, S. pneumoniae, H. influenzae*	Parallel hybridization of two cDNA probes, followed by digestion of dsDNA with λ exo-nuclease	ssDNA (cDNA)	Avidin-biotin linker chemistry	-NA-	45.3pM, 99.5pM, 21.7pM	The digestion of reporter probe by λ-exonuclease may not be specific, as the capture probe also contains a terminal phosphate group. DNA digestion may be possible with external factors; hence Raman signal of the dye molecules cannot act as a definitive indicator.	( Gracie et al., 2014)
2.	* E. coli* O157:H7	Competitive interaction of aptameric DNA sequences covalently conjugated to 4-amino thiophenol-gold nanoparticle complexes	Aptamer	Gold-thiol interaction	Ground beef	10 CFU/mL	The use of anisotropic gold nanoparticles of absorbance match of the 1064 nm excitation laser may significantly improve the sensitivity. Sensitivity of the system relied on 4-ATP reporter molecule, an indirect measure of bacteria presence.	( Díaz-Amaya et al., 2019)
**Nucleic acids**								
3	HIV-1 DNA	A novel SERS-LFA-based test strip using MGITC-functionalized gold nanoparticles as SERS nanotags	ssDNA (capture DNA)	Avidin-biotin linker chemistry	-NA-	0.24 ppb	The use of a fluorescent dye can hinder the effect of Raman scatter of the target DNA bound with the cDNA. The excitation wavelength of the laser was not specified which is crucial for light absorption of the plasmonic nanoparticle.	( X. Fu et al., 2016)
**Reporter dye**								
4	Malachite green	Glass fiber and paper-based SERS substrate coated with colloidal silver nanoparticles synthesized by double reduction	-NA-	-NA-	Fish samples	182.5 ppt	Larger pore size of glass fiber paper can decrease the hotspot density, thus affecting the sensitivity. The activity of SERS substrate relies on the pH-based reduction of metal precursor which can be affected by interferents in a real sample.	( Deng et al., 2019)
**Heavy metal ion**								
5	Arsenic (III)	Competitive interaction of aptamer with As (III), thus increasing conc of Au@Ag shell-core nanoparticle, conjugated with 4-MBA reporter dye and adsorbed with As (III) aptamer.	Aptamer	Weak coordination interaction between N atom of nitrogenous bases and Au@Ag nanoparticles.	Lake water	0.1 ppb	Weak non-covalent interactions of the aptamer with 4-MBA can result in partially bound aptamers, affecting the sensor reproducibility. Sensitivity may be affected by the nanoparticle bound free aptamer, as they can hinder the laser to the reporter dye molecules.	( L. Song et al., 2016)
**Food toxins**								
6.	Aflatoxin B1 (AFB1)	Exonuclease assisted hydrolysis of dsDNA (aptamer+ cDNA) resulting in dehybridization of hairpin DNA with cDNA on sputtered gold film.	Hairpin DNA	Gold-thiol interaction	Spiked peanut samples	0.4 ppt	Dehybridization of dsDNA can be affected by the pH and temperature of the sample, resulting in false positives. The specified LoD cannot be achieved with the defined SERS substrate as the λ _max_ of sputtered gold nanoparticles and the excitation wavelength of the laser are distinctive.	( Q. Li et al., 2017)

**Figure 7.**
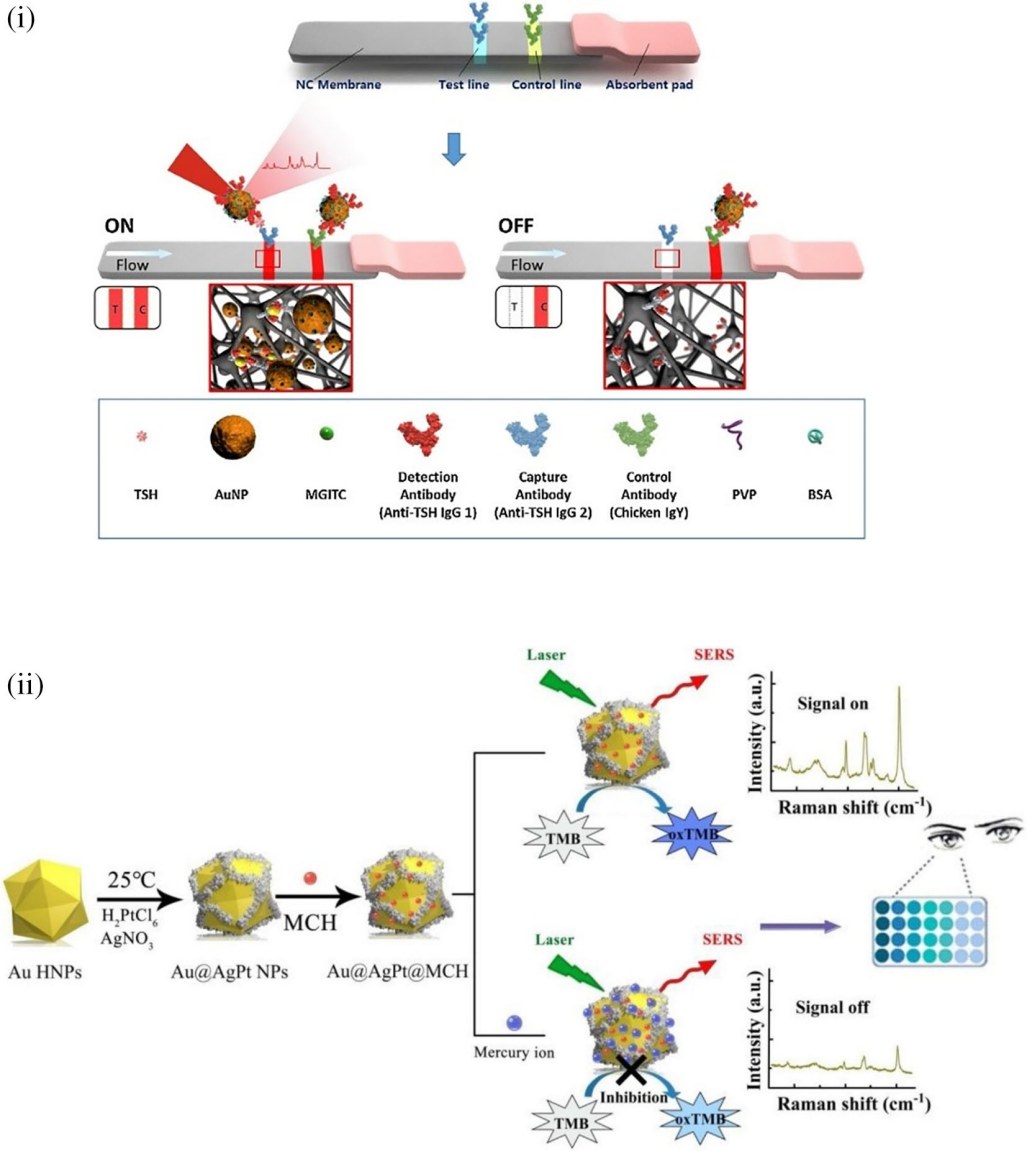
SERS systems for point-of-use sensing (i) Schematic of SERS-based lateral flow immunoassay (LFIA) system for ultrasensitive detection of thyroid stimulating hormone (TSH) (This figure has been reproduced with permission from ( Choi et al., 2017), Copyright (2017) Sensors and Actuators B: Chemical), (ii) Schematic of Au@AgPt@MCH synthesis and the sensing of Hg ^2+^ by colorimetric/SERS strategy (This figure has been reproduced with permission from ( C. Song et al., 2020), Copyright (2020) Sensors and Actuators B: Chemical).

SERS sensing systems are widely used for the detection of analytes, such as metal ions ( C. Song et al., 2020; Y. Zeng et al., 2016), organic ( Ignat et al., 2009; Virga et al., 2012) and inorganic molecules ( Alvarez-Puebla & Liz-Marzán, 2012; Y. Zhou et al., 2012), and small molecules, such as antibiotics, pesticides ( Chan et al., 2003; Severyukhina et al., 2015; P. Guo et al., 2015; Y. Zhang et al., 2014), toxins ( https://doi.org/10.1016/j.foodres.2025.115885) nucleic acids ( Bell & Sirimuthu, 2006; Y. He et al., 2011; Prado et al., 2014), proteins, ( Feliu et al., 2017; Kennedy et al., 2010; Y. Zhu et al., 2020) and radionuclides ( X. He et al., 2019). Most of these POU-SERS systems are efficient for on-site use and have the potential for commercialization. A POU Raman system was developed by Choi et al. in 2017 for the colorimetric detection of thyroid-stimulating hormone (TSH) using a SERS-based lateral flow immunoassay (LFIA). Figure 7(i) shows a schematic illustration of the developed SERS-LFIA platform and the TSH detection mechanism. The image shows the use of SERS nanotags with gold nanoparticles (AuNPs) coated with malachite green isothiocyanate (MGITC), a Raman reporter, and immobilized with an anti-TSH antibody. The LoD of the proposed sensor was calculated to be 0.025 IU/mL in a 10-minute assay and a linear analyte detection range of 1–30 μIU/mL ( Choi et al., 2017). However, the use of antibodies immobilized on gold nanoparticles (AuNPs) can affect the stability and reproducibility of the sensor because the activity of the antibody can be significantly affected by the temperature and pH of the sample. In addition, the use of a Raman dye such as 4-mercaptobenzoic acid (4-MBA) with terminal thiol groups for analyte detection can improve the reproducibility by covalent linking with the antibody.

Song et al. (2020) developed a novel method for detecting mercury (Hg ^2+^) levels in aqueous samples via a unique colorimetric/SERS dual-mode method using SERS-active peroxidase-like Au core-Pt shell nanoparticles (Au@AgPt NPs). Figure 7(ii) depicts the method of Au@AgPt NPs synthesis and laser-induced enzyme-based oxidation for Hg ^2+^. The hexoctahedral core of the Au@AgPt NP had edges coated with Pt, which displayed enhanced catalytic activity and SERS effect. The developed sensor achieved an LoD of 0.28 nM, a linear detection range of 1–5 M with an unaided eye, and 1-10 nM using SERS-active peroxidase-like Au@AgPt NPs. However, this method relies on the catalytic activity of PtNPs and is not specific to the presence of Hg ^2+^; therefore, it can adversely alter the result in the presence of a potent oxidizing molecule in the sample, resulting in false positive results. In addition, masking the PtNPs by any biological or non-biological molecule can inhibit the oxidation of 3,3′,5,5′-tetramethylbenzidine (TMB), thus affecting the sensitivity ( C. Song et al., 2020). This approach may be used for the cumulative detection of heavy metal ions, thus enabling quick discard or remediation; however, for specific detection of Hg ^2+^, receptors such as those illustrated ( Sadani et al., 2019) may help improve sensor specificity and mitigate cross-sensitivity.

Extensive research has been conducted on the development of microfabricated SERS-active substrates, such as those involving nanopillars, nanopyramids, nanoholes, and nanogratings. However, their cost-effective scalability for extensive use is limited and the development of frugal interventions for POU sensing in resource-limited settings is necessary. Thus, commercial-grade disposable materials with surface modifications can be used to develop robust, accurate, and reproducible SERS-active substrates ( Ogundare & van Zyl, 2019). The choice of base materials for use as disposable substrates depends on a few characteristics, such as ease of synthesis and integration of plasmonic nanocomposites, compatibility with the target analyte, resistance to biofouling, and mitigation of noise due to background effects. Commonly used disposable substrates, including paper, fabrics, polymers, and silica-based materials, are briefly discussed. Table III presents the target analyte, substrate synthesis method, use of a (bio) receptor for specificity, linker chemistry of the substrate with (bio) receptor, sample matrix of the analyte, (LoD), and some critical insights for improvement.

## 5. Considerations in design of disposable SERS substrates

Extensive research is being conducted to develop disposable substrates for the sensitive and specific detection of various analytes of interest, as described in Section 6. Despite the use of commercial-grade disposable materials as SERS substrates, the development of SERS sensing platforms that offer repeatable utility is associated with a few important considerations such as hotspot uniformity, surface periodicity, and surface hydrophobicity. The optimizations involved in each of these methods are discussed.

### 5.1 Hotspots

SERS hotspots are localized nanozones with intense plasmonic fields that enhance the resonance and thus the Raman scattering. The interaction of incident light with plasmonic nanostructures results in a highly concentrated and localized EM field, and an exceptional enhancement in Raman scattering is termed a hotspot ( Freeman et al., 1995). This interaction requires the presence of a target analyte near the plasmonic field of the nanostructure. SERS hotspots assist in the amplification of Raman scattering by multiplicative enhancement of the plasmonic field of nanostructures, resulting in synergistic effects and spatial localization facilitating selective enhancement of signals ( Qin et al., 2006) from the hotspot and reduces the background interference ( Le Ru & Etchegoin, 2004). Dyanmics of the structure and orientation ( Mulvaney & Keating, 2000) of the plasmonic nanostructure and their aggregation affects the dimensionality of the nanosubstrate ( Futamata et al., 2003) and thus the signal enhancement. The enhancement factor in the hotspots was calculated as the ratio of the SERS intensity (I _SERS_) to the Raman intensity (I _Raman_) ( Vo-Dinh, 1998). The Raman scattering intensity is mathematically expressed as the square of the electric field energy of the incident light (│E│ ^2^) and expressed as (│E│ ^4^) in the proximity of a plasmonic nanoparticle ( Tian et al., 2002). The generation of hotspots is crucial for the design of SERS materials and platforms to improve nanoscale light confinement ( Hao & Schatz, 2004). Hence, extensive research is being pursued on the synthesis and incorporation of nanomaterials ranging from 0D to 3D for engineering hotspots. Table IV provides an overview of the common nanostructures used in hotspot generation, and some unique properties that assist in higher signal enhancement.

**Table IV.** List of common plasmonic nanostructures used as SERS substrates and their unique features for signal enhancement.

Sl. No.	Material	Unique features for signal enhancement	References
1	Silver nanoparticles	Isotropic nanoparticles with absorbance range of 430-450 nm. Commonly used excitation light sources include Argon or Krypton laser or gallium-nitride (GaN) laser diodes in UV& visible light range, that exhibit excellent signal enhancement.	( Ahmad et al., 2014) ( C.-H. Lu et al., 2014)
2	Silver nanocubes	Isotropic or anisotropic nanostructures with dual plasmonic absorbance modes at 400-450 nm and 800-1000 nm. Commonly used excitation light sources include long wavelength lasers such as Nd-YAG laser and Indium gallium arsenide (InGaAs) photodiodes or Aluminum Gallium Arsenide (AlGaAs) Laser Diodes, that result in good signal enhancement.	( Near et al., 2012) ( Qazi et al., 2017) ( Juodėnas et al., 2025)
3	Gold nanoparticles	Isotropic nanoparticles with absorbance range of 520-540 nm. Commonly used excitation light sources include Krypton laser or GaN or InGaN laser diodes in the visible region, resulting in excellent signal enhancement.	( Hong & Li, 2013) ( Valenzuela-Hernandez et al., 2024)
4	Gold nanorods	Anisotropic nanostructures with dual plasmonic absorbance at 500-550 nm and -750-850 nm. Commonly used excitation light sources include long wavelength light sources such as InGaAs or AlGaAs laser diodes, resulting in high signal enhancement.	( Chang et al., 1999) ( Parchur et al., 2018)
5	Copper nanoparticles	Isotropic nanoparticles with absorbance range of 500-600 nm. Commonly used excitation light sources include standard green, red and amber laser diodes, that result in moderate signal enhancement.	( Kapoor et al., 2002) ( D.-D. Wang et al., 2017)
6	Aluminum nanoparticles	Isotropic nanostructures with absorbance range of 200-400 nm. Aggregates of Al nanoparticles show substantial absorbance in the NIR region. Commonly used excitation light sources include UV excimer lasers and GaN or AlGaN laser diodes, that result in moderate signal enhancement.	( Yu et al., 2020) ( Son et al., 2018)

Hotspot engineering is crucial for the development of SERS-active substrates with nanoscale light confinement in specific localized regions of 0D to 3D materials. In the case of 0D materials, such as quantum dots, a plasmonic field structure with confined dimensionality is not possible ( H. P. Lu, 2005). However, quantum confinement of discrete energy levels can generate localized field that can be engineered to generate hotspots ( Johnson, 1995), and the near-field effect can tailor the plasmonic field magnitude and structure ( Pereyra & Ulloa, 2000). The limited plasmonic field of 0D materials cannot detect larger analytes. Common domains of applications include the detection of small molecules ( Weisbuch et al., 2000) and biomolecules ( Chan & Nie, 1998). In the case of 1D materials, such as nanorods and nanowires, a greater plasmonic field is observed at the tip curvature, which contributes to longitudinal plasmon absorbance ( Clapp et al., 2004). Regularly ordered alignment ( Nikoobakht et al., 2002), surface roughness ( Fan et al., 2004), and tip engineering ( Fan et al., 2004) of 1D materials can be used to tailor plasmonic field structures. These materials are mostly employed for the detection of small molecules ( Kneipp et al., 1998), inorganic molecules ( Niemeyer, 2001), and heavy metal ions ( Muniz-Miranda & Sbrana, 2001) in biological or non-biological samples. For 2D materials such as nanoparticle films, graphene, and graphene oxide, the plasmonic field structure is determined by the nanogaps or crevices of adjacent nanosheets or nanoparticles ( Grigorenko et al., 2012). Cumulative surface plasmon polaritons (SPP) can enhance the local field by coupling with 1D and 3D materials to tailor the plasmonic-field structure ( Zayats et al., 2005). 2D materials are commonly used for the label-free detection of biomolecules. In the case of 3D materials such as microfabricated nanoholes, nanopillars, and nanopyramids, the plasmonic field structure is generated by bulk plasmon resonance ( Wei, 2004). Optimization of the 3D material geometry and customizing the dielectric environment can tailor the plasmonic field structure, and thus assist in hotspot engineering. Three-dimensional (3D) materials are commonly used in the detection of biological and non-biological organic molecules, food sample analysis, and forensic analysis.

***5.1.1 Hotspot engineering with 0D & 1D materials***

**Figure 8.**
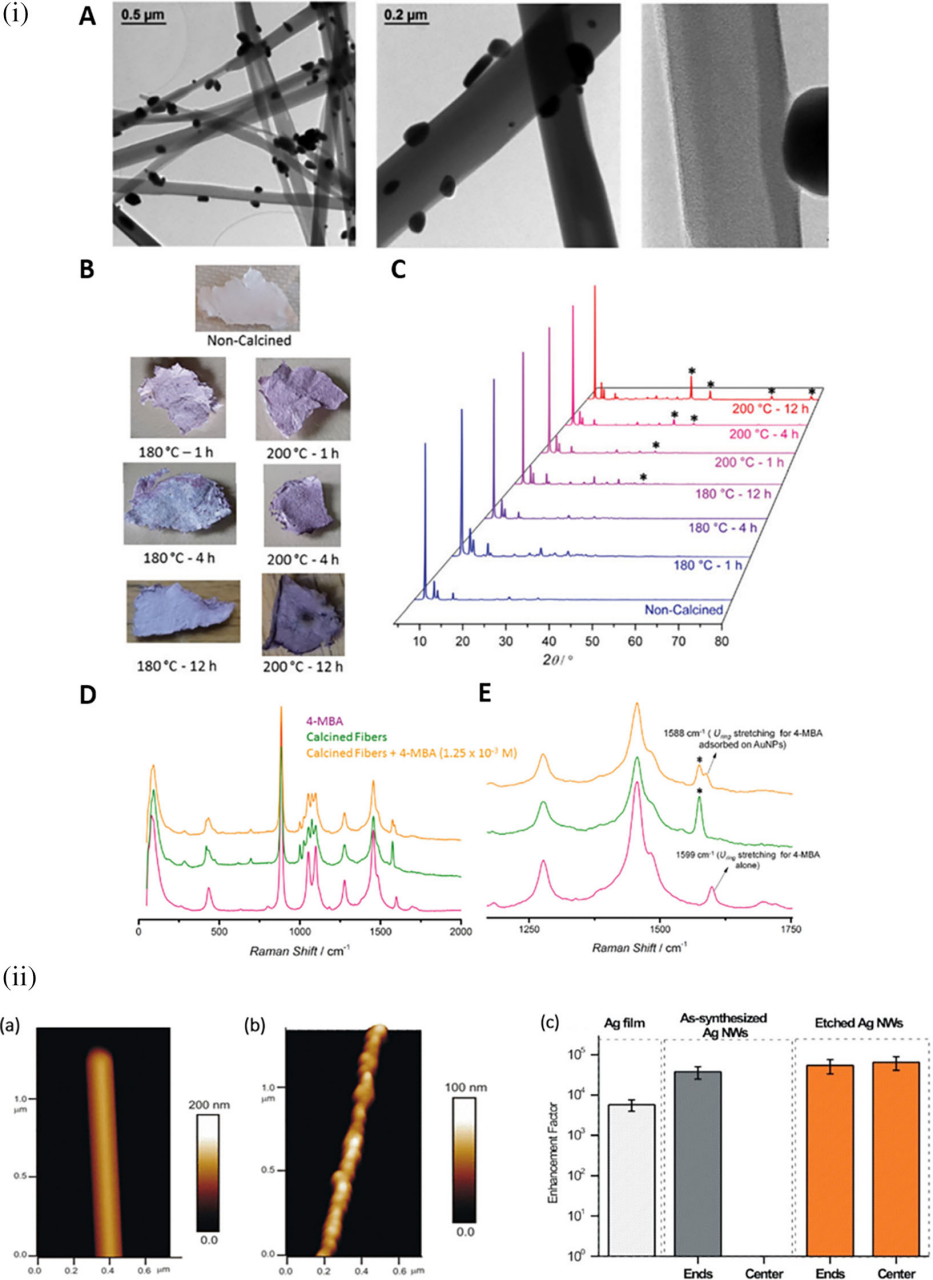
Hotspot engineering with 0D & 1D materials for SERS signal enhancement (i) (A) TEM images of the composite material obtained after calcination of [Au(SPh)]n fibers at 230 ^o^C for 1 hour, (B) Photographs and (C) Powder X-ray diffraction (PXRD) patterns fibers calcined at various temperatures and time durations, (D) Comparison of the Raman spectra of 4-mercaptobenzoic acid (4-MBA) (5 x 10 ^−2^ M), the fibers calcined at 230 ^o^ C–1 h and a solution of 4-MBA with the fibers calcined at 230 ^o^ C–1 h, and (E) a zoomed in version of the spectra. (This figure has been reproduced with permission from ( Vaidya et al., 2020), Copyright from (2020) Journal of Materials Chemistry) (ii) (a, b) Atomic force microscopy (AFM) images of the synthesized and chemically etched Ag nanowires, (c) Average EF per pixel of etched and synthesized Ag nanowires and Ag film. (This figure has been reproduced with permission from ( Goh et al., 2012), Copyright (2012) Langmuir).

Research in the past decades regarding hotspot engineering of 0D and 1D nanostructures has focused on the synthesis of shape-controlled metallic (Au/Ag/Cu) nanoparticles. 0D materials are nanostructures lacking dimensionality and show quantum confinement effects, whereas 1D materials are relatively larger or elongated nanostructures that generate hotspots by phonon scattering and electron confinement. Common 1D materials include nanorods, nanotubes, and nanowires with high aspect ratios that generate well-defined hotspots. These 1D nanostructures exhibit the lightning rod effect by concentrating on the localized EM field at the tip-surface curvature. This results in a longitudinal enhancement mode of the plasmonic resonance ( Cardinal et al., 2017) and absorbance at longer wavelengths (700-1000 nm). Arrays of 1D metal nanoparticles are promising nanoscale optical devices that orient and guide electromagnetic energy ( Quinten et al., 1998). An interesting study by Vaidya et al., the use of free-standing fibers of a Au(I)-based coordination polymer (CP) for 4-MBA detection was described. The [Au (SPh)] _n_ CP flexible fibers are hydrophobic and exhibit high chemical stability under harsh acidic and basic conditions because of the phenyl rings and strong Au(I)–S interactions. Furthermore, calcination can produce a composite, resulting in the formation of AuNPs on CP fibers. Because of the plasmonic resonance of AuNPs, this composite material showed high sensitivity, as demonstrated by SERS ( Vaidya et al., 2020). Figure 8(i) shows the 1D gold(I)-thiophenolate [Au (SPh)] _n_ deposition on a polymer, XRD patterns, and photographs of the modified coordination polymers. Despite the extensive use of electron-beam lithography (EBL)-fabricated 1D metal nanoparticle arrays with defined spacing, the large-scale fabrication of 1D arrays and organized structures is essential for practical applications. Other methods, such as chain assembly synthesis using solution-based protocols, exhibit interesting plasmonic properties. For example, hollow Au nanoparticle-based chains with cobalt nanoparticle (CoNP) chain templates have been assembled using magnetic fields ( J. Zeng et al., 2007). Other examples of nanostructures assembly include the ligand exchange method using mercaptoethyl alcohol (MEA) ( S. Lin et al., 2005) or cetyltrimethylammonium bromide (CTAB), a cationic surfactant ( Dai et al., 2006). For example, as shown in Figure 8(ii), the etching of Ag nanowires using a H _2_O _2_/NH _3_ mixture roughens the nanowire surface resembling beads-on-a-string, which improves the SERS activity across the surface area by 10-fold, while ( Goh et al., 2012) another common 1D nanostructure is face-to-face nanodisk arrays fabricated by on-wire lithography comprising cylindrical nanopores, using porous alumina membranes ( J. Qin et al., 2005). Therefore, research suggests that the decrease in SERS enhancement is due to excitation in the crevices of nanostructures, for which field enhancement is associated with the nanoscale roughness of the metal surface. Despite significant progress in optimizing protocols for nanoparticle hotspot engineering, these nanoparticles do not possess the intrinsic property of serving as efficient SERS platforms because of the limited SERS active area and insufficient EM hotspot strength required for ultra-trace sensing. Future studies should aim to improve nanoparticle efficiency and enhance SERS signals by optimizing the interparticle distance or using nanostructures with high-order dimensionality.

***5.1.2 Hotspot engineering with 2D materials***

**Figure 9.**
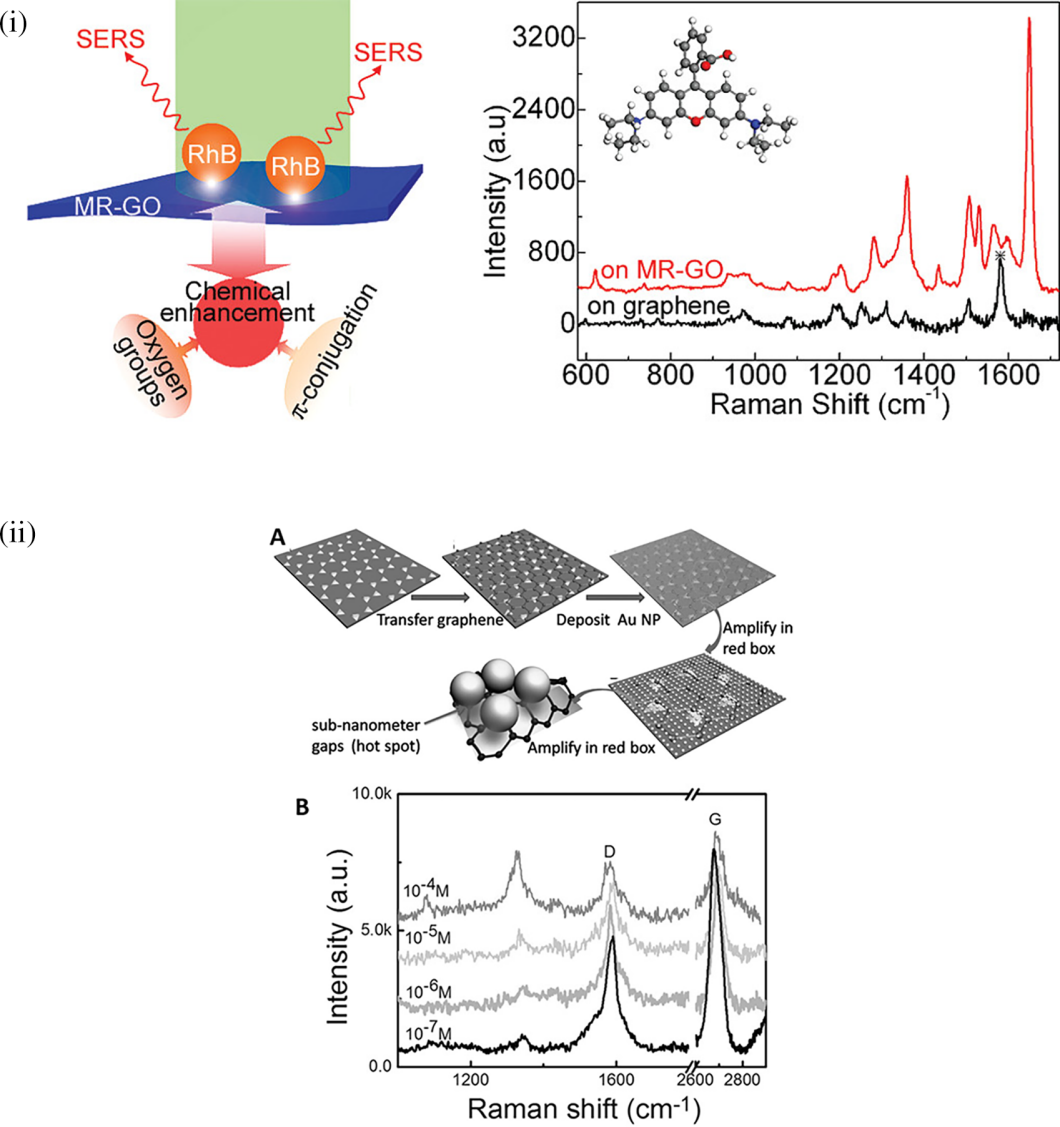
Hotspot engineering with 2D materials for SERS signal enhancement (i) Graphical representation of the 2D mildly reduced-graphene oxide (MR-GO) substrate for Rhodamine B detection and the corresponding SERS spectra depicting increased SERS intensity (This figure has been reproduced with permission from ( X. Yu et al., 2011), Copyright (2011) ACS Nano), (ii) (A) Schematic representation of Au TNAs/graphene/Au NP fabrication. (B) SERS spectra of varying concentrations of 4-NTP absorbed on 9nm thick Au TNAs/monolayer graphene. (This figure has been reproduced with permission from ( X. Zhang et al., 2017), Copyright (2016) Nano micro small).

The organization of plasmonic nanoparticles in ordered 2D arrays significantly initiates the plasmonic coupling of adjacent nanoparticles, generating uniform EMF enhancement. This will likely enable the design of repeatable SERS systems. 2D hotspot engineering can be achieved using a top-down or bottom-up approach. A recent strategy for engineering hotspots with 2D materials is graphene-enhanced Raman scattering (GERS), which results from the deposition of exfoliated graphene on a SiO _2_/Si substrate. The characteristic electronic structure and high electron density of graphene can significantly improve EM interactions ( Schultz et al., 2014). Additionally, the first layer effect of the molecules adsorbed on the graphene surface caused by its high surface area and charge transfer can substantially improve EM interactions ( Ling & Zhang, 2010), thereby enhancing Raman scattering. In a study by Yu et al., mildly reduced graphene oxide (MR-GO) was drop-casted on a 300 nm SiO2/Si substrate for the detection of Rhodamine B (RhB), which displayed a good EF of 10 ^3^ and LoD of 10 ^−8^M. Figure 9(i) Graphical abstract of the 2D MR-GO substrate for Rh B detection and corresponding SERS spectra ( X. Yu et al., 2011). Another interesting strategy is the use of graphene-noble metal substrates that can provide substantial SERS enhancement by the coupling of GERS with the plasmonic effect of nanostructures. An interesting study by Xu et al. fabricated a novel SERS substrate by depositing Ag and Au nano-islands on the backside of a graphene monolayer (1LG) for the detection of R6G molecules on the non-coated side of graphene ( W. Xu et al., 2012b). Furthermore, the geometry of plasmonic nanostructures deposited on graphene was found to alter the nanoparticle assembly. A recent study by Zhang et al., in 2017, demonstrated the fabrication of gold triangular nanoarrays (Au TNAs) on graphene for the detection of Hg ^2+^ in water and sandy soil samples. The use of AuTNAs for substrate fabrication improved the thermal stability and further deposition on the graphene monolayer, which enhanced the SERS signal, facilitating improvement in SERS sensitivity with an LoD of 8.3 nM. A schematic representation of the Au TNA/graphene/Au NP fabrication process and its effect on the SERS spectra is shown in Figure 9(ii) ( X. Zhang et al., 2017). Furthermore, other 2D materials, such as hexagonal boron nitride (h-BN), can also be used for hotspot engineering because of its structural analogy with graphite ( Pakdel et al., 2014). Signal enhancement using h-BN was different from that of graphene, as variations in the charge transfer process of h-BN do not affect the Raman intensity. Kim et al., in 2016, utilized h-BN to insulate Au SERS substrates. R6G Raman signals were stronger for h-BN/Au/SiO2 than for h-BN/SiO2 and Au/SiO _2_ ( G. Kim et al., 2016a). Therefore, the use of nanostructured sheets, graphene, and h-Bn as 2D materials assists in hotspot engineering and the enhancement of Raman signals. Although 2D materials possess unique benefits, the use of 3D materials for hotspot engineering is expected to provide further enhancements.

***5.1.3 Hotspot engineering with 3D materials***

**Figure 10.**
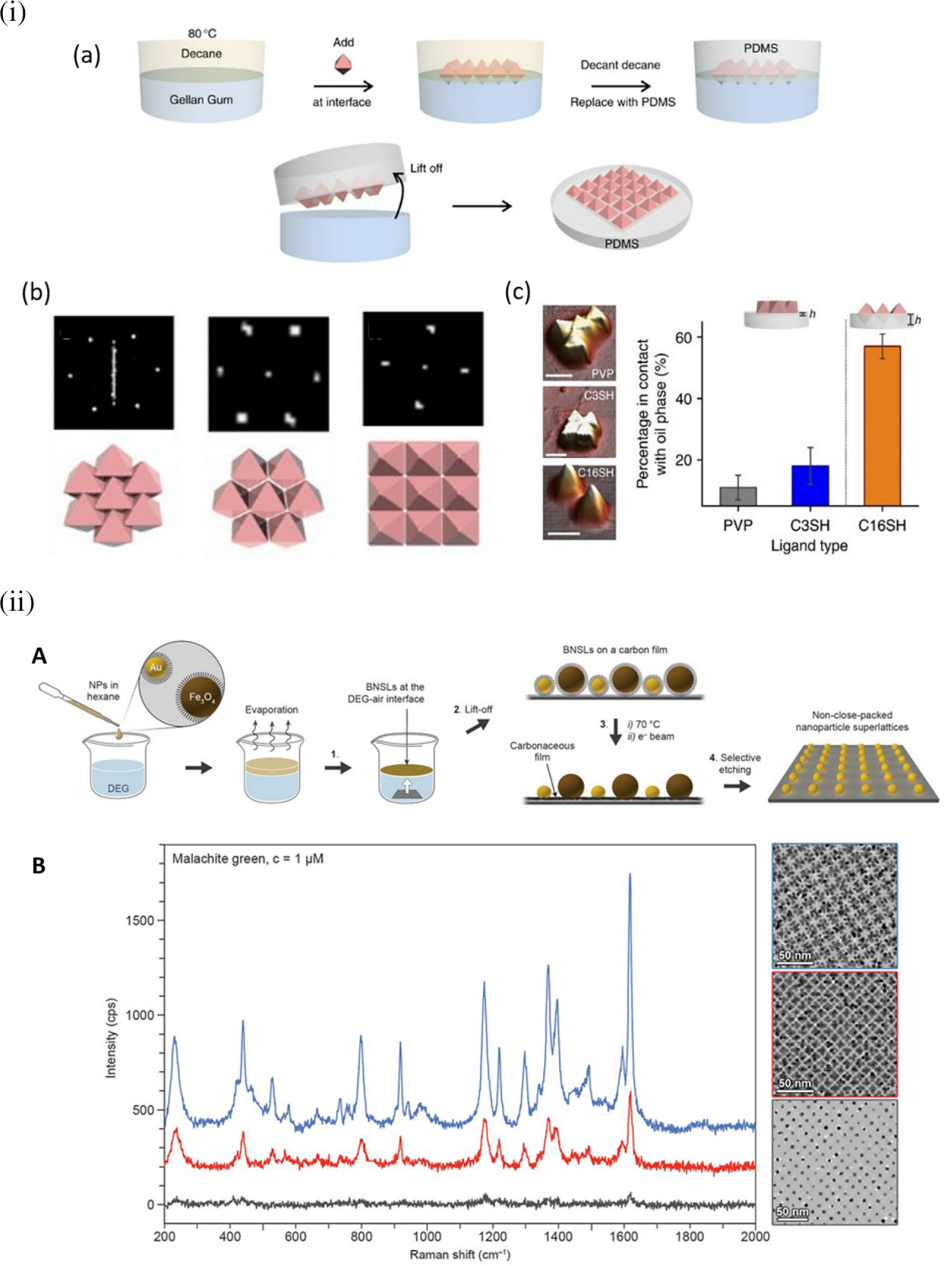
Hotspot engineering with 3D materials for SERS signal enhancement (i) (a) Schematic illustration of the developed interfacial self-assembly near the oil/water interface, (b) adopting a planar configuration, C3-octahedra- octahedra aligned edge to edge, C16-octahedra-square superlattice with a pyramidal protrusions with square superlattice, (c) AFM characterization of PDMS with various functionalized Ag octahedra and the Ag octahedra length (%) immersed into the oil phase (This figure has been reproduced with permission from ( Y. H. Lee et al., 2015), Copyright (2015) Nature Communications), (ii) (A) Schematic representation of the synthesis protocol (B) The measured SERS spectra of *vac*_1_Au _11_ (blue), *vac*_1_Au _5_ (red), *vac*_1_Au _1_ (gray) arrays, as displayed in the TEM images (This figure has been reproduced with permission from ( Udayabhaskararao et al., 2017), Copyright (2017) Science).

Hotspot generation with 3D materials differs from that with 2D materials in terms of dimensionality, spatial distribution, plasmonic field distribution, and accessibility to the analyte. Common 3D materials for hotspot engineering include nanoporous materials, such as nanoholes, nanoarrays, nanopillars, and nanoparticle aggregates. The integration of top-down and bottom-up strategies, as seen in 2D hotspot engineering, was identified to generate open 3D SERS platforms. In the case of a bottom-up approach, nanoparticle self-assembly is widely used to exploit the interface of two immiscible fluids to improve the assembly of 2D nanoparticle meta-crystals. Figure 10(i) shows the schematics of the developed interfacial self-assembly at the oil/water interface, varying configurations of the Ag octahedral nanostructures, Atomic Force Microscope AFM images of functionalized Ag octahedra, and length of octahedra immersed in the oil phase ( Y. H. Lee et al., 2015). 3D hotspot engineering also focuses on designing open structures that improve the accessibility of the laser to the analyte to maximize the SERS response. For example, Udayabhaskararao et al. (2017) used non-close-packed gold nanoparticle arrays with Au and Fe _3_O _4_ building blocks that displayed improved analyte diffusion into the crystal lattice due to the selective etching of Fe _3_O _4_ nanoparticles and the resulting SERS signal enhancement, as shown in Figure 10(ii) ( Udayabhaskararao et al., 2017). Another approach proposed by Lee et al. (2013) used polymeric films for nanoimprint molds to create porous microcylindrical structures and further used metal nanoparticles by electrostatic self-assembly to generate open SERS-active microcylinders with an EF of 6.5 × 10 ^4^ ( S. Y. Lee et al., 2013). The use of 3D porous microcylinders improves the AuNP loading ability, thus improving the SERS signal by 10-fold in comparison with AuNPs on non-porous substrates. Despite the advancements in hotspot engineering, some persistent considerations include: (1) inadequacy of target/analyte detection at the single-molecule level and (2) uniform hotspot density only with specific affinity of analyte molecules to plasmonic surfaces. Hence, most studies still use Raman probes that possess a greater affinity for plasmonic surfaces or larger cross-sectional areas. To overcome the limitations of hotspot engineering, surface fabrication techniques such as in situ growth of 3D nanostructures may be used by electrochemical deposition and bottom-up in situ growth for an enhanced SERS effect.

### 5.2 Substrate hydrophobicity

Hydrophobicity is the intrinsic property of a material to resist water owing to nonpolar interactions, resulting in poor water solubility. Substrate hydrophobicity can alter the contact angle, which defines the ability of the substrate to maintain contact with the liquid sample matrix. Hydrophilic surfaces possess a high affinity for aqueous sample matrices, thus decreasing the contact angle and increasing the surface contact area of the droplet, resulting in rapid sample evaporation ( T. Smith, 1980). In contrast, hydrophobic surfaces have a higher contact angle with the substrate surface because of their quasi-spherical shape and require a longer time for solvent evaporation ( Ko et al., 1981). Analyte detection with hydrophobic substrates generates a large EM enhancement owing to the high contact angle of the target analyte containing the sample matrix. The confinement of plasmonic nanostructures in a sessile droplet on a hydrophobic substrate can generate enhanced SERS signals. This behavior was not observed with hydrophilic surfaces owing to the spread-out nature of the water droplet on them, offering significantly less confinement of plasmonic nanostructures. Hydrophobic surfaces aid in the concentration of target analyte molecules near plasmonic nanoparticles within a confined region on the substrate ( Sakai et al., 2006). The confinement of the analyte molecules with nanostructures generates an intense plasmonic field that assists in SERS signal enhancement. Common hydrophobic surface fabrication methods include silanization ( Péron et al., 2009), fluorination ( Y. Chen et al., 2009), vapor deposition ( Y. Wu et al., 2007), sol-gel coating ( Pilotek & Schmidt, 2003), chemical etching ( B. Qian & Shen, 2005), and the use of hydrophobic nanostructures ( Akagi et al., 2007).

The substrate wettability can be tailored for SERS-based applications by fabricating hydrophobic and hydrophilic surfaces. For example, a microcontact-printing-based hydrophilic surface was fabricated by Shin et al. in 2002 on a hydrophobic polydimethylsiloxane (PDMS) stamp using hydrophilic silver colloids. The hydrophilic nanostructure coated stamp was pressed against a gold-coated Si substrate with a self-assembled monolayer (SAM) of a thiol-containing moiety to develop silver colloidal patterns ( Shin et al., 2002). However, the hydrophilicity of this substrate is dependent on electrostatic and van der Waals interactions, which can severely affect silver colloid adsorption on the gold-coated Si substrate. In another study by Wei et al. (2005), a hydrophilic substrate was fabricated on mica using CTAB-based silver colloids, forming a hydrophilic surface because of the -NH _4_^+^ cationic group of CTAB ( G. Wei et al., 2005). However, CTAB concentrations lower than 10 μM resulted in no SERS signal, and a higher CTAB concentration may result in the formation of surfactant bilayers, resulting in minimal surface adsorption.

**Figure 11.**
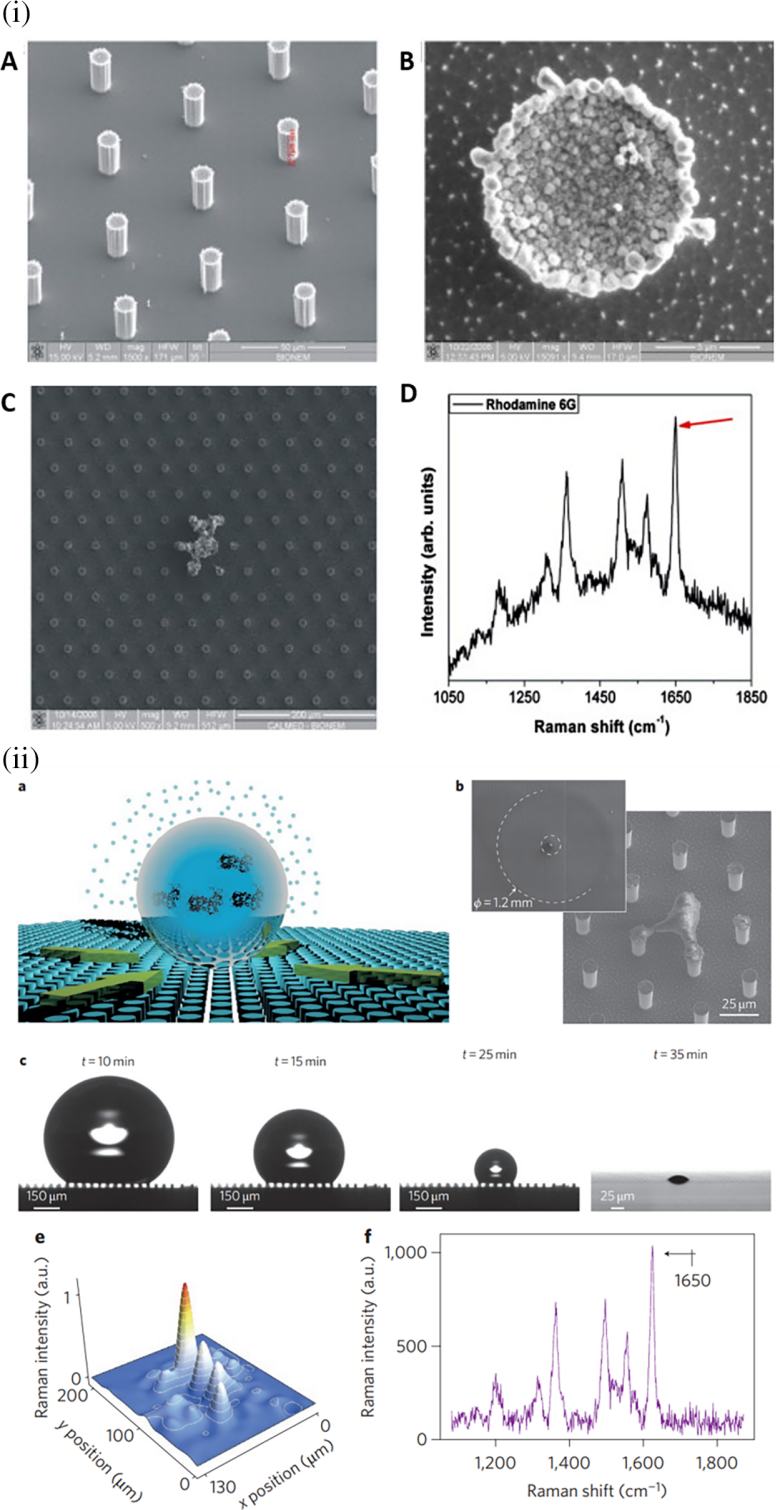
Effect of substrate hydrophobicity on SERS signal enhancement (i) (A-B) MicroPillars of silicon containing ordered hexagonal lattices, coated with electroless grown silver aggregates, (C) SEM image of the residual Rhodamine 6G (R6G) by the end of solvent evaporation, and (D) Characteristic Raman spectrum of R6G. (This figure has been reproduced with permission from ( Gentile et al., 2010), Copyright (2010) Microelectronic Engineering), (ii) Graphical illustration of a droplet and its evaporation at specific solute deposition spot due to the hydrophobic substrate surface. (b) SEM images of the drop diameter and the solute suspended on the nanopillars. (c) Recorded contact angles during evaporation at varying time intervals, (e) Measurement of Raman map of rhodamine and (f ) the associated Raman spectrum (This figure has been reproduced with permission from ( De Angelis et al., 2011), Copyright (2011) Nature Photonics).

As discussed, hydrophobic surfaces can improve the retention time because of the increased contact angle with the sample matrix. An interesting study by Kyle C. Bantz and Christy L. Haynes in 2009, demonstrated the use of SAMs of alkanethiol and perfluoroalkanethiol on the silver film over nanospheres (AgFON) substrates for the detection of polychlorinated biphenyls (PCBs). Cleaned copper discs were deposited with silica nanospheres and vapor-deposited with Ag to form a 200 nm thick Ag film on the nanospheres. Further, it was treated with 1 mM decanethiol (DT) and perfluorodecanethiol (PFDT) to improve the hydrophobicity of the SAM layer. The sensor demonstrated an LoD of 50 pM PCB within 1 min of 532 nm laser exposure, thus facilitating the distinction of PCBs ( Bantz & Haynes, 2009). However, manual agitation of the silica nanospheres cannot ensure homogeneous layer formation, which affects substrate repeatability. Rather, spin-coating the silica nanospheres and analysis with microscopic techniques, such as atomic force microscopy (AFM), may provide insight into substrate surface homogeneity. In addition, the SAM layer assists in the partitioning of PCBs from organic solvents, such as tetrahydrofuran (THF), rather than from an aqueous solvent. However, this may limit the use of AgFON substrates for on-site applications because they are abundantly found in aqueous environmental samples. An interesting study by Gentile et al. (2010) developed a micro/nanopatterned superhydrophobic sensor to detect and differentiate biomolecules. Figure 11(i) shows silver grains coated with regularly ordered disk patterns comprising cylindrical micropillars on a Si wafer obtained by optical lithography aiding in the SERS enhancement. A thin Teflon (C4F8) polymer film was coated on the Si wafer to ensure hydrophobicity, which increased the apparent contact angle from 150° to 175°. This hydrophobic SERS sensor exhibited an LoD of 10 ^−18^ M with a sample volume of 5 μL of R6G ( Gentile et al., 2010). However, the sensitivity may be significantly affected by the reactive-ion etching process, which in turn alters the diameter and height of the Ag mask. Hydrophobic metal surfaces are synthesized by the construction of a micro/nano-metered structure, followed by surface modification with low surface energy molecules ( B. Su et al., 2010).

The contact angle of the substrate affects the analyte distribution around a plasmonic hotspot and thus determines the effective SERS enhancement. Another property of the substrate surface, superhydrophobicity, can be obtained with contact angles higher than 150° ( Reyssat et al., 2006). Optical lithography-based superhydrophobic surfaces are created to adlayer plasmonic nanostructure arrays by micro/nanofabrication. The structured arrays were coated with a thin Teflon film via reactive ion etching. The deposition of a droplet of aqueous R6G solution on the superhydrophobic surface allows it to concentrate and precipitate to a confined area near the Ag nanostructures owing to substrate hydrophobicity. Figure 11(ii) depicts a droplet of a specific solute deposited on a hydrophobic substrate made of nanopillars. The use of a hydrophobic nanopillar substrate resulted in an increase in the SERS intensity, as shown in Figure 11(ii) and (f ) ( De Angelis et al., 2011). The use of a superhydrophobic plasmonic substrate such as silver-decorated polystyrene (PS) nanotubes is highly efficient, with an LoD of 400 ppt for crystal violet ( Lovera et al., 2014). Jayaram et al. used a hydrophobic Ag-decorated ZnO nanostructure thin film with a contact angle of 163°. The as-prepared SERS substrate exhibited an LoD of 10 ^−10^ mol/L for the detection of R6G ( Jayram et al., 2015).

### 5.3 Substrate periodicities

**Figure 12.**
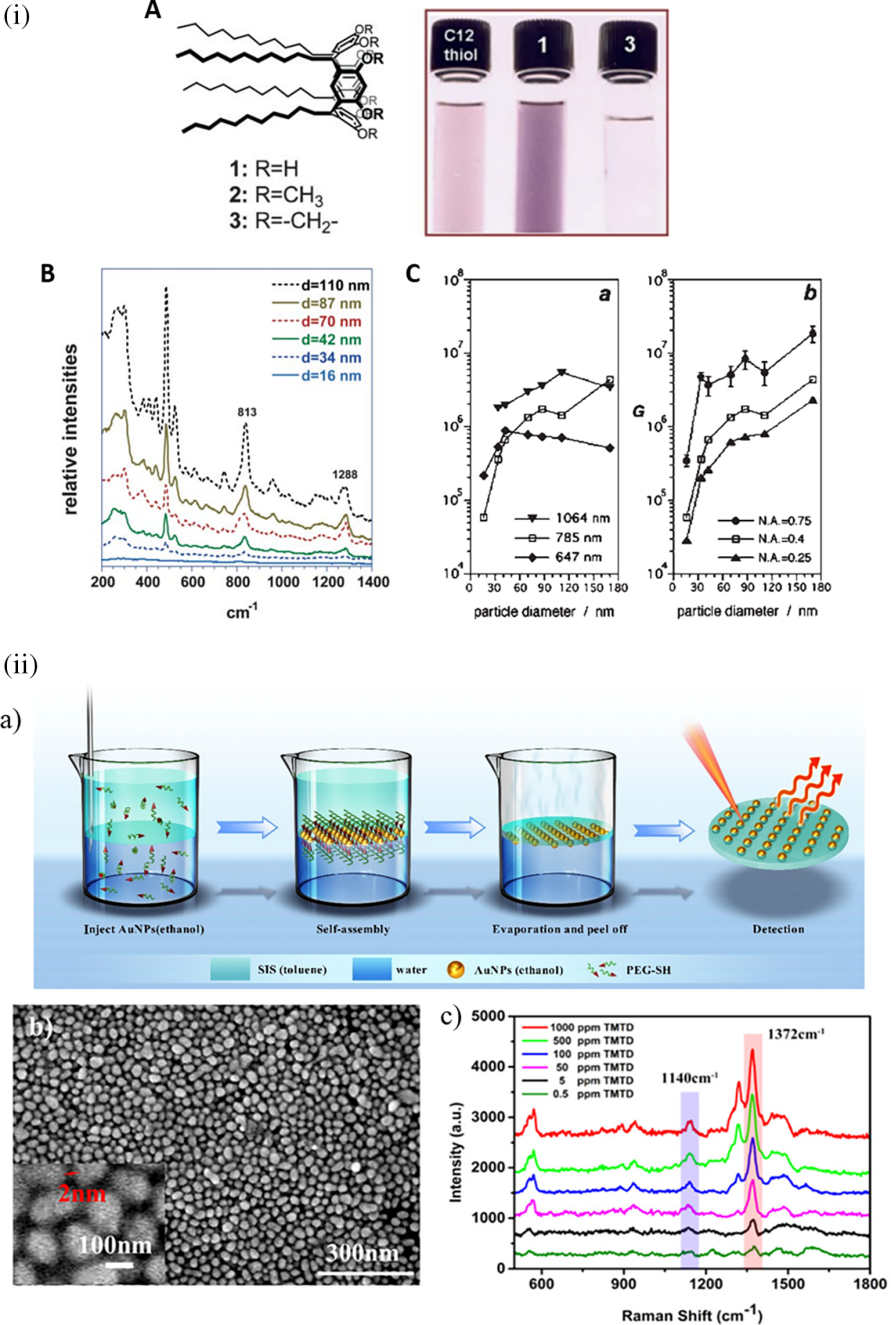
Effect of substrate periodicity on SERS signal enhancement (i) (A) 1-3: C _11_ resorcinarene derivatives (Left); C _12_ thiol capped Au nanocrystals, resorcinarene 1, and cavitand 3 (control), (B) SERS spectra of resorcinarene 6 measured from Au nanoparticle arrays, with a 785 nm laser, (C) (a) G values with excitation wavelengths at 1064, 785, 647 nm; (b) G values with 785 nm using varying solid angles, as determined by the numerical aperture (N.A.) of the collection objective. (This figure has been reproduced with permission from ( A. Wei, 2006), Copyright (2007) Chemical Communications), (ii) (a) Schematic illustration of the self-assembled AuNP metafilm (b) SEM image of the fabricated metafilm with 3ml AuNP solution (c) Thiram SERS spectra (1000-0.5 ppm) obtained from a AuNP metafilm by swabbing the orange surface (This figure has been reproduced with permission from ( N. Yang et al., 2019) Copyright (2019) Langmuir).

Periodicity is defined as a highly ordered array or pattern on the substrate surface that ensures hotspot uniformity, with SERS enhancement up to several orders of magnitude higher than that of disordered metal-nanoparticle films. This section focuses on the use of ordered 1D and 2D SERS substrates and the corresponding SERS enhancements. Periodicity using larger metal nanoparticles is challenging because of the increase in long-range van der Waals forces due to the increase in particle size, thus preventing the formation of 2D periodic structures. The tuning of van der Waals attraction was observed using a proper surfactant, ensuring the close packing of larger nanoparticles. For example, the use of calixarene as a surfactant provides greater repulsive forces for the fabrication of highly ordered larger-sized AuNPs, as shown in Figure 12(ii) ( A. Wei, 2006).

The periodicity of metafilms created by self-assembled nanostructures at the liquid-liquid interface owing to density differences can be utilized for signal enhancement. Metafilms are customizable nanofilms fabricated using precisely structured nanoparticles with unique optical properties that generate highly localized EM hotspots. Yang et al. synthesized a flexible SERS metafilm with self-assembled AuNPs at the water-toluene interface for the detection of thiram, a commonly used moderately toxic fungicide, on orange peel, as shown in Figure 12(i). The metafilm obtained after the evaporation of toluene exhibited high uniformity owing to its ordered nanostructure arrangement. The sensitivity of metafilm was tested using crystal violet as the SERS probe. The sensitivity of the metafilm with 1 mL and 6 mL was low because of the large vacant spaces and due to overspread and close packing, respectively. The metafilm with 3 mL showed the best enhancement, with an LoD of 0.5 ppm thiram ( N. Yang et al., 2019).

In addition to chemical synthesis and self-assembled structures, surface fabrication techniques assist in the formation of 2D periodic substrates. Gong et al. employed plasmonic cavity lens lithography for the fabrication of graphene and silver nanohole arrays for the detection of R6G in standard samples. A 100 nm thick silver film was deposited on a quartz substrate, followed by spin coating of the photoresist and another layer of silver film. The substrate was UV-cured with a chromium (Cr) mask, followed by Ag film removal for photoresist development. The pattern was then transferred to the bottom Ag layer by dry etching. The developed SERS-active substrate exhibited an LoD of 10 ^−11^ mol/L of R6G and an EF of 10 ^7^ ( T. Gong et al., 2019). In another study by Bi et al., an SERS substrate was fabricated using electron-beam lithography (EBL) to detect crystal violet from standard samples. Polyvinylpyrrolidone (PVP) dissolved in ethanol was mixed with chloroauric acid and spin-coated onto the Si substrate. EBL was used to generate nanopatterns of AuNPs on the Si substrate. Polyvinyl alcohol (PVA) gel was then spin-coated onto the nanopatterns. Following the baking and solidification of PVA, the gel was peeled off with the AuNP pattern transferred onto the gel. The fabricated PVA gel with AuNP patterns was used to analyze the sensitivity of the Crystal Violet (CV) probe molecule, which showed an LoD of 10-5 M and an enhancement factor of 9.8 × 10 ^5^ ( Bi et al., 2019). In conclusion, the periodicity of the substrate was observed to improve the surface plasmonic field density and, thus, the EF of the substrate. Furthermore, specific optimizations with physical, chemical, or biological methods may ensure periodicity at the nanoscale, but may not ensure repeatability of the substrate owing to irregular distribution or uneven surface fabrication.

## 6. Disposable SERS surfaces and substrates

Disposable SERS substrates can be used for SERS and are discarded after single use. They are relatively inexpensive and can avoid the issue of biofouling. Unlike reusable substrates, disposable substrates do not require pretreatment steps, as they are intended for single-use detection ( Ferchichi et al., 2015). A few potential disposable substrates employed in SERS-based analyte detection are discussed here.

### 6.1 Paper-based substrates

Paper-based substrates are gaining attention owing to their customizable, biodegradable, and biocompatible properties, and their scalable use in the development of consumer-oriented products. Most paper-based substrates are made of cellulose polymer, which is composed of a linear structure of a few to hundred 1 of 4-linked D-glucose monomers ( Shaik et al., 2022). Other common polymers in paper include hemicellulosic paper ( Kaushik & Moores, 2016; Xiang et al., 2022), lignin-based paper ( Klemm et al., 2005; Mahmoud & Zourob, 2013), and bacterial cellulose-based paper ( Basta & El-Saied, 2009; Xiao et al., 2023). However, hemicellulose exhibits high water solubility, ( Credou & Berthelot, 2014),whereas lignin-based paper undergoes rapid oxidation in the presence of air, leading to degradation of the substrate ( Małachowska et al., 2020). Bacterial cellulose is known to lose its flexibility upon drying, which may serve as a potential limitation for its extensive use in paper-based sensor substrates ( Provin et al., 2021). Therefore, cellulose-based paper has been extensively used for the development of paper-based substrates. For instance, in a study conducted by Romo et al. (2021), the intrinsic properties of cellulosic paper, such as porosity, hydrophilicity, and mechanical strength, were exploited to fabricate SERS-based substrates for cell culture applications. The inherent ability to absorb fluid by capillary action and its porous nature contribute to the adhesion and migration of cells ( Romo-Herrera et al., 2021). These properties make cellulosic paper an excellent substrate for various applications, including the development of paper-based biosensors. In addition to low-cost, large-scale production and disposability are some major benefits of cellulose-based paper ( S. Wang et al., 2012). The physical and chemical properties of cellulose paper have been utilized for the integration of nanoparticles and surface engineering to develop disposable paper-based substrates for SERS-based sensing applications. They are affordable, highly useful in resource-limited settings, and user-friendly alternatives with considerable sensitivity and specificity ( W. Zhao et al., 2008). Additionally, the ease of loading liquid samples will further improve sensitivity by restricting the sample flow to a small sensing region, which will aid in lateral flow assay (LFA)-based sensing techniques ( W. W. Yu & White, 2010).

**Figure 13.**
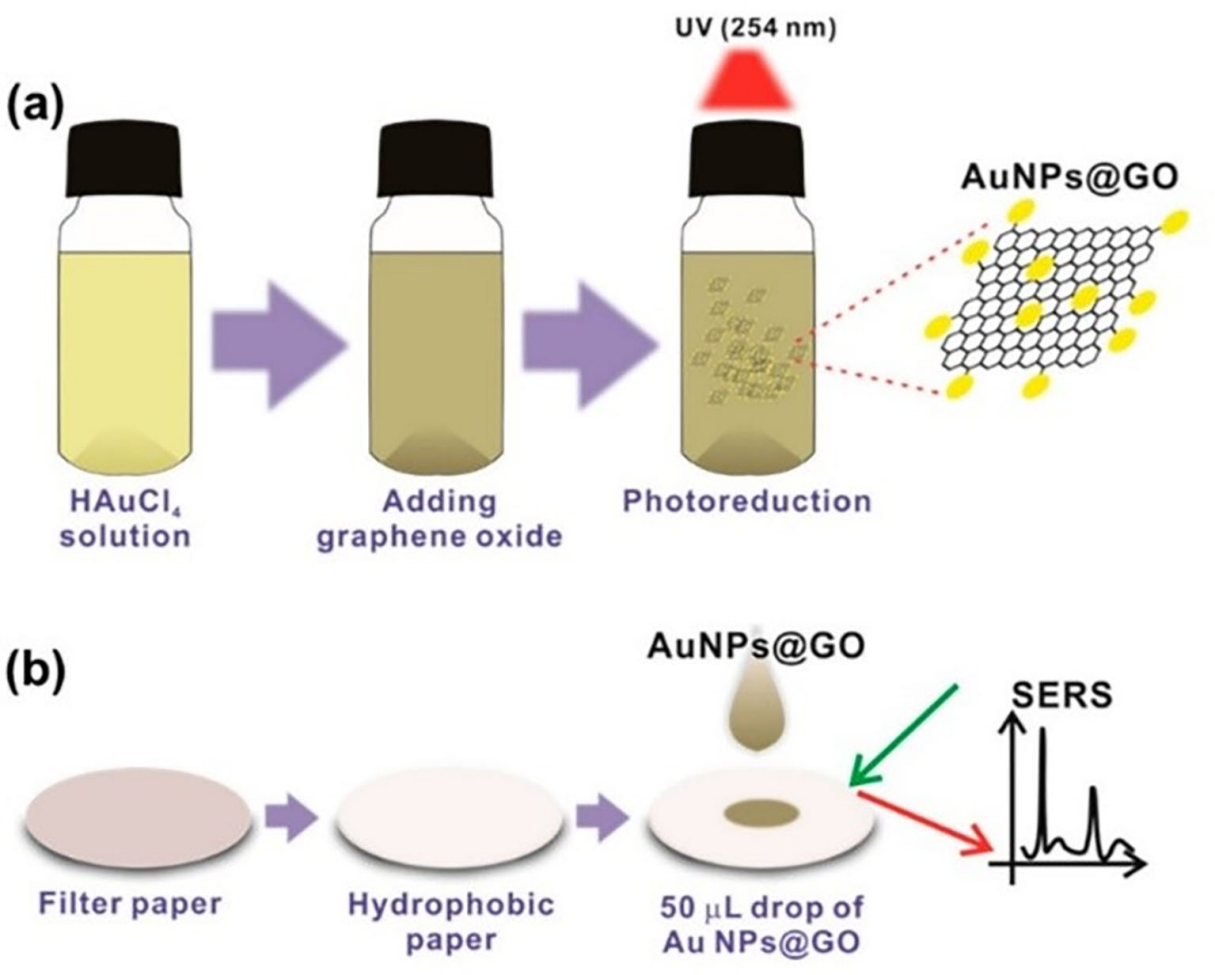
(a) Photoreduction process for the synthesis of AuNPs arranged on graphene oxide (GO) flakes (AuNPs@GO). (b) The hydrophobic (h)-paper-based SERS substrate was fabricated by drop-casting 50 μL AuNPs@GO solution onto the h-paper (This figure has been reproduced with permission from ( Dong-Jin Lee & Dae Yu Kim, 2019), Copyright (2019) Sensors).

In an interesting study by Dong-Jin Lee et al *.,* in 2019, a method was developed for the detection of thiram, using paper as the sensor substrate. Initially, the surface of the filter paper was modified with a diluted PDMS solution to achieve hydrophobicity and confine the porosity, followed by drop-casting gold nanoparticles arranged on graphene oxide (AuNPs@GO) flakes to fabricate a hydrophobic paper (h-paper). The PDMS on the filter paper increased the contact angle (CA) to ~ 128.4° and decreased the surface contact area with an extended retention time of the AuNPs@GO solution. The developed sensor showed an LoD of 1 μM and a linear detection range of 10 ^−3^-10 ^−6^M, using a Raman spectrometer equipped with a 785 nm excitation wavelength (λ _ex_) laser and a laser power of 2.4mW ( Dong-Jin Lee & Dae Yu Kim, 2019). Figure 13 depicts the detailed synthesis of gold nanoparticles (AuNPs) and the steps involved in substrate modification. However, this study did not include validation using real samples. Additionally, the presence of interferents or other structurally analogous molecules can result in false positives. This limitation can be overcome by using a bioreceptor specific to the analyte, which can improve the specificity of the sensor, even in the presence of complex samples. However, all these cellulose-based paper substrates are associated with a major limitation of autofluorescence because of the presence of organic materials, lignin, and additives, such as calcium carbonate (CaCO _3_), alkyl ketene dimer (AKD), polyacrylamide-based resins, bleaching agents, and antioxidants. Autofluorescence may be mitigated by the use of higher-wavelength lasers and optics that block fluorescence or baseline autofluorescence. Other limitations include moisture sensitivity, flammability, and lower chemical and temperature resistance, which can be surpassed by other disposable substrates such as polymer-based and silica-based substrates. Recent advancements in analyte detection using paper-based substrates are presented in Table V.

**Table V.** List of paper-based substrates for SERS-based sensors.

Sl. No.	Analyte	Method	(Bio)receptor	Linker chemistry	Sample	LoD	Remarks	Ref
1.	Mucin-1 in blood	Au NPs are synthesized on a strip of paper fibers by a carbon dots assistant strategy that is used for indirect SERS-based detection of Mucin-1	Aptamer	Gold-thiol interaction	Blood with heparin.	50 ppb	The linear fitting curve show a high relative standard deviation (RSD) that adversely affects the substrate reproducibility. The aptamer used showed poor specificity which can be improved by using a shorter aptamer sequence that reduces complementarity with other biomolecules.	( Hu et al., 2018)
2.	Tyrosine	Immersion-based silver mirror reaction to form silver nanoparticle (AgNP) doped filter paper used to record SERS spectra.	N/A	N/A	Spiked aqueous solution	113.1 ppb	The developed SERS substrate was not specific for tyrosine detection, as no bioreceptor was conjugated to AgNPs, thus generating Raman signatures of other analytes as well. The isotropic AgNPs may not effectively produce the SERS effect, as the 1064 nm excitation laser was used for Raman studies.	( M.-L. Cheng et al., 2011)
3.	Methomyl	Eco-friendly SERS substrate using silver nanoparticles and bacterial nanocellulose paper (AgNP-BNCP) composites synthesized via vacuum-assisted filtration.	N/A	N/A	Fruit peels	58.39 ppb	The results of this study show that the SERS spectra of top-side up and bottom-side up did not vary significantly. But as the optical transmittance of the filter paper is only 1% and that of the BNCP is 50%, the SERS spectra of the bottom-side-up cannot be as high as the top-side-up spectra of the substrate.	( Parnsubsakul et al., 2020)
4.	Melamine & Malathion	Silver nanoparticles used as SERS substrate trapped in the membrane filters	N/A	N/A	N/A	6.3 ppb and 61.5 ppb	The specificity of the target detection can be significantly improved by the use of a bioreceptor. The use of anisotropic AgNPs can improve the sensor sensitivity.	( W. W. Yu & White, 2012)
5.	Sulphite (SO _2_^**-**^)	Integration of gas-diffusion μPAD and SERS on the ZnO-coated paper discs	N/A	N/A	Wine	2 ppm	Sensitivity can be improved by the use of anisotropic noble metal nanoparticles on the ZnO paper discs or by generating hydrophobicity on the paper to concentrate the sample. The use of bioreceptors can improve specific detection of the target analyte.	( M. Chen et al., 2016)
6	Rotavirus	Silver nanoparticles functionalized paper	N/A	N/A	Clinical stool sample	8 ppb	Performance of the developed SERS-active substrate was equivalent with standard rotavirus detection methods. However, decorating the 85 and 100 GSM papers with AgNPs, results in penetration into the bulk, thus affecting the SERS enhancement.	( Biswas et al., 2023)
7	1, 2-bis(4-pyridyl)ethylene	Polyvinylpyrrolidone (PVP) capped silver nanoparticles imprinted paper	N/A	N/A	Artificial urine sample	182 ppm	Fabrication of a low-cost paper substrate with LoD of 1 nM can assist in the further development of highly specific SERS-active substrates. However, the inconsistent performance of the SERS substrate for the 7-day period was not fully understood.	( Chamuah et al., 2017)

### 6.2 Fabric-based substrates

Fabrics can serve as excellent substrates for SERS-based detection owing to their uniform interwoven fibers, durability, and resistance to wear and tear ( Satani et al., 2023). They possess excellent porosity, microfluidic behavior, and enhanced surface area; thus, they ( Ismail, 1991) make ideal materials for efficient contact with samples. In addition, the 300–500 μm coarse fibrous structure in plant fibers ( C. H. Lee et al., 2021) can be modified by coating or trapping plasmonic metal nanoparticles for the development of a SERS-active substrate ( Moon et al., 2011). However, porosity may be a limitation as it may lead to the requirement of large sample volumes, which can be overcome by optimizing the sample microfluidic flow, which is regulated by the capillary pressure and wicking force of the material. Benltoufa et al. suggested that the capillary kinetics in a knitted cotton fabric depended on the geometry of the fibers ( Benltoufa et al., 2008). Furthermore, Bhandari et al. suggested that the wicking rate of yarns is significantly regulated by the number of fiber twists/inch ( Bhandari et al., 2011), while Das et al. suggested that the wicking rate significantly decreased with an increase in twists per inch under the effect of gravity ( B. Das et al., 2011). Other recent studies have suggested that microfluidic flow can be optimized by surface engineering methods such as patterning or channeling the hydrophilicity of fabrics. Furthermore, hydrophobic cotton fabrics can be used for SERS-based applications by dip coating ( Mahltig, 2011), spin coating, ( L. Xu et al., 2012a) or printing with hydrophobic materials ( Noppakundilograt et al., 2010) to obtain varied levels of hydrophobicity that regulate the sample flow. Wearable sensors that can be integrated into clothing can be used for the continual assessment of patient health. Electronic textiles that can monitor physiological parameters are becoming more common, and smart textiles that can monitor chemical biomarkers are required ( Lu et al., 2016).

**Figure 14.**
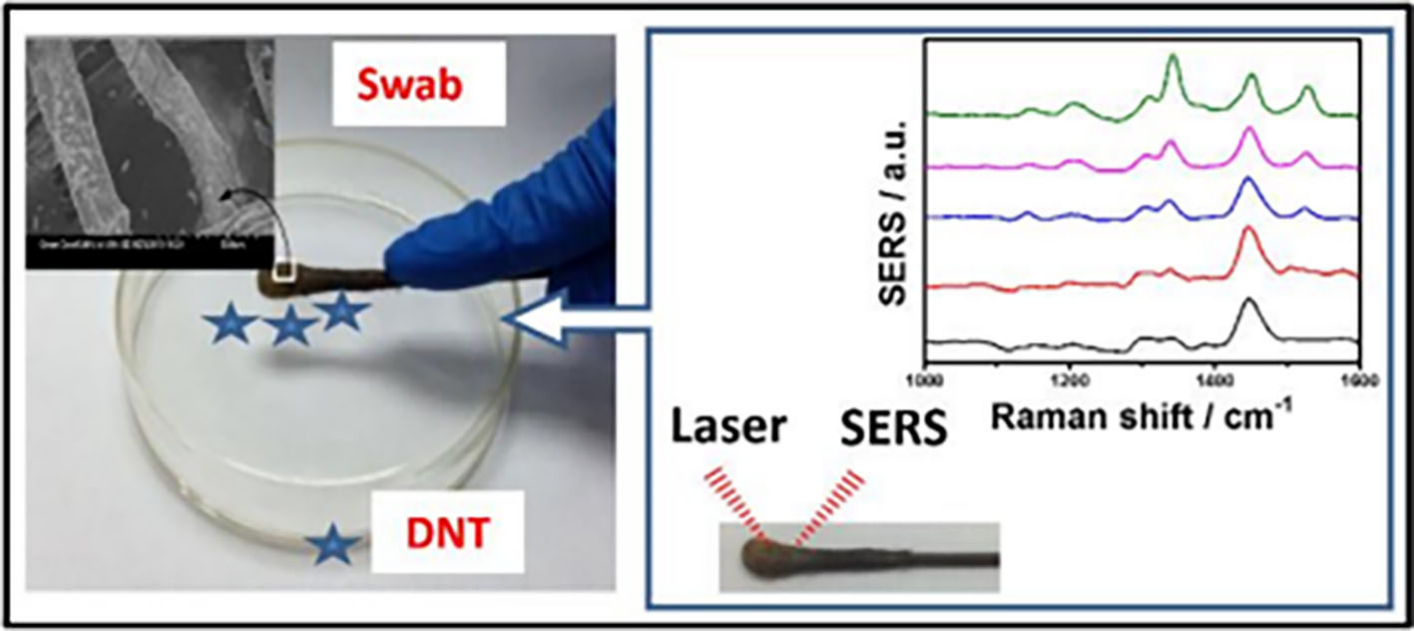
Schematic illustration of the SERS Q-tip on cotton swab for 2, 4-DNT detection and the associated SERS spectra. (This figure has been reproduced with permission from ( Z. Gong et al., 2014), Copyright (2014) Applied materials & Interfaces).

Robinson et al. conducted an interesting study in 2014 for the detection of 4, 4’-Bipyridine (4, 4’-BiPy) using novel fab-chips made of Zari fabric (metal coated over silk fabric). The Zari fabric-based chip is composed of silver nanoparticles (AgNPs) for roughening the Zari yarns, thereby improving the SERS signal. 4,40’-BiPy was used as a probe to assess substrate uniformity and sensitivity, while the detection of adenine bases in DNA provided solid evidence for SERS detection of biological molecules on treated Zari fabric. However, the data suggest a relatively higher relative standard deviation (RSD) for the substrate and large errors at lower analyte concentrations. In addition, no real sample testing was conducted, which can significantly alter the results owing to pH, the presence of other biomolecules, analogous molecules, and other organic loads ( Robinson et al., 2015). In another study, Gong et al. fabricated gel-assembled AgNPs, and their in situ growth on cotton swabs was used for the detection of 2, 4-dinitrotoluene (2,4-DNT) in standard organic solvents, as shown in Figure 14. The cotton Q-tip was transformed into a surface-SERS-active substrate (SERS Q-tip) using a bottom-up strategy. The sensitivity of this direct swab-sensing method was tested with Nile blue A (4-NBA) and further explored for the detection of 2,4-DNT. The swab detection method showed exceptional sensitivity, with an LoD of ∼1.2 ng/cm ^2^ and a shelf life of ~30 days ( Z. Gong et al., 2014). Table VI discusses some recent advancements in analyte detection with exceptional LoDs and some critical insights for improvement in sensitivity. However, most fabric-based substrates do not optimize the microfluidic flow because of (i) varying inter-fiber sizes between the threads and (ii) limited surface area that does not allow printing. Hence, the use of rigid, inert, non-porous materials, such as polymers or silica, can facilitate the fabrication of surface chemistries.

**Table VI.** List of fabric-based substrates for SERS-based sensors.

Sl. No.	Analyte	Method	Bioreceptor	Linker Chemistry	Sample	LoD	Remarks	Ref
1	Carbaryl	Dip coating of triangular silver nanoplates (TSNPs) on cotton fabrics	N/A	N/A	Wet swab sample from apple surface	20.12 ppb	Sensitivity relies on plasmonic nanoparticles on the sensor, thereby displaying the Raman spectrum of other molecules. No cross-sensitivity testing was done using the developed sensor. The specificity of the sensor was not tested due to the absence of a bioreceptor.	( D. Cheng et al., 2018)
2	Thiabend-azole or thiram	Colloid deposition of silver for development of silver-decorated cotton swab	N/A	N/A	Dry swab sample from bitter gourd	1 ng/cm ^2^	The swab-based substrate synthesis showed excellent repeatability, reproducibility, and shelf-life. However, the real samples were not tested with the lowest analyte concentration. Hence 1ng/cm ^2^ cannot be taken as LoD of the sensor.	( Lili Kong et al., 2020)
3	Primary explosive marker 2,4-dinitrotoluene (2,4-DNT)	Ag NPS self-assembly and *In-situ * growing on cotton swabs	N/A	N/A	Fingerprint of contaminated swab	15.41 ppb (p-MBA)	The sensitivity of the SERS-Q tip was significantly high by self-assembly of AgNPs combined with *in-situ * synthesis. The data suggests that the normal Raman spectra intensity of 2, 4-DNT was higher than the SERS intensity obtained after 6 consecutive touches with the contaminated finger, thereby limiting its use for less than 6 times.	( Z. Gong et al., 2014)
4	p,p’-DDT, isocarbophos, sumicidine	Self-assembly followed by *in-situ * growth of AgNPs on polyacrylonitrile fabric	N/A	N/A	Apple surface	5 ng/cm ^2^	SERS spectra of all four tested pesticides were at very high concentrations as shown in the data. However, the concentrations of pesticides on food surfaces will be significantly lower, hence exists a need for improving sensitivity by using anisotropic nanoparticles.	( Cai et al., 2017)

### 6.3 Commercial polymer-based substrates

Polymers are large molecules that consist of long chains or networks of covalently bonded monomers. Some commonly used polymers include polyethylene terephthalate (PET) ( Y. Wang, Jin, et al., 2018a), poly (methyl methacrylate) (PMMA) ( Huebner et al., 2012), (PDMS) ( C. Qian et al., 2015), and polyvinylidene fluoride (PVDF) ( Szymborski et al., 2014). They possess characteristic features, such as durability, flexibility, and versatility, enabling their broad-spectrum applications, including medical diagnostics and environmental monitoring. Recently, the increased use of polymer-based substrates has been observed due to their chemical inertness, disposability, cost-effectiveness, and optical transparency( Z. Li et al., 2020), which facilitate the fabrication of disposable SERS substrates.

However, some major considerations in the fabrication of polymer-based SERS substrates are the flexibility and transparency of the polymers. The flexibility of a polymer ensures design versatility, adaptability with integrated circuitry ( McAlpine et al., 2005), and irregular sample surfaces for proximity between the substrate surface and the analyte molecules ( Restaino & White, 2019). Optical transparency facilitates light penetration ( L. Li & Chin, 2020) and suppression of background fluorescence ( George et al., 2018). Flexible and transparent substrates that can attach conformally to arbitrary solid surfaces are of increasing interest owing to their in situ detection potential. Among these, PDMS ( Park et al., 2017) stands out because of its chemical inertness ( McDonald & Whitesides, 2002), leak-proof nature ( Abidin et al., 2019), gas impermeability ( Nakagawa et al., 2002), and thermal stability( X. Liu et al., 2015). In addition, polymer-based nano composites are gaining attention as hybrid SERS substrates, with a typical composition of synthetic or natural polymers as the host matrix and a filler with 1D nanostructures such as metallic nanoparticles dispersed into a large volume of filler followed by curing ( Connatser et al., 2004; Giesfeldt et al., 2005). Furthermore, substrates modified with surfactants or fatty acids, such as alkyl dithiols ( Kubackova et al., 2015) and oleic acid, ( H. Zheng et al., 2015) have been used as promising disposable-SERS substrates, alongside gold ( Shahar et al., 2017) and silver nanoparticle-doped or modified polymers ( H. Zheng et al., 2015).

In 2017, Singh et al. developed tantalum (Ta)-doped TiO _2_ nanofibers (TNFs) in alcoholic solutions via electrospinning with PVP for the detection of methylene blue. The 5% Ta in the TNFs displayed an improved photocatalytic activity of 2.2 times with solar light irradiation because of the newly induced energy levels in TiO _2_ (Ti ^3+^). These energy levels improve the photoexcited charge division and promote charge transfer, resulting in a higher chemical enhancement of the substrate. However, as the average signal enhancement is primarily influenced by charge transfer, the use of other photocatalytic nanoparticles may improve the sensitivity. In addition, as the homogeneity of Ta in TNFs depends on the properties of PVP and the parameters associated with electrospinning, the repeatability and reproducibility of the substrate may be adversely affected. Rather, PVP electrospinning should be performed prior to Ta doping of the TNFs ( Singh et al., 2017).

**Figure 15.**
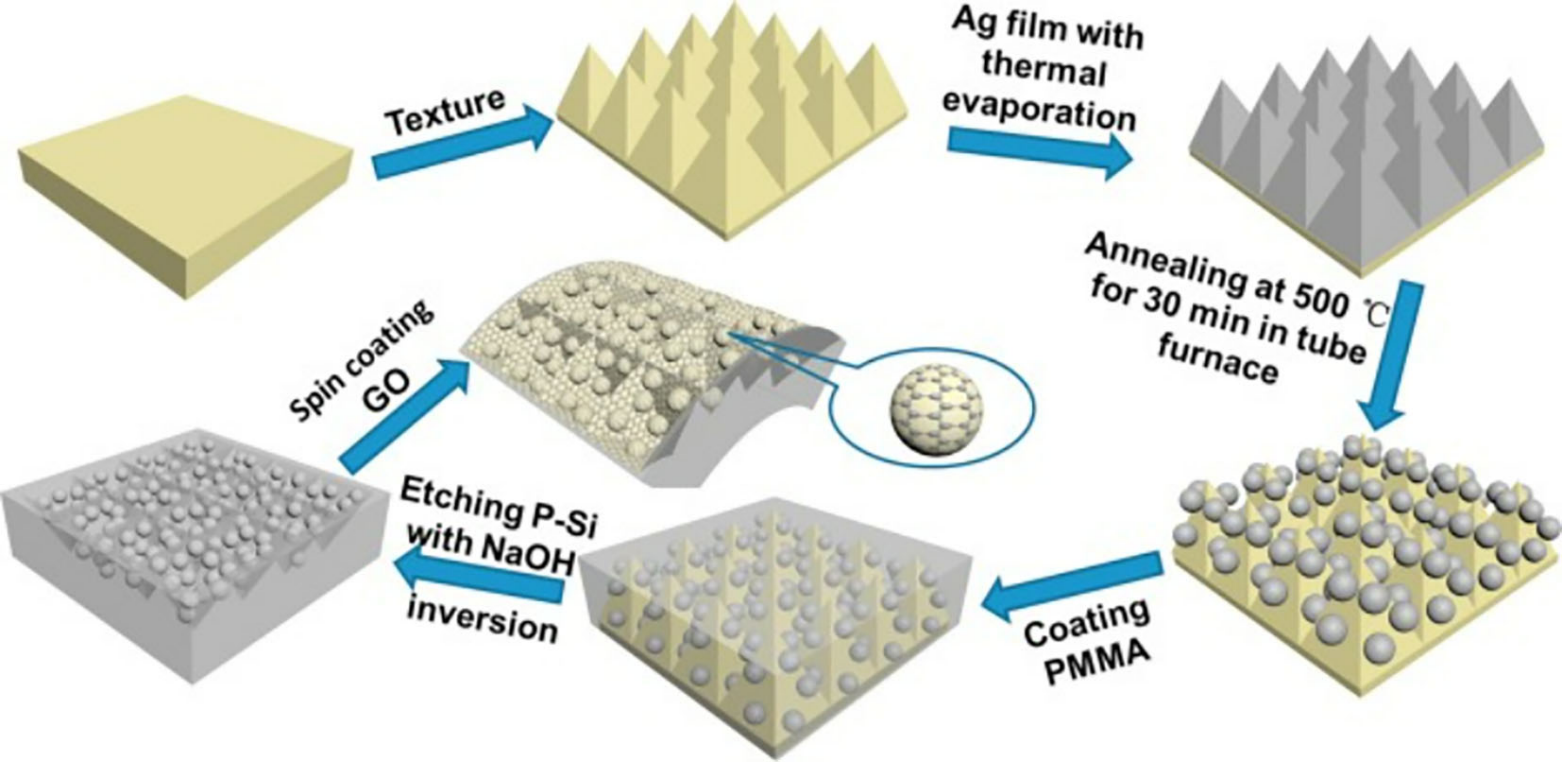
Schematic illustration of the process for the fabrication of the GO/AgNPs/P-PMMA substrate. (This figure has been reproduced with permission from ( X. Zhao et al., 2018), Copyright from (2018) Applied Surface Science).

As discussed above, the flexibility of the polymer is pivotal for its choice as a disposable SERS substrate. Recent studies have shown the increasing attention paid to flexible platforms owing to their durability and adaptability with irregular surfaces and geometries, aiding in practical applications. In a recent study, Zang et al., in 2021, proposed a strategy for the fabrication of a polyethylene terephthalate (PET) film-based flexible SERS substrate using argon (Ar) plasma etching with the physical vapor deposition (PVD) of gold to produce worm-like Au nanostructures. The as-synthesized SERS substrates exhibited a significant signal enhancement with an EF of 1.2 x 10 ^8^ and the LoD was calculated as 10 ^−9^ M. However, the repeatability and reproducibility of the developed sensor substrate were not analyzed. In addition, no cross-sensitivity testing or real sample testing was performed, which can vary significantly with the point-of-use application ( Zang et al., 2021). Another interesting study on the detection of malachite green (MG) presence on fish was reported by Zhao et al. in 2018. They developed a novel method for fabricating a three-dimensional (3D) flexible SERS substrate using graphene oxide/Ag nanoparticle/pyramidal PMMA (GO/AgNP/P-PMMA), as shown in Figure 15. The pyramidal and flexible 3D PMMA film (P-PMMA) was imprinted from pyramidal silicon with a high curvature and triangular geometry; therefore, it acted as an activity site for heterogeneous detection. The larger field enhancement and improved probe capturing are due to the homogenous development of hotspots, as is evident from the higher SERS intensity of R6G in comparison to the flat PMMA surface. The performance of the developed SERS substrate was validated with the AgNP/P-PMMA substrate and GO/AgNP/flat-PMMA substrate using molecular probes, such as R6G and (CV) ( X. Zhao et al., 2018). However, the LoD of the developed sensor varied significantly with the sensing MG on the real sample; therefore, the accessibility of specific detection of MG in a complex organic specimen may be a challenge. The use of a specific bioreceptor may improve the sensitivity and specificity of a sensor, as demonstrated by Li et al. (2018). Biofluid analysis was performed for the specific and quantitative assessment of dopamine in the serum ( L. Li et al., 2018). Table VII discusses a few recent advancements using polymer-based substrates for analyte detection with exceptional LoDs and some critical insights.

**Table VII.** List of polymer-based substrates for SERS-based sensors.

Sl. No.	Analyte	Method	Bioreceptor	Linker chemistry	Sample	LOD	Remarks	Ref
1	*Vibrio parahaemo lyticus*	Surface growth of cysteamine-modified gold nanoparticles on PDMS film by electrostatic adsorption	Aptamer	Gold-thiol linking	Oyster, salmon	12 CFU/mL	Good sensitivity, but the substrate shelf-life was not tested. Poor cross-sensitivity of the substrate shows that aptamer is not specific to *V. parahaemolyticus.*	( S. Wu et al., 2019b)
2	Prostate-specific antigen and α- fetoprotein	Sol-gel synthesis of Fe3O4@TiO2@Ag core−shell NPs and spin-coating of Au nanowires on PDMS film (Au NW/PDMS)	Antibody	Gold-thiol linking	Serum	0.49 ppt & 0.72 ppt	The substrate material may be reusability. The use of antibodies ensures efficient and specific detection at lower analyte concentration and in a real sample.	( Y. Ma et al., 2020)
3	2,6-pyridinedicarboxylic acid	3D plasmonic trap array of snowflake-like silver nanoparticles assembled as flower-like micro-structure	-NA-	-	Bacterial spores of *B. subtilis*	0.2 ppt	The synthesized substrate showed excellent sensitivity, good repeatability and shelf-life. The presence of other pyridine ring-containing compounds in the sample can result in false positives; hence considering multiple peaks can minimize incorrect results.	( Yao et al., 2018)
4	Enrofloxacin hydrochloride	Ag nanoparticles-based SERS imprinting on PVDF membrane by precipitation polymerization method	-NA-	-	Water	39.59 ppb	Despite a good linear detection range, the substrate repeatability and stability were not tested. Generally, MIPs are structure specific and, hence can show poor cross-sensitivity, but in this case the specificity of the substrate was significantly higher.	( M. Wang et al., 2019b)
5	2,4- dichlorophen-oxyacetic acid (2,4-D)	Gold nanoparticle- based biomimetic recognition using magnetic-based molecular imprinted polymer nanoparticles	-NA-	-	Tap water & Milk	0.00147 ppb	The specificity of MIPs is known to be quite low, but integration of the substrate with bioreceptor can improve the specific analyte detection. Selectivity evaluation with other pesticides was not performed with higher concentration of other pesticides	( Y. Xu, Hassan, et al., 2020c)

***6.3.1 DVD-based substrate***

**Figure 16.**
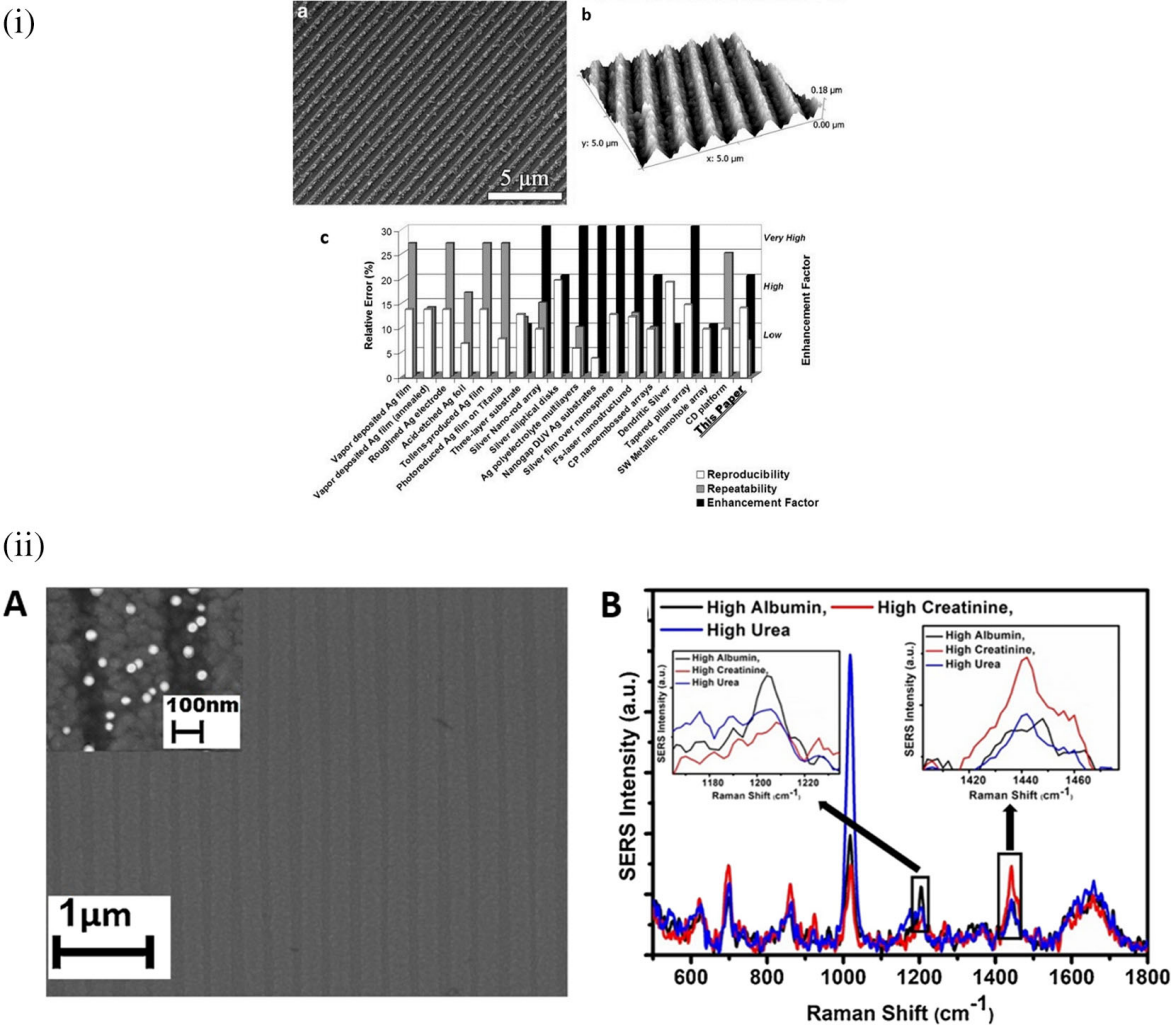
Digital versatile disc (DVD)-based disposable substrate for SERS-based sensing (i). (a) Large-scale SEM images from AgNPs@AgDVD sample, (b) AFM image from AgNPs@AgDVD, and (c) Comparison between literature data of several Ag-based SERS substrates and the results of this study (EF values, reproducibility and repeatability) (This figure has been reproduced with permission from ( Giallongo et al., 2011), Copyright from (2011) Plasmonics), (ii) (A) FESEM image of BR(Blue ray) DVD substrate. The inset image shows the distribution of AuNPs on the substrate. (B) Characteristic SERS signal intensities scattered from the mixture of albumin, creatinine and urea when mixed in different ratio (This figure has been reproduced with permission from ( Chamuah et al., 2019) Copyright (2019) Sensors and Actuators B: Chemical).

Digital Versatile Discs (DVDs) can be efficiently repurposed to generate ordered structures with microscale features that amplify the Raman signal. Commercially available DVDs contain a silver-coated spiral arrangement of rectangular grooves (AgDVDs), which is exploited as a regularly ordered substrate for SERS biosensing. A few characteristics of the substrate include a larger surface area, homogeneity of the substrate, easy customizability, multiplexing, and substrate recycling, which enable the versatility of DVDs for SERS-based sensing. A study proposed by Giuseppe Giallongo et al. detailed the fabrication of SERS substrates based on the electrodeposition of silver nanoparticles on the inner silver surface of a commercial DVD, resulting in AgNPs@AgDVD, as depicted in Figure 16(i). The versatility of the DVD facilitated the customization of the AgNPs and substrate uniformity. The AgNPs@AgDVD substrates showed an enhancement factor (EF) up to 7 × 10 ^5^ with good reproducibility and repeatability. This method provides a practical alternative for inexpensive disposable substrates for SERS and offers further room for improvement ( Giallongo et al., 2011). Commercially available blue ray digital versatile discs (BRDVDs) possess a 320 nm structural periodicity and a channel width of 100 nm, thus generating an ideal structure for entrapping nanoparticles. The BRDVD nanochannel’s sidewalls are composed of polycarbonate (PC) material with a refractive index of 1.58 required for guiding the coupled EM field with the trapped nanoparticles in the channel.

In a similar study by Chamuah et al. in 2019, BRDVD was used as a SERS substrate for the detection of albumin, creatinine, and urea in urine samples. The trapped AuNPs in the BRDVD channel produced a guided mode resonance (GMR) field and an increase in the photon lifetime of the coupled EM field, accounting for the overall increase in the local field intensity, as depicted in Figure 16(ii). The LoDs were calculated as 0.1 μg/mL, 0.2 μg/mL and 0.6 μg/mL respectively, which are well below the normal range and thus meeting the requirements for the analysis in various clinical approaches ( Chamuah et al., 2019). The sensitivity and LoD obtained by the BRDVD-based sensor were significant because only isotropic nanostructures were employed. However, AuNP absorbance wavelength coherence with the 785 nm laser may not be prominent for efficient light scattering from the synthesized nanostructure. The substrate exhibited an exceptional shelf life of 45 d. However, no cross-sensitivity testing has been conducted to ensure specificity in the presence of structurally analogous molecules or other interferents in a complex sample. In another study, Nguyen et al. (2019) demonstrated the detection of amoxicillin by drop-casting AuNPs onto the surface of a DVD. AuNPs were synthesized by pulsed laser ablation of gold, resulting in colloidal gold, which was then deposited on the DVD surface in a circular pattern. The polycarbonate layer was removed from the DVD, followed by rinsing with ethanol and DI water before the deposition of AuNPs. The average EF of the AuNPs/DVD SERS substrates was calculated as 10 ^6^, with an LoD of 0.1 ppm and linear detection range of 0.1-1 ppm ( Nguyen et al., 2019). However, the sensor showed poor sensitivity despite having a good EF with the developed substrate. Rather, the use of anisotropic nanostructures, such as gold nanorods, nanostars, or nanoflowers, significantly improves the detection limit and sensitivity. Despite the advantages offered by polymer-based substrates for SERS measurements, they are also associated with some major limitations, such as optical absorption or scattering, background interference, lower signal enhancement, and incompatibility with different types of sample matrices. Therefore, another promising disposable material, glass, has been extensively used in SERS systems.

### 6.4 Silica-based substrates

**Figure 17.**
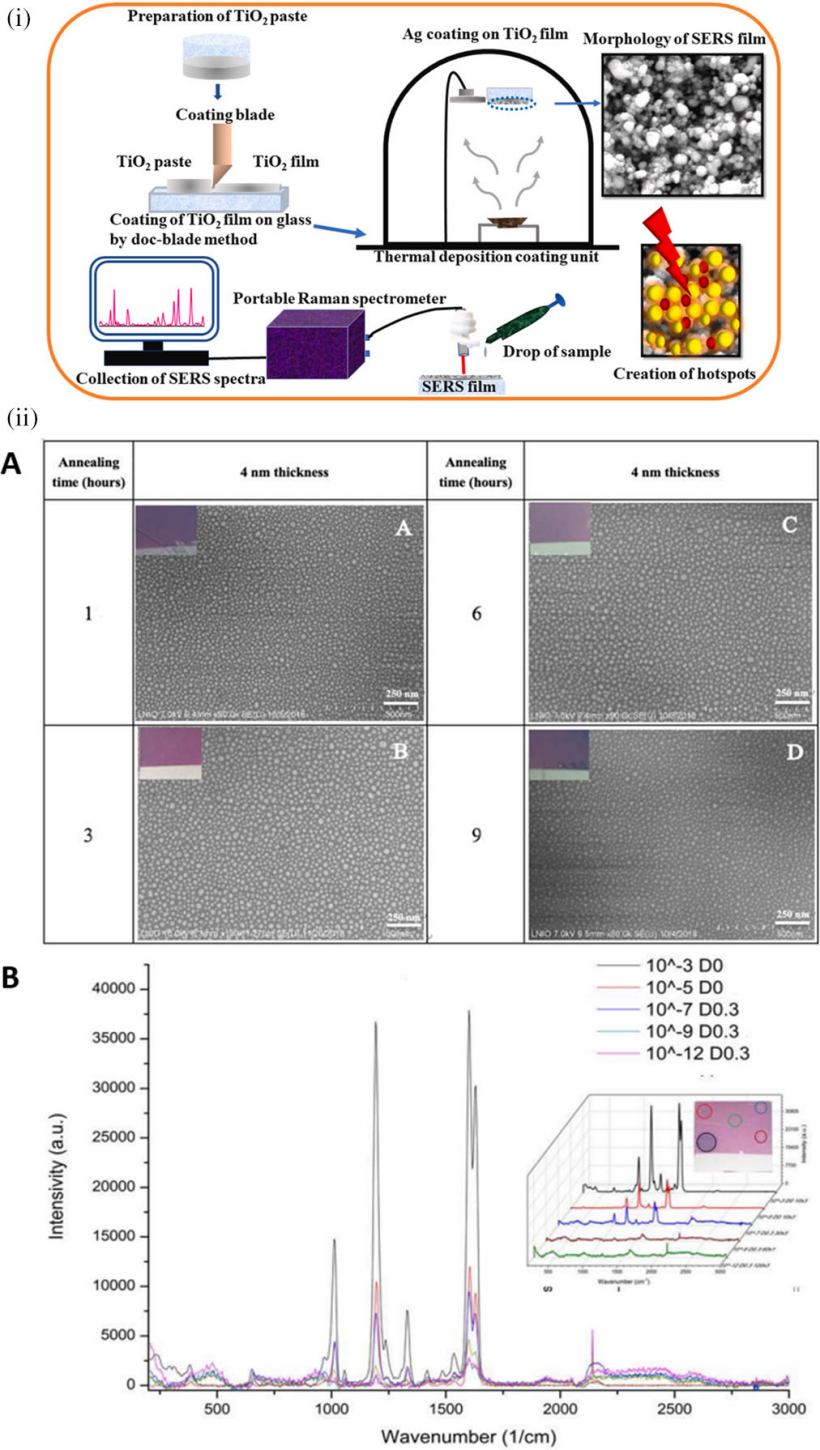
Silica-based disposable substrate for SERS-based sensing (i). Graphical abstract of mesoporous Ag-TiO _2_ nanocage for biosensing application (This figure has been reproduced with permission from ( S. Das et al., 2022), Copyright from (2022) Optical Materials), (ii) (A) SEM images of AuNPs on square glass coverslips coated with 4 nm and annealed for different time periods (a) 1 h, (b) 3 h, (c) 6 h, and (d) 9 h at 550°C, (B) SERS spectra of (1,2-bis-(4-pyridyl)-ethene) BPE molecules of different concentrations (10 ^−3^, 10 ^−5^, 10 ^−7^, 10 ^−9^, and 10 ^−12^ M) using 4 nm gold-coated coverslips annealed at 550°C for 3 h on a hot plate. Inset-photo of a coverslip after the deposition of five different BPE concentrations (This figure has been reproduced with permission from ( L. Zhou et al., 2019), Copyright from (2019) Biosensors).

Crystalline silica, commonly known as quartz, is the most abundant form of silica in nature, whereas other common forms are fused silica or glass, amorphous silica, colloidal silica, and silica nanoparticles. Silica is beneficial because of its high-temperature resistance, chemical inertness ( Vaidya et al., 2020), rigidity ( Bruno & Svoronos, 2010), transparency, electrical insulation ( B. C. Senn et al., 1999), and biocompatibility ( Bayliss et al., 1999). Fused silica/glass slides ensure a stable and inert surface necessary for the chemical modification or immobilization of biomolecules. Conversely, Silica nanoparticle-embedded paper and silica nanoparticle-coated substrates possess exceptional physical and chemical stability and regulate sample flow, which aids in the development of disposable microfluidic devices. These properties will assist in the development of SERS-active substrates for on-site environmental monitoring and biomedical diagnostics. In contrast, porous silica (pSi) is extensively used in adsorption and separation ( Steinbacher & Landry, 2014), (bio) sensors ( Patel et al., 2006), drug delivery ( J.-F. Chen et al., 2004), catalysis ( K. Yang et al., 2015), and environmental remediation ( Nutt et al., 2005), owing to its large surface area, pore size, and sample distribution. However, fused silica/glass-based substrates are extensively used for the fabrication of disposable substrates owing to their characteristic features such as optical transparency, ease of functionalization, homogeneous surface roughness, and biocompatibility. In 2019, Zhou et al. fabricated an SERS-active substrate on ultrathin glass to detect 1,2-bis-(4-pyridyl)-ethene (BPE). AuNPs were annealed on the glass coverslips by metal evaporation at varying temperatures (350°C, 450°C, and 550°C) and at different time intervals (1,3,6, and 9 h), which resulted in varying thicknesses of the gold films, as shown in Figure 17(ii). The variation in the gold film thickness resulted in different colors, such as dark green (8 nm), light green (6 nm), blue (4 nm), and light blue (2 nm). The developed glass substrate showed an EF of 2.71 × 10 ^7^ with an LoD of 10 ^−12^ M and a linear detection range of 10 ^−3^ to 10 ^−12^ M was observed within 120 s of laser incidence on the aqueous solutions. It also showed excellent repeatability with an R ^2^ value of 0.9976 and a good shelf-life of 5 weeks, indicating that these gold coverslips can be a good choice for SERS-based sensors ( L. Zhou et al., 2019). However, coverslips coated with 4 nm Au at 550°C showed the highest surface coverage and smallest interparticle distance, which assisted as a 2D ordered array structure, aiding in better sensitivity. Rather, the use of anisotropic nanostructures can significantly improve the sensitivity owing to the edge effect. In addition, no cross-sensitivity testing or real-time detection were performed because they significantly affect the sensitivity with interference from other analogous structures, biomolecules, or organic load in the sample matrix.

A novel study by Das et al. (2022) fabricated a low-cost mesoporous Ag–TiO _2_ SERS substrate on glass. The distinctive cage-like structure of the TiO _2_ film resulted in the uniform growth of a spherical and porous Ag film with an average interparticle distance of 10 nm, as shown in Figure 17(i), which aids hotspot generation within a small volume. The TiO _2_ nanocage (NC) also increased the effective surface area for analyte adsorption and the Ag–TiO _2_ NC structure displayed an enhancement of 10 ^8^ with the R6G probe using a portable handheld Raman spectrometer. The proposed substrate was recyclable, owing to its photocatalytic activity, upon exposure to UV light for 130 min, which resulted in degradation of the dye molecule ( S. Das et al., 2022). Furthermore, the substrate exhibited high sensitivity for detecting urea concentrations up to 1 mM, which covers the critical range of blood urea levels. In yet another study, Furu Zhong et al., 2018 prepared a silver nanoparticle (AgNPs)-coated porous silicon photonic crystals (PS PCs) for the detection of Picric acid (PA) in alcoholic solution. The developed sensor exhibited an LoD of 10 ^−8^ mol/L and a linear detection range of 10 ^−4^ to 10 ^−7^ mol/L. The PS PCs substrates displayed a 3.58 times greater signal enhancement than conventional single-layered porous silicon. Incubation of PS PCs with AgNPs for more than 75 s decreased the inter-nanoparticle distance and improved SERS enhancement ( Zhong et al., 2018). However, as the PS PCs substrate was incubated with AgNPs, the homogeneity of the SERS substrate was severely affected. Rather, electrospinning or spin-coating of AgNPs on PS PCs can generate uniform hotspots. Furthermore, no cross-sensitivity testing of the AgNP-coated PS PCs was conducted, as it may significantly affect the specificity of SERS-based detection. Therefore, these studies indicate that, despite the challenges associated with the use of silica nanoparticles or glass slides for SERS-based sensing, they can serve as excellent disposable SERS substrates.

In conclusion, the use of all disposable substrates for SERS-based sensing at the small-scale or industrial level was beneficial because of its cost-effectiveness, ease of customizability, portability, and scalability, despite some challenges in substrate performance. Therefore, disposable SERS substrates are promising for sensing target analytes in food safety, biomedical diagnostics, and environmental monitoring, to address real-world challenges.

## 7. Effect of surface modifiers in SERS based sensor design

Despite the intrinsically strong electromagnetic field, SERS substrates can be tailored with specific surface modifiers for higher specificity and robustness. Typically, surface modifiers are nanostructures or nanocomposites linked to substrates to alter the local EM environment and ensure efficient interaction by manipulating the covalent or non-covalent forces between the analyte and substrate. The use of modifiers facilitates hotspot uniformity ( Jahn et al., 2016), easier charge transfer, ( W. Fan et al., 2014) and incorporation of bioconjugation chemistries by improving the surface area of biosensing on the SERS substrate, ( Z. Fan et al., 2013) thereby improving the sensitivity and robustness of the substrate. The substrate surface treatment process categorizes surface modifiers and associated conjugation as physical conjugation, chemical conjugation, and biological conjugation.

### 7.1 Physical conjugation and its effect on Raman enhancement

**Figure 18.**
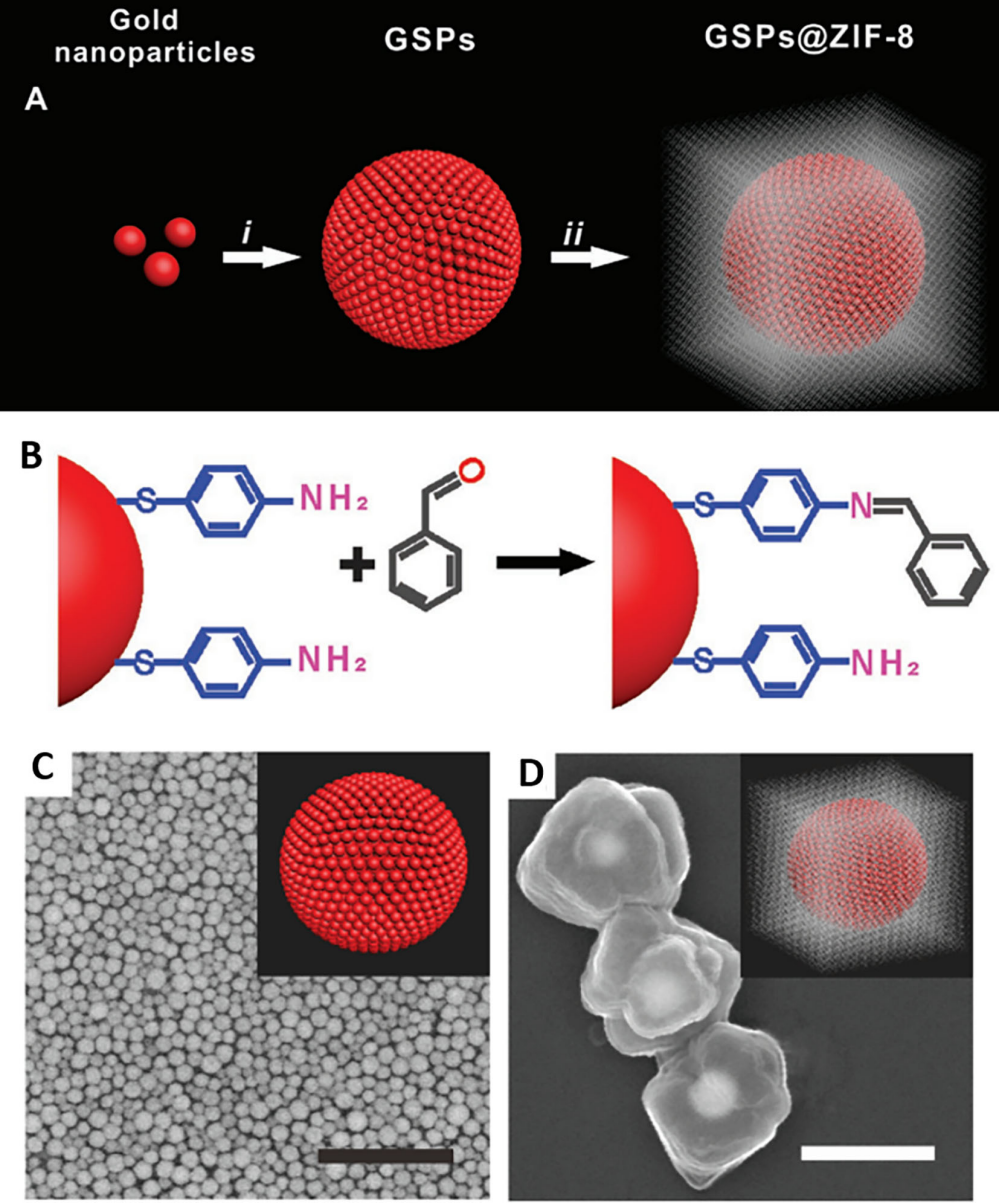
(A) Schematic diagram of AuNP@ZIF-8 synthesis, (B) Schiff’s base reaction of capture aldehyde vapors and covalent linkage with the GSPs. (C) SEM of gold superparticles (GSPs) from monodispersed AuNPs (D _avg_ ≈ 5.8 nm), (D) SEM images of GSPs@ZIF-8 (This figure has been reproduced with permission from ( Qiao et al., 2018), Copyright from (2017) Advanced Materials).

A few commonly used surface modifiers in physical conjugation include metal–organic frameworks (MOF), covalent organic frameworks (COF), aerogels, and hydrogels. These surface modifiers are porous structures that facilitate the controlled integration of plasmonic nanoparticles or Raman-active molecules for the efficient enhancement of the EM field and Raman scattering. The controlled addition of plasmonic nanostructures with defined shapes and sizes to these surface modifiers can generate uniform hotspots on the substrate surface. In an interesting study by Qiao et al., a zinc-based MOF, ZIF-8, was coated with AuNPs (AuNPs@ZIF-8) for the detection of volatile organic compounds (VOCs) for early lung cancer diagnosis, as shown in Figure 18. Regulated gaseous flow of VOCs resulted in increased adsorption owing to the use of ZIF-8. Schiff’s base reaction between the 4-ATP dye on the AuNPs and the aldehyde group of VOCs resulted in a detection limit of 10 ppb ( Qiao et al., 2018). However, the efficiency and sensitivity of the system may be significantly affected by humidity, which may restrict the adsorption of VOCs onto ZIF-8. In addition, the Schiff base interaction may be due to the interaction with another aldehyde-containing interferents. Table VIII presents some recent studies describing the effect of physical conjugation, their effect on Raman enhancement, and some critical insights for improvement.

**Table VIII.** List of commonly used physical conjugations in SERS-based sensors.

Sl. No.	Analyte	Method	Sample	LoD	Physical conjugation	Remarks	Ref
1	Sulfur dioxide (SO _2_)	Wrapping of porous NU-901 on thiol-magenta modified Ag nanoparticles (TM-Ag@NU-901)	Commercial wine	640 ppb	Thiol magenta group of binds to AgNPs, facilitating the alkene (C=C) reaction with the SO _2_.	Interaction of the SO _2_ with the C=C group of AgNPs is not specific and can significantly later with any thiol-containing interferents. Rather, the use of a specific bioreceptor may improve the specificity of detection.	( Huo et al., 2022)
2	β-lactoglobulin	AuNPs doped COF that mimics nitro-reductase activity	Milk and yoghurt	4.26 and 2.75 ppm	Sodium borohydride reduction of HAuCl _4_. 4H _2_O in COF-methanol suspension resulting in regularly doped AuNP-COFs	The mechanism of physical conjugation is stated clearly. But, the coherence of the excitation laser wavelength and the absorbance range of nanoparticle does not match, thus resulting in poor sensitivity. Rather, the use of anisotropic nanostructures can improve the sensitivity.	( Y. Su et al., 2019)
3	Sulfamonometh-oxine	In-situ reduction of AgNPs with β-cyclodextrin	Lab wastewater sample	11.8 ppm	Decorated β-CD-AgNPs into a PVA hydrogel network, with the use of 1,10-phenanthroline as Raman probe	Despite, the optimization for synthesis of PVA gel-CD-Ag composite, repeatability may be a significant challenge, as the analyte is only adsorbed on the gel surface, that may not be enriched with β-CD-AgNPs.	( Ouyang et al., 2015)
4	Trinitrobenzene (TNB), 3-nitro-1, 2, 4-triazol-3-one (K ^+^(NTO) ^-^)	Sol-gel synthesis of titanium dioxide (TiO _2_) wet gel	Standard organic solutions (DMSO/acetonitrile)	21.3 ppb	Adsorption of TNB and (K ^+^(NTO) ^-^) onto the TiO _2_ wet gel.	The use of TiO2 for photocatalytic activity assisted rapid gel synthesis. However, the use of TiO2 for SERS effect cannot be a good choice due to its low plasmonic activity. Rather the use of UV-based AuNSs during the aerogel synthesis can generate high plasmonic field that can significantly improve the sensitivity.	( W. Liu et al., 2023)

### 7.2 Chemical conjugation and its effect on Raman enhancement

**Figure 19.**
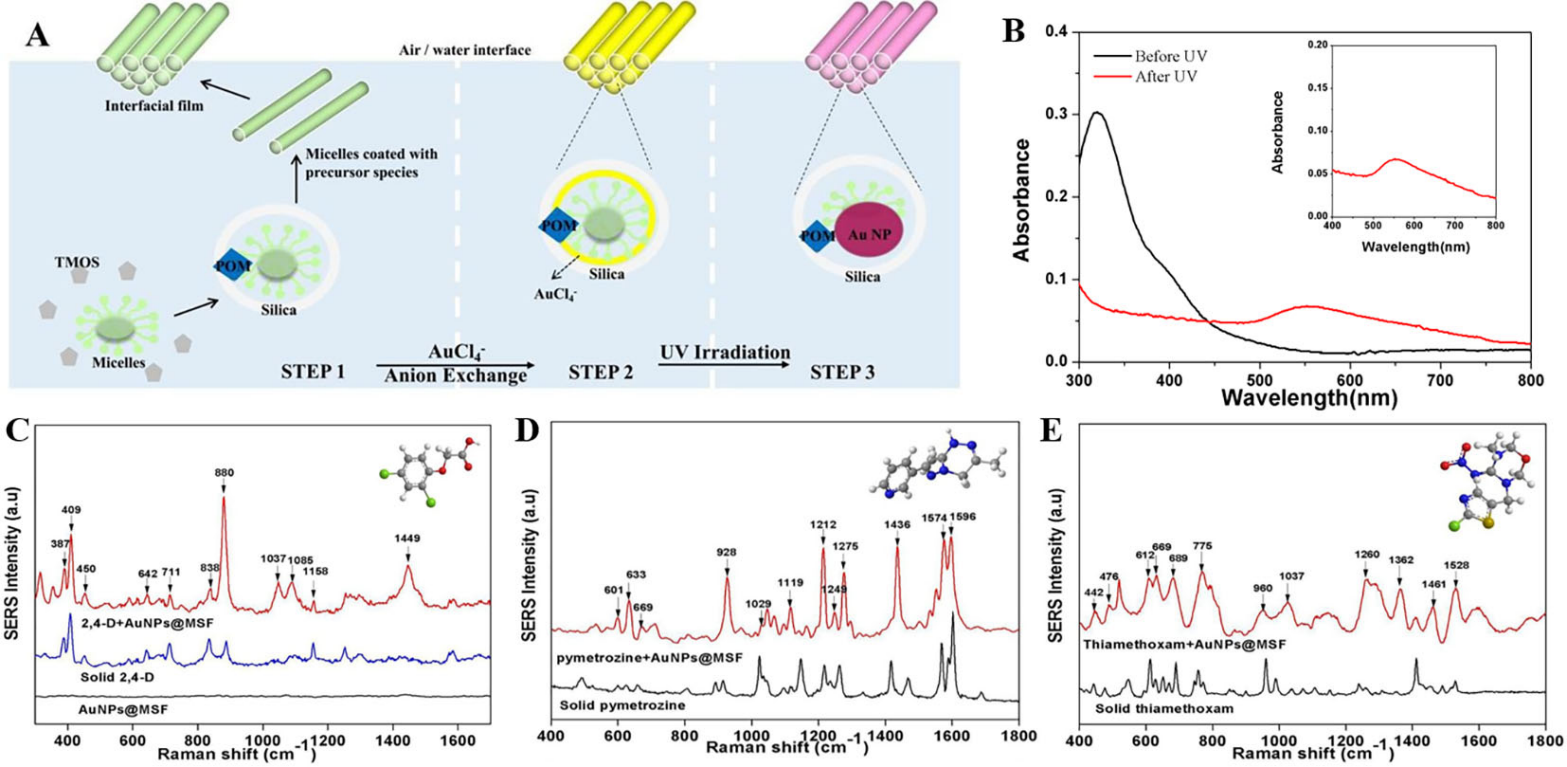
(A) Synthesis of AuNPs@MSF at air-water interface, (B) UV-Visible absorption spectra of AuCl _4_- silica films before and after UV irradiation exposure (C) SERS spectra of AuNPs @MSF mixed with 2,4-D and 2,4-D solid respectively (D) SERS spectra of AuNPs@MSF mixed with pymetrozine and pymetrozine solid respectively (E) SERS spectra of AuNPs@MSF mixed with thiamethoxam and thiomethoxam solid respectively (This figure has been reproduced with permission from ( Y. Xu, Kutsanedzie, et al., 2020b), Copyright (2020) Food Chemistry).

Commonly used chemical surface modifiers include long-chain thiol-containing molecules such as 11-mercaptoundecanoic acid (11-MUA), 1, 4-benzenedithiol (1, 4-BZT), and amine-terminated or carboxyl-terminated dendrimers. Typically, chemical conjugation is used for the immobilization of target analytes or bioreceptors to improve sensitivity, mitigate non-specific interactions, and stabilize the sensor. These techniques can significantly alter the Debye length of the developed sensor ( L. Xu et al., 2020d) and generate uniform hotspots on a substrate. They also act as binding sites for different functional groups, such as silanes, amides, phosphoric acids, and carboxylic acids, relative to the available substrates **.** In an interesting study by Gorbachevskii et al., the local electric field density and the hotspot density were observed to vary depending on the kinetics of Raman dye oxidation with hydrogen peroxide using citrate-capped AuNPs ( Gorbachevskii et al., 2018), while another study by Kim et al., in 2016, observed irreproducibility in Raman enhancement by selective interaction between the amine and carboxyl groups of dendrimers and rGO of the Au-rGO complex, respectively ( K. Kim et al., 2016b). In another interesting study, Xu et al. developed a compact AuNP-templated nanostructure from a mesoporous silica film (MSF) at the air-water interface, as shown in Figure 19. The increased adsorption of AuCl _4_^**-**^ in the MSF channels resulted in the close packing of AuNPs@MSF and facilitated hotspot creation and SERS enhancement. Real sample detection was done in water, milk, and apple samples and obtained an LoD of 0.79 pg/mL for 2,4-D, 1.04 pg/mL for pymetrozine and 1.21 pg/mL for thiamethoxan for a linear detection range of 0.1 to 1000 ng/mL ( Y. Xu, Kutsanedzie, et al., 2020a). Table IX presents some recent studies describing the effect of chemical conjugation, their effect on Raman enhancement, and some critical insights for further enhancement.

**Table IX.** List of commonly used chemical conjugations in SERS-based sensors.

Sl. No.	Analyte	Method	Sample	LoD	Chemical conjugation	Remarks	Ref
1	Enrofloxacin (ENRO), ciprofloxacin (CHL), and chloramphenicol	SERS with dendritic silver nano-substrates	-NA-	20ppb	Electro-static interaction of Cl ^-^ in ciprofloxacin and silver dendrites	Though good sensitivity has been achieved by the as-synthesized substrate, no real sample testing was done. pH, ionic concentration and other factors can significantly alter the analyte detection from a real sample.	( L. He et al., 2010)
2	Tetracycline and Doxorubicin	SERS of chitosan coated Au nanoparticle conjugated with graphene oxide in 3D porous membrane	Lake water and river water	92& 88% captured respectively	Electrostatic interaction of chitosan on AuNP and graphene oxide	The LoD of the developed sensor was not specified, hence cannot be used for POC applications. No cross-sensitivity testing and real sample testing were performed, as they determine the sensitivity and specificity of the sensor.	( Jones et al., 2017)
3	Lindane	A reusable SERS substrate synthesized as nano-porous silver (NPAg) sheet.	-	87ppb	Electrodeposition on nanoporous silver sheet	This method of substrate synthesis showed good sensitivity, repeatability and stability, but real sample testing and cross-sensitivity were not conducted.	( Chi et al., 2020)
4	Aldrin, dieldrin, lindane and endosulfan	SERS	-	45 ppb	Functionalization of gold and silver surface with alkyl dithiols	Dithiols were identified to influence the surface coverage of metal nanoparticles and also affect the affinity of binding with pesticides. A mixture of these analytes in a matrix can significantly affect the affinity of binding between DT8 and the specific pesticide thus producing incorrect results. Also, no real samples have been tested.	( Kubackova et al., 2015)
5	2,4-dichlorophenoxyacetic acid (2,4-D), pymetrozine, and thiamethoxam	SERS	-	0.79 ppt, 1.04 ppt and 1.21 ppt	Compactly packed gold nanoparticles (AuNPs) templated from mesoporous silica film	Good stability with a relative 3% standard deviation.	( Y. Xu, Kutsanedzie, et al., 2020a)

### 7.3 Biological conjugation and its effect on Raman enhancement

Biological conjugation involves the covalent linking of a specific bioreceptor with a substrate surface or modifier to improve sensor specificity. This is primarily dependent on the terminal functional groups of the stabilizing shells. The bio-conjugation of the ligands on the surface of NPs is facilitated by the formation of an amide bond by carbodiimide activation. Because it offers superior stability, bio-conjugation created by thiol group attachment is regarded as a robust and effective conjugate in SERS. Additionally, electrostatic interactions and the attachment of biotin-streptavidin conjugates to nanoparticles (NPs) have also been utilized in the creation of adaptable nanomaterials ( Chauhan et al., 2022).

**Figure 20.**
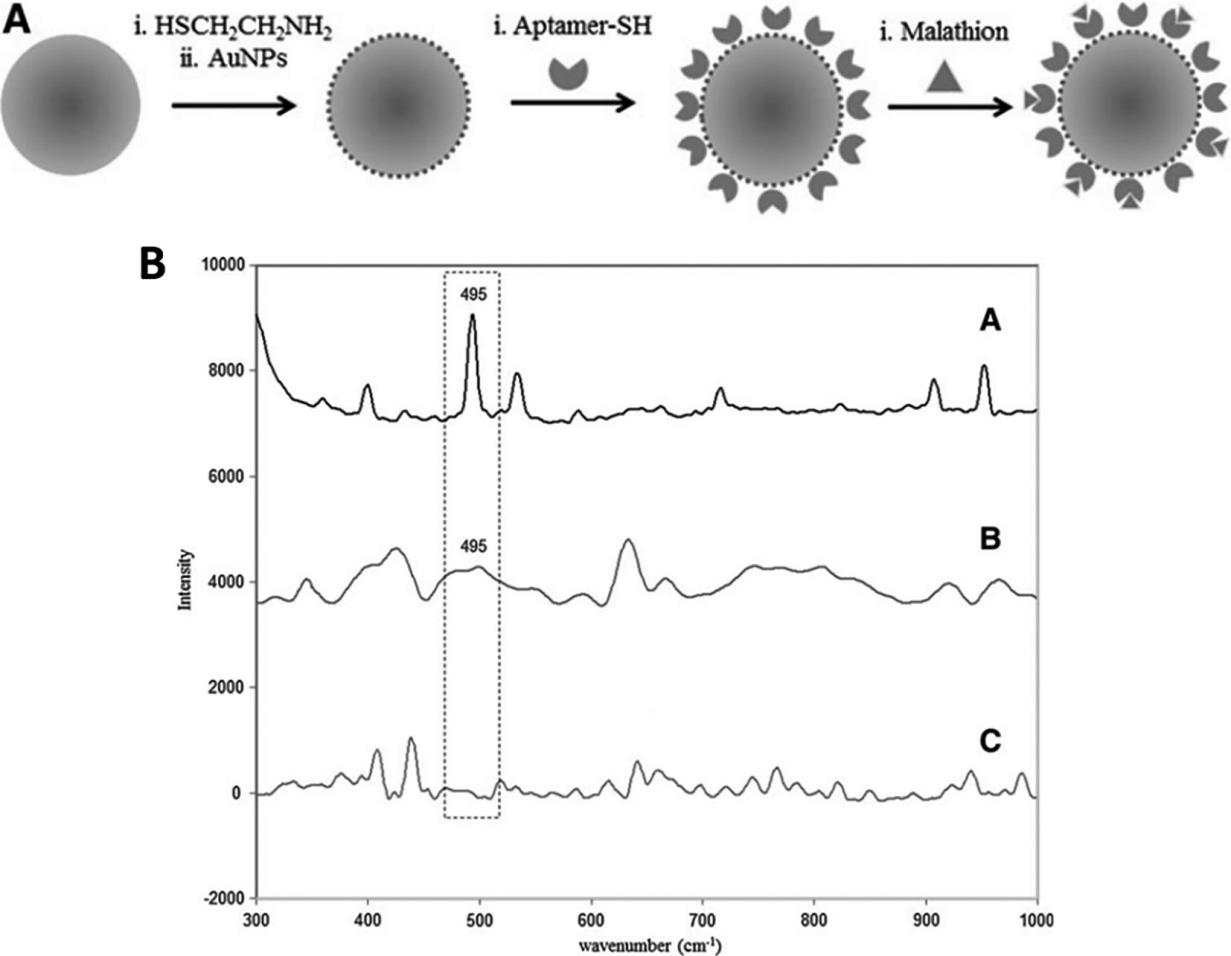
(A) Illustration demonstrating synthesis of polymer-AuNP-aptamer substrates for the SERS based detection of malathion (B) Comparison of SERS detection of malathion at 495 cm ^-1^ peak with polymer-AuNPs aptamer substrates 16.5 μg/mL of malathion (A) 3.3 μg/mL of malathion (B) and blank buffer solution (C) Spectra are offset for clarity. (This figure has been reproduced with permission from ( Barahona et al., 2013), Copyright from (2013) Industrial Biotechnology).

In an interesting approach by Barahona et al. *,* in 2013, an SERS-based aptasensor on micron-sized polymer particles was used for the detection of malathion. Methacrylic acid monomers and ethylene glycol dimethacrylate co-monomers were subjected to precipitation polymerization to synthesize polymer particles in acetonitrile. The use of acetonitrile facilitates a smaller pore size, whereas methacrylic acid supplies carboxyl groups that bind gold. Controlled aggregation of AuNPs resulted from the conjugation of AuNPs with the polymer using 2-aminoethanethiol. Thiolation was used to connect the aptamers to the nanoparticles. The thiol–gold interaction caused the polymer-AuNP-aptamer complex to adhere to the metal surface. The LOD was 33.3 μg/mL, and the concentration ranged from 3.3-33.3 μg/mL ( Barahona et al., 2013). Figure 20 depicts the fabrication of polymer-AuNP-aptamer substrates and the specific detection of 2, 4-DNT. Table X presents some recent studies describing the effect of biological conjugation, their effect on Raman enhancement, and some critical insights for improvement.

**Table X.** List of commonly used biological conjugations in SERS-based sensors.

Sl. No.	Analyte	Method	Bioreceptor	Linker chemistry	Sample	LoD	Remarks	Ref
1	Malathion	SERS-based aptasensing using micro-polymer particles synthesized by precipitation polymerization	Aptamer	Gold & 2-aminoethanethiol linking	-NA-	33.3 ppb	Despite the use of plasmonic nanoparticles and specific bioreceptor, the LoD was relatively lower than expected. Cross-sensitivity testing and Real sample testing was not done.	( Barahona et al., 2013)
2	Cocaine	SERS-based apatmeric sensing of cocaine using model targets	Aptamer		Tap water	303 ppb	Conjugation of substrate with aptamer was based on electrostatic interactions. Hence, use of covalent linker chemistries can detect and improve the repeatability.	( J. Chen et al., 2008)
3	3,3,4,4-tetrachlorobiphenyl (PCB-77)	DNA aptamer based-SERS using Ag nanorod arrays	DNA Aptamer	Silver-thiol linking	-NA-	96.32 ppm	The linear range of detection of the substrate is not specified and substrate repeatability is not conducted.	( K. Sun et al., 2016)
4	Anthrax biomarker	Peptide conjugated gold nanoparticle tagged with DSNB reporter used as SERS substrate	16-aminoacid peptide	-	-NA-	6.1 fM	This paper specifies that peptides are more stable bioreceptors than antibodies.	( Ryu et al., 2010)

## 8. SERS biosensing

A typical SERS-based biosensor involves the use of a robust SERS-active substrate with customized linker chemistry for conjugation with an analyte-specific bioreceptor. Commonly used bioreceptors include antibodies, aptamers, enzymes and polysaccharides. The concentration of the target analyte near the plasmonic field of the SERS-active substrate due to binding with the bioreceptor can enhance Raman scattering and hence improve the detection sensitivity. The use of bioreceptors assists in the differentiation of molecules with similar structures and the detection of specific analytes from complex specimens. However, the use of bioreceptors with a SERS-based system is also associated with challenges, such as degradation of bioreceptors in sensing with environmental or complex samples, high cost of the bioreceptor that limits widespread use, and non-specific interactions of sample matrix components, leading to incorrect results. These challenges may be reduced to a certain extent by optimizing the SERS system parameters, such as the choice of the excitation laser wavelength, laser power, integration time, and scans to average. Typically, the use of longer-wavelength lasers can minimize autofluorescence and background noise from bioreceptors. A high laser power can denature the bioreceptor and reduce background fluorescence, thus requiring the use of a low laser power. A higher integration time and scans to average can enhance background noise, along with the signal intensity, which can be minimized by using appropriate optics, such as optical filters and light polarization optics.

**Figure 21.**
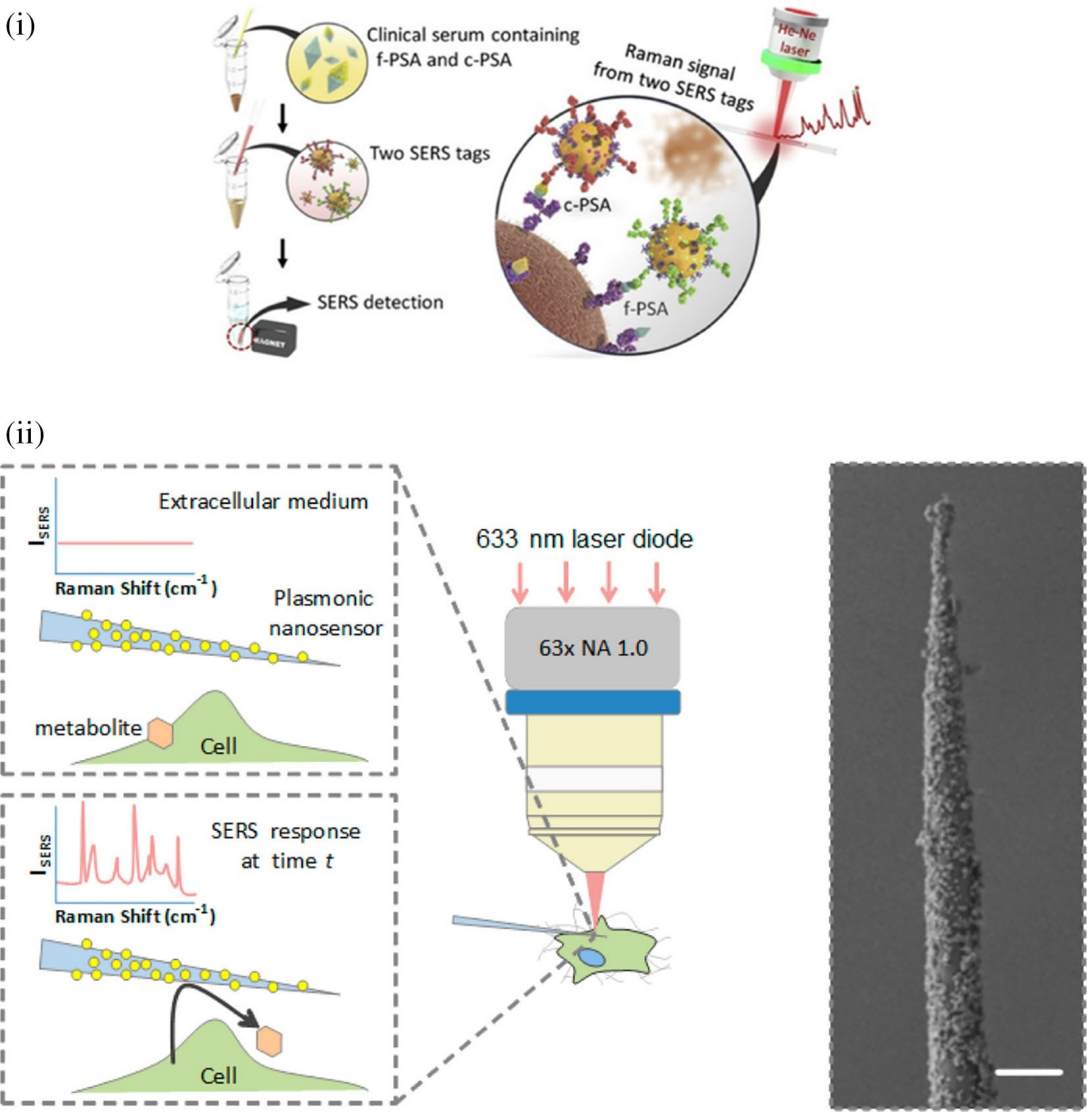
(i) Schematic illustration of dual-reporter SERS immunoassays for the detection of two proteins labelled with dual prostate-specific antigen (PSA) markers for simultaneous detection from the two SERS tags (This figure has been reproduced with permission from ( P. Li et al., 2020), Copyright from (2020) Current opinion in Biomedical Engineering). (ii) Representation of SERS based nanosensor to monitor the cellular secretion. The SERS nanosensor is positioned under the Raman microscope and monitored in real-time detection for the molecules diffused from the MDCKII epithelial cells. (This figure has been reproduced with permission from ( Lussier et al., 2016), Copyright (2016) ACS Nano letters).

Currently, biosensors play a crucial role in the qualitative and quantitative determination of several biomolecules, which helps in monitoring biological processes and disease diagnosis. Compared to other biosensing platforms, Surface-enhanced Raman scattering (SERS) shows a million-fold enhancement in Raman signals when using appropriate SERS substrates. Since the discovery of the SERS phenomenon using a roughened metallic silver surface, it has evolved as a method of interest in bioanalysis across various fields, such as biochemistry, chemistry, material science, and life science. SERS has been established as the most sensitive and powerful spectroscopic technique for biosensing applications ( Zhou et al., 2020), ( Muhammad & Huang, 2021). A recent study by Li. P et al. presented a schematic for the synthesis of dual-reporter SERS probes for the specific detection of prostate-specific antigens, as shown in Figure 21(i) ( P. Li et al., 2020). SERS has several advantages over traditional bioanalysis methods, such as ultrahigh sensitivity; thus, it is useful even in the detection at a single molecular level. It shows less sensitivity towards photobleaching and photodegradation, thereby aiding long-term monitoring. Additionally, SERS makes convenient multiplex detection possible using single-wavelength excitation ( Zong et al., 2018). Breakthroughs in the fields of synthetic chemistry and material engineering have aided in broadening the applications of SERS beyond those in which the molecules bind to metallic nanostructures and are resonant in the visible wavelength region. Capture agents bring low-metal-affinity analytes close to plasmonic surfaces, where large electromagnetic enhancement allows signal amplification, thereby expanding the library of molecules detectable by SERS, which includes molecules of biological and clinical importance ( Cardinal et al., 2017). The two available methodologies for SERS-based applications are label-free detection and indirect approaches that use SERS tags. The goal of SERS-based label-free detection is to obtain vibrational spectroscopic information of molecules through direct interaction between the samples and SERS-based nanostructures, resulting in enhanced intrinsic fingerprint information of biological and biomedical samples. A study by Lussier et al. used a sharp plasmonic nanosensor for cellular piercing to detect the time-dependent secretion of cellular metabolites with a He-Ne laser diode with the same SERS-active substrate, as shown in Figure 21(ii) ( Lussier et al., 2016b). Label-free SERS approaches can provide information on the structure of proteins, nutritional quality of food products, and detection of distinctive pathogens present in clinical samples as well as biological processes occurring at the cellular level ( X.-S. Zheng et al., 2018). Successful implementation of both label-free SERS and indirect SERS in biomedical and biological analyses requires the powerful and rational design of plasmonic nanostructures.

Typically, metallic nanostructures for SERS are made by four strategies: bottom-up, self-organization, and top-down processes; a layer of reporter molecules showing a unique and strong Raman fingerprint that enables indirect detection; an outer layer coating that aids in improving its biocompatibility and stability; and bioconjugations that enable specific detection of the analyte ( Cialla-May et al., 2017). Noble-metal nanoparticles have traditionally been used for the synthesis of SERS-active substrates because of their unique ability to exhibit local plasma resonance. Signal enhancement is dependent on various parameters, including the size, shape, morphology, arrangement, and dielectric environment of the nanoparticles. Owing to these reasons and their enormous advantages, studies have now focused on tunable nanofabrication and the synthesis of noble metallic nanoparticles. Several reports have been published on the development of metallic substrates, focusing on their various morphologies and applications in biosensing, such as nanospheres, nanocubes, aggregates, and well-designed one-dimensional (1D), two-dimensional (2D), and three-dimensional (3D) arrays and patterns. These special structures are of great use in the trace detection of chemical and biological molecules such as food additives, antibiotics, and disease markers. Although several substrates and designs have been developed, the production of highly sensitive, selective, stable, and reproducible substrates using a simple, robust, low-cost, and high-yield method remains a challenge ( Cao et al., 2013). Point-of-care (POC) SERS analysis is an emerging technique in which SERS measurements are recorded on-site, in contrast to conventional laboratory testing methods that use benchtop SERS instruments. Most point-of-care testing methods are based on lateral flow immune assay (LFIA), which frequently has low sensitivity. SERS with significant signal enhancement via the creation of “hot spots” have enormous potential to address the sensitivity limitation, with POC SERS garnering the most attention. Several flexible materials have been utilized as SERS substrates for SERS-based POC diagnostics, including paper, flexible polymers, graphene, and nanowires, each contributing to different applications to the substrate by virtue of their characteristics. These materials enable the mass synthesis of SERS substrates that are affordable, disposable, and scalable. To maintain the high specificity of the SERS technique, the molecular functional interfaces of all these flexible substrates must be carefully considered to interact precisely with the analytes ( Perumal et al., 2021).

## 9. Some critical design aspects for SERS Biosensors

An important consideration in designing a SERS biosensor is the elimination of additional fluorescence from the substrate, linker, or bioreceptor. Fluorescence is defined as a type of luminescence that occurs only when a substance is irradiated with electromagnetic radiation ( “IUPAC-Fluorescence,” 2014). The fluorescence of the substrate was nullified by the use of a quencher. For example, graphene quantum dots (GQDs) are liable to fluoresce owing to quantum confinement. Mn _3_O _4_ (manganese (II)/ (III) oxide) was used for fluorescence quenching of the GQDs and suppression of the Raman spectral fluorescence background. This property of GQD-Mn3O4 nanocomposites has a high potential for the differentiation of cancer cells and normal cells by SERS ( Z. Zhao et al., 2014). The conjugation chemistry of biotin-streptavidin forms a heterostructure with fluorescence properties and assists the surface plasmonic properties of SERS sensors ( X. Qin et al., 2019). Studies on graphene indicate that resonance Raman spectroscopy of the substrate can potentially inhibit fluorescent molecules and can be applied to the low-concentration detection of fluorescent molecules. A study by Xie et al. indicated that the fluorescence quenching of R6G adsorbed on graphene was of the order of 10 ^3^ ( L. Xie et al., 2009).

In addition to fluorescence, reproducibility of the substrate design is an important consideration for the development of SERS biosensor. Reproducibility was defined as the closeness between individual test results obtained with same material under different testing conditions ( “IUPAC- Reproducbility,” 2014). The synthesis of hybrid nanoparticles, that is, the modification of colloidal metal nanoparticles with additional nanostructures, was observed to improve the SERS performance. The use of graphene-modified surfaces has been observed to improve the stability and repeatability of SERS signals, such as the repeatable determination of 6-mercaptopurine (6-MP) in tablets using graphene-modified metal nanoparticles ( W. L. Fu et al., 2014). Other studies have suggested the use of a flow system for sample mixing with colloidal substrates to optimize nanoparticle aggregation for SERS-based detection. A sensor designed by integration of a lab-on-chip setup with a microfluidic system for quantification of mitoxantrone showed a good concentration range of 2.5 × 10 ^−9^M to 1 × 10 ^−6^M for plasma and serum. Despite achieving good repeatability by integrating the flow system into the substrate surface, the sensor requires a complex and expensive setup ( McLaughlin et al., 2002). Surface modification of substrates by chemical functionalization of metal surfaces allows for improved repeatability. Klarite substrates have been used in a recent study by Litti et al. al., displayed good repeatability for the detection of specific anticancer drugs. This work also indicates that hot spot density is crucial for determining the limit of quantification, which is related to sensor repeatability ( Litti et al., 2016).

A higher signal-to-noise ratio is a crucial consideration for designing a SERS biosensor, as high-quality spectral data acquisition is achieved using holographic notch filters, which can reject the Rayleigh scatter of the analyte molecule ( McCreery, 2000). In the case of long-range penetration of the excitation wavelength for biomedical applications of SERS, defined spectral windows are observed in the NIR region ( A. M. Smith et al., 2009) based on the cellular composition of the body. Additional fluorescence is observed due to the relatively higher tissue auto-fluorescence, which contributes to the broad background of the Raman signal, thus reducing the SNR. Optimization of the SNR for SERS spectral recording is essential for achieving rapid results and a lower sensitivity of the sensor ( McCreery, 2000). Owing to the intrinsically weak nature of Raman spectroscopy, one part of the fluorescence is seen for 6-10 orders of magnitude of Raman scattering per cross-sectional area ( Etchegoin, 2009). Therefore, to ensure a sensitive and analyte-specific Raman signal with minimum background noise, the measured Raman spectra should be free of trace impurities. Hence, the best SNR can be achieved with a minimal background signal ( McCreery, 2000). Additional limitations with real-world applicability of SERS-based biosensors includes the complexity of sample matrix that affects the analyte Raman signal and its sensitivity ( Terry et al., 2022), the substrate shelf-life based on their susceptibility for oxidation or degradation, sensitivity to environmental conditions such as temperature and light that significantly affects bioreceptor performance ( Pérez-Jiménez et al., 2020), imprecise quantification due to variability in the signal amplification and unreliable calibrations ( Terry et al., 2022), integration with standard techniques for enhanced analytical performance, and real-time monitoring ( Pilot et al., 2019). In summary, though SERS-based sensing offers exceptional sensitivity and specificity, addressing these challenges is crucial for expanding the practical utility of SERS-based sensing systems.

## 10. Disposable SERS systems for specific analyte detection

Typically, disposable SERS systems employ one-time-use substrates that mitigate the need for multiple cleaning procedures and the risk of cross-contamination. They are more beneficial than standard substrates owing to their low cost, versatile detection, robustness, and user-friendliness. These disposable SERS systems can be tailored for specific analyte sensing with improved sensitivity and selectivity through specific surface functionalization or the use of a specific bioreceptor to capture the analyte of interest. The compact design and ease of use of the system can facilitate on-site use and measurement of real-time data.

### 10.1 Small molecules

Small molecules are organic compounds with molecular weights lower than 1000 Da that are fundamental for chemical and biological processes. Common small molecules include antibiotics, pesticides, chemicals, and heavy metal ions. The detection of small molecules in food and water sources is crucial for preventing detrimental effects on the environment and human and animal health. Early detection of these small molecules can assist in formulating the necessary regulatory measures to devise strategies for the prevention of adverse health disorders.

***10.1.1 Pesticides and chemicals***

The indiscriminate use of pesticides poses a serious threat to consumer health and can possibly lead to soil contamination and, eventually, contamination of water bodies. The chronic and fatal effects on human health are proportional to pesticide exposure time, pesticide concentration due to bioaccumulation, and chemical characteristics of the pesticide( L. Zhang et al., 2014). Thus, the detection of pesticides in food, soil or water bodies is essential to prevent their entry into humans. Conventional strategies, including HPLC and GC-MS, are expensive and require longer time for result acquisition. Other strategies include colorimetric detection, RI sensitivity detection, and Raman spectrum-based detection, which may not detect trace levels of pesticides due to their lower sensitivity, although these standard techniques cannot provide a quantitative estimation of the analyte.

**Figure 22.**
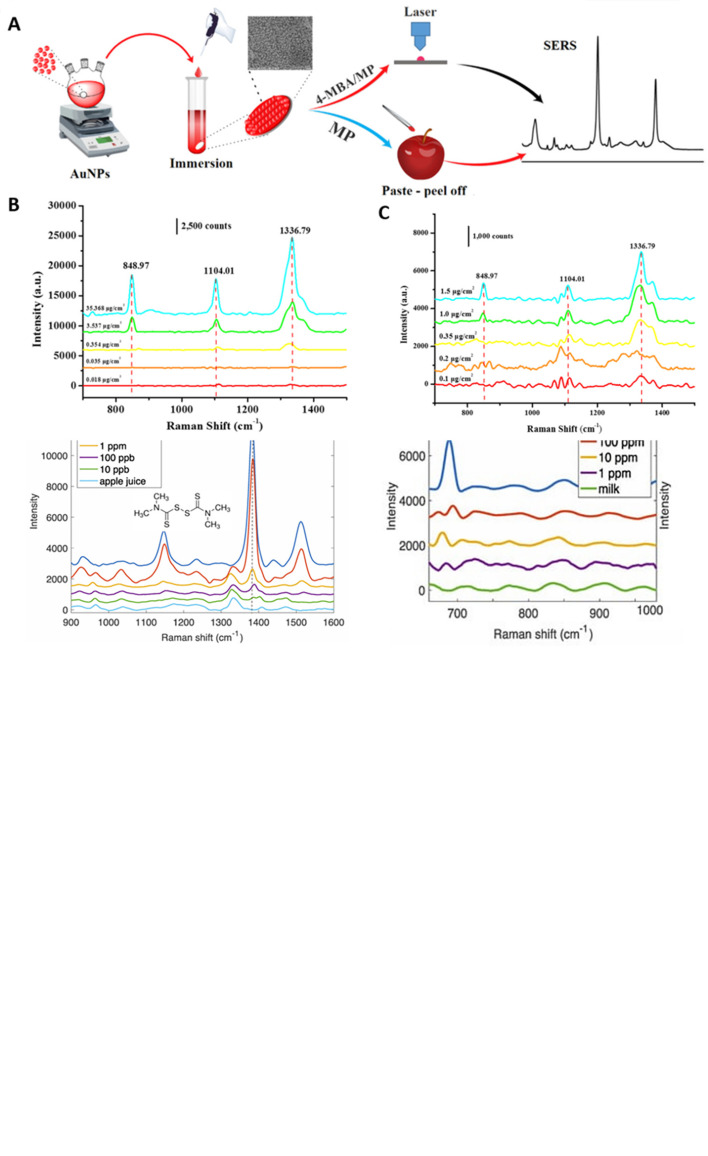
Disposable SERS system for specific detection of pesticides and chemicals (i) (a) Preparation of the SERS substrate for the detection of food contaminants (thiram and melamine from apple juice and milk respectively), (d, e) SEM images of the dendritic silver nanostructures, (g-f) SERS spectra of thiram (peak at 1384 cm ^-1^) and melamine (peak at 695 cm ^-1^) at varying concentrations, in unprocessed apple juice and milk samples (This figure has been reproduced with permission from ( Dies et al., 2018), Copyright from (2018) Sensors), (ii) (A) Schematic of the paper-based SERS substrates for the detection of methyl parathion on the fruit peels surface, (B) SERS spectra of methyl parathion (MP) (concentration range-0.018 μg/cm ^2^ to 35.368 μg/cm ^2^) with a paper-based substrate, and (C) SERS spectra of MP on the surface of apple using the paper-based substrate (This figure has been reproduced with permission from ( J. Xie et al., 2020), Copyright from (2020) Spectrochimica Acta Part A: Molecular and Biomolecular Spectroscopy).

In an interesting study by Dies et al. in 2018, the presence of thiram, a fungicide used in agriculture, was checked in apple juice by SERS using dendritic silver nanosubstrate on microelectrodes by maskless photolithography. It recorded an LoD of 115ppb and a liner detection range of 0.01-100 ppb, on the microelectrode chip by using a preconcentrated nanoparticle solution, as shown in Figure 22(i). The key peak at 686–703 cm ^−1^ was used for identification and quantification ( Dies et al., 2018). However, the use of microelectrodes as the substrate with a high-energy laser can ablate the electrode surface, resulting in the generation of surface interferents that significantly affect the sensitivity of the sensor. Another interesting approach by Xie et al. (2020) used bipyramidal AuNPs for trace detection of methyl parathion in apple peels. The senor showed an LoD lower than other substrates, i.e., 98.63 ng/cm ^2^, 31.56 ng/cm ^2^ and 36.58 ng/cm ^2^ on peels of cucumber, tomatoes and apples respectively ( J. Xie et al., 2020). The “press -peel off” method of detection is shown in Figure 22(ii) is user-friendly, but the recovery rate is not significant and therefore cannot aid in field deployment. Despite the use of a 785 nm laser and spherical AuNPs, a considerable detection limit with a 10-fold EF was achieved. Table XI presents the specific detection of pesticides and chemicals using disposable substrate-based SERS systems.

**Table XI.** Disposable substrates for SERS-based detection of pesticides and chemicals.

Sl. No.	Analyte	Substrate	Method	Sample	LoD	Remarks	Ref
1	Melamine and Urea	Paper	In-situ grown silver nanostructures on filter paper used as SERS substrate	Milk	213.14 and 28.8 ppth	Better sensitivity of the developed sensor can be obtained as the substrate comprises of anisotropic nanostructures. Filter papers with varying porosity can be analyzed as the substrate is synthesized by *in-situ * method and can result in better periodicity, thus detecting lower concentrations.	( Verma et al., 2021)
2	Thiram	PDMS	Flexible SERS substrate synthesized using Ag nanorod embedding into PDMS film	Apple peel	Mass-to-area ratio was 2.4 × 10 ^−9^ g/cm ^2^	The Raman spectrum intensity of the developed sensor significantly varied with the standard Raman reporter and thiram sample. However, the LoD of this sensor was identified to be much lower than the MRL of thiram, hence can be an effective solution for POC-based pesticide detection.	( Kumar et al., 2017)
3	Thiram	Bacterial nanocellulose	Bacterial cellulose and gold nanoparticle-based SERS-substrate for paste and peel testing of thiram	Apple peels	0.98 ppm	The sensor could achieve better sensitivity by using the anisotropic nanoparticles in the substrate. Though the substrate achieved considerable enhancement in the SERS signal, lower LoD is possible by the *in-situ * reduction method of the substrate.	( Xiao et al., 2023)
4	Methyl parathion	PDMS	Gold nanostar-embedded PDMS film used as SERS substrate	Apple peel	1.946 ppth	The PDMS layer thickness optimization was not performed, which can mitigate the plasmonic field of the nanostructures thereby resulting in lower sensitivity. This method of pesticide detection on the surface of food samples is of great use as POC as it eliminates the need for preprocessing.	( X. Ma et al., 2022)
5	R6G and Melamine	Plastic film of polyethylene-terephthalate	Screen printed Ag nanoparticles on polyethylene-tere phthalate used as SERS substrate	Milk	47.9 ppt & 12.6 ppm	Sensitivity of the sensor is significantly lower while tested with a real sample. This method of substrate synthesis can overcome uneven radial distribution of samples.	( W. Wu et al., 2015)

***10.1.2 Antibiotics***

Excessive use of antibiotics leads to antibiotic resistance in patients, rendering the drug ineffective. Commonly used antibiotics include tetracyclines, beta-lactams, fluoroquinolones, amphenicols, and carbapenems. Traces of these antibiotics may be found in food and water sources because of their excessive use in animal treatments that remain as residues or by the use of contaminated water for agricultural practices. Currently, SERS detection methods are used extensively to detect trace levels of antibiotics in food and water sources.

**Figure 23.**
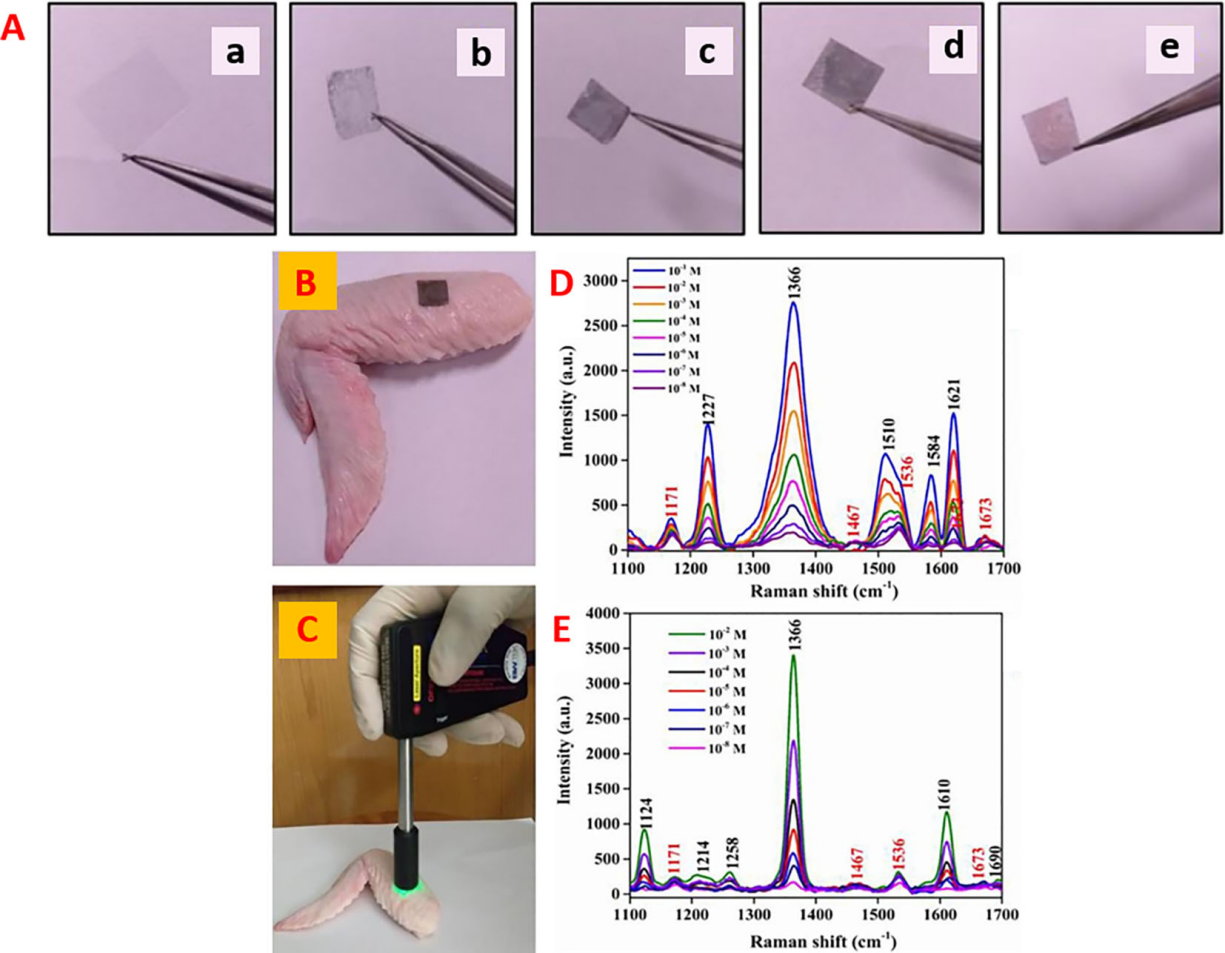
(A) Photographs of (a) pure PMMA film, (b) Au/PMMA-1 film, (c) A/PMMA-2 film, (d) Au/PMMA=3 film, (e) Au/PMMA-4 film; (B, D) D Photographs of the SERS-based detection of (B) Ciprofloxacin and (C) Chloramphenicol on chicken wings with flexible Au-NSs/PMMA SERS substrate using 532 nm laser and (D, E) their corresponding SERS spectra (This figure has been reproduced with permission from ( Riswana Barveen et al., 2022), Copyright (2022) Chemical Engineering Journal).

An interesting study was conducted by Shi et al. (2017) for the detection of neomycin in milk using lateral flow immunoassay (LFA)-based SERS. This testing strategy displayed good sensitivity with an LOD of 0.216 ppb and a recovery rate between 89.7%- 105.6% ( Shi et al., 2017). Although exceptional sensitivity was observed, the sensor lacked coherence of the laser excitation wavelength mismatch and absorbance of the nanoparticles. The use of monoclonal antibodies could not ensure specificity, as better cross-reactivity with other antibiotics such as enoxacin was observed. In another interesting study by Pinheiro et al. in 2018, gold nanostars (GNS) decorated on magnetite nanoparticles (MNP) were used for the trace detection of tetracycline. This method estimated LOD of 44.4ppb and 444ppm in ultrapure water and mixed aqueous solutions respectively ( Pinheiro et al., 2018). However, this substrate sensitivity is primarily dependent on the adsorption of tetracycline, which may significantly vary with Au branches and surface irregularities on MNPs that generate hotspots. Instead, the use of a specific bioreceptor may improve the adsorption capacity and specificity of the substrate. pH studies for tetracycline adsorption showed that pH 5-6 was the most favorable for adsorption; however, pH regulation in complex sample matrices may not be possible during rapid analysis. In a recent study by Riswana et al., gold nanostars (AuNSs) were decorated on a PMMA substrate for SERS-based detection of ciprofloxacin and chloramphenicol in chicken wing samples, as shown in Figure 23. The developed system achieved an LoD of 3.41 × 10 ^−11^ and an EF of 2.03 × 10 ^9^ ( Riswana Barveen et al., 2022). The use of a novel AuNS synthesis method utilizing a UV-C light-based photoreduction process is simple, yet effective. The substrate showed exceptional sensitivity and multiplexing ability; however, no cross-sensitivity testing was conducted. Table XII discusses the disposable substrates for the SERS-based detection of antibiotics.

**Table XII.** Disposable substrates for SERS-based detection of antibiotics.

Sl.No.	Analyte	Substrate	Method	Sample	LoD	Remarks	Ref
1	Tetracycline	Cardboard	SERS substrate from cardboard-based layer coated with evaporated aluminum	Milk	0.01 ppm	SNR of the Raman spectrum of tetracycline is not significantly higher than the reference. The absorption spectrum of AgNPs was not specified and the SEM image shows isotropic NPs which cannot have absorbance at 600-650nm.	( Marques et al., 2019)
2	Tetracycline	Filter paper	Ag nanodisks coated filter paper-based SERS substrate	-	444.4 ppt	The linear detection range of the sensor was not mentioned. Cross-sensitivity and real sample testing were not performed using the developed sensor as it can significantly vary with the obtained LoD.	( Pagano et al., 2021)
3	Ciprofloxacin and chloramphenicol	PMMA films	Flexible SERS substrate using AuNS/PMMA film.	Chicken wing samples	0.11 ppb	The use of PMMA as substrate is advantageous due to its high optical transparency. AuNS incorporation in the substrate improved the Raman signals (EF=2.3*10 ^9^).	( Riswana Barveen et al., 2022)
4.	Sildenafil	Teflon membrane	Hydrophobic slippery surface (Teflon membrane) coated with concentrated plasmonic gold nanoparticles	Healthcare products	0.076 ppm	Low sensitivity of the sensor is due to a mismatch of the absorbance range of the plasmonic nanostructure and the excitation laser. Due to surface hydrophobicity, the sample restriction may be crucial than the hotspot generation, resulting in lower LoD.	( D. Zhang, You, et al., 2019b).

***10.1.3 Heavy metals & other small molecules***

**Figure 24.**
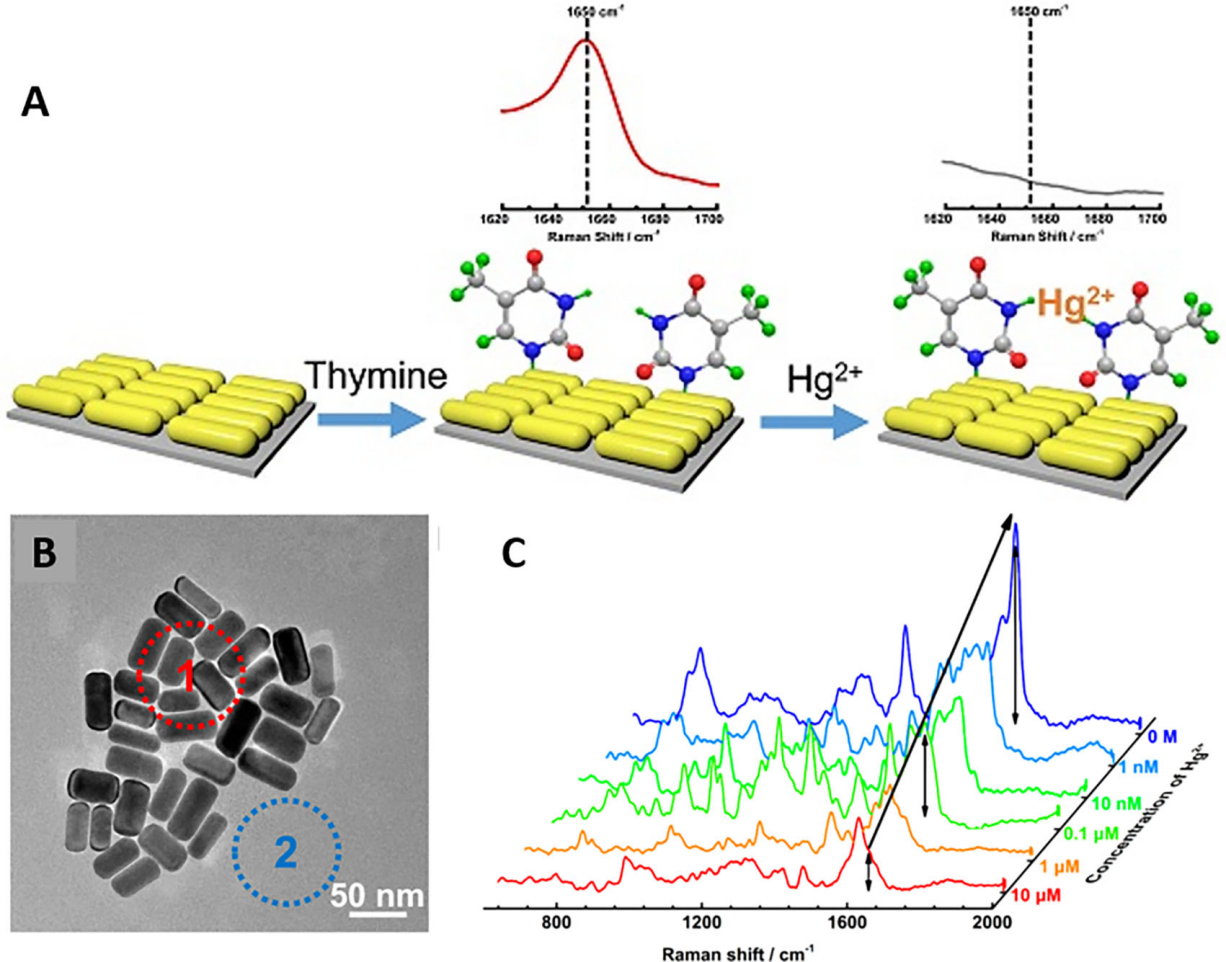
(A) Schematic illustration of the SERS based detection of Hg ^2+^ ion on the Au NRs@Thymine (This figure has been reproduced with permission from ( H. Yang et al., 2017), Copyright from (2017) Nanomaterials). (B) TEM image of the Au NRs@T adsorbed with Hg ^2+^ ions, (C) SERS spectra of the Au NRs@T with varying Hg ^2+^ ion concentrations.

Contamination of food and water by heavy metal ions such as mercury (Hg ^2+^), lead (Pb ^2+^), arsenic (As ^2+^), and cadmium (Cd ^2+^) can potentially affect the physiological functions of the nervous system, as well as injure vital organs such as the kidneys and liver. Accidental ingestion of food containing these contaminants increases the risk of heart disease, neurological damage, such as tremors, and impairment of cognitive function. The most toxic heavy metal ion in water sources that affects the environment and human health is mercury (Hg ^2+^), and its accumulation in humans leads to neurological disorders. A disposable SERS substrate developed by Yang et al., ( H. Yang et al., 2017), as shown in Figure 24, by the deposition of thymine for simultaneous detection of Hg ^2+^ and Pb ^2+^ ions in drinking water employing oligonucleotide functionalized gold coated polystyrene microspheres (PSMPs). The LoD of the fabricated substrate was found to be 0.1ppb for Hg ^2+^ ions, which is far lower than the limit prescribed by the World Health Organization (WHO) ( Guo et al., 2023). Despite the excellent sensitivity and ability to specifically detect Hg ^2+^ from untreated river samples, cross-sensitivity analysis was not conducted. Table XIII discusses the disposable substrates for the SERS-based detection of heavy metals and other small molecules.

**Table XIII.** Disposable substrates for SERS-based detection of heavy metals and other small molecules.

Sl.No.	Analyte	Method	(Bio) Receptor	Linker chemistry	Sample	LOD	Remarks	Ref.
1	Rhodamine 6G	Disposable paper-based hydrophobic substrate coated with aggregates of colloidal gold nanoparticles	-NA-	-NA- (Gold nanoparticles were drop casted on the substrate)	Cytochrome C	2.06 ppb	Selectivity of AuNP loaded substrate was not checked with other molecules. Absorption peak range of AuNP and the range of the SERS excitation laser do not correlate with each other.	( Geng et al., 2020)
2	Nickel (Ni ^2+^), Cadmium (Cd ^2+^), Copper (Cu ^2+)^	Paper chromatography SERS (PC-SERS) by ion-sputtering gold on filter paper	-NA-	Gold & thiol interaction of AuNP and 4-mercaptobenzoic acid	Rice	~58 pbb	Sensitivity of the sensor can vary by the chelating ability of the 4-MBA with the heavy metals, thus affecting the repeatability. Selectivity of the sensor was not tested which can account for cross-sensitivity.	( Yuqi Song et al., 2020b)
3	Cobalt chloride (CoCl _2_), Lead chloride (PbCl _2_)	Photolithography of polymer films on the glass substrate and covalent interaction by gold-Argon sputtering.	Diethylenetriaminepentaacetic acid (DTPA)	Aldehyde (-CHO) & amine (-NH _2_) interaction	-NA-	0.13 ppb & 0.18 ppb respectively	Sensitivity and SERS EF were suggested to be affected by the analyte’s ionic radius and valence electrons. As the detection strategy works by metal ion chelation, the selectivity of the sensor will be affected.	( Guselnikova et al., 2017)
4	Bisphenol-A	SERS-LFA on nitrocellulose membrane using Gold nanostars	Anti-BPA antibody	EDC-NHS	-NA-	0.073 ppb	Higher plasmonic activity of the GNSs by 1064 nm excitation laser cannot be correlated with the absorbance spectrum. No real sample testing was performed which might have a load of structurally analogous molecules.	( L.-K. Lin & Stanciu, 2018)

### 10.2 Nucleic acids

**Figure 25.**
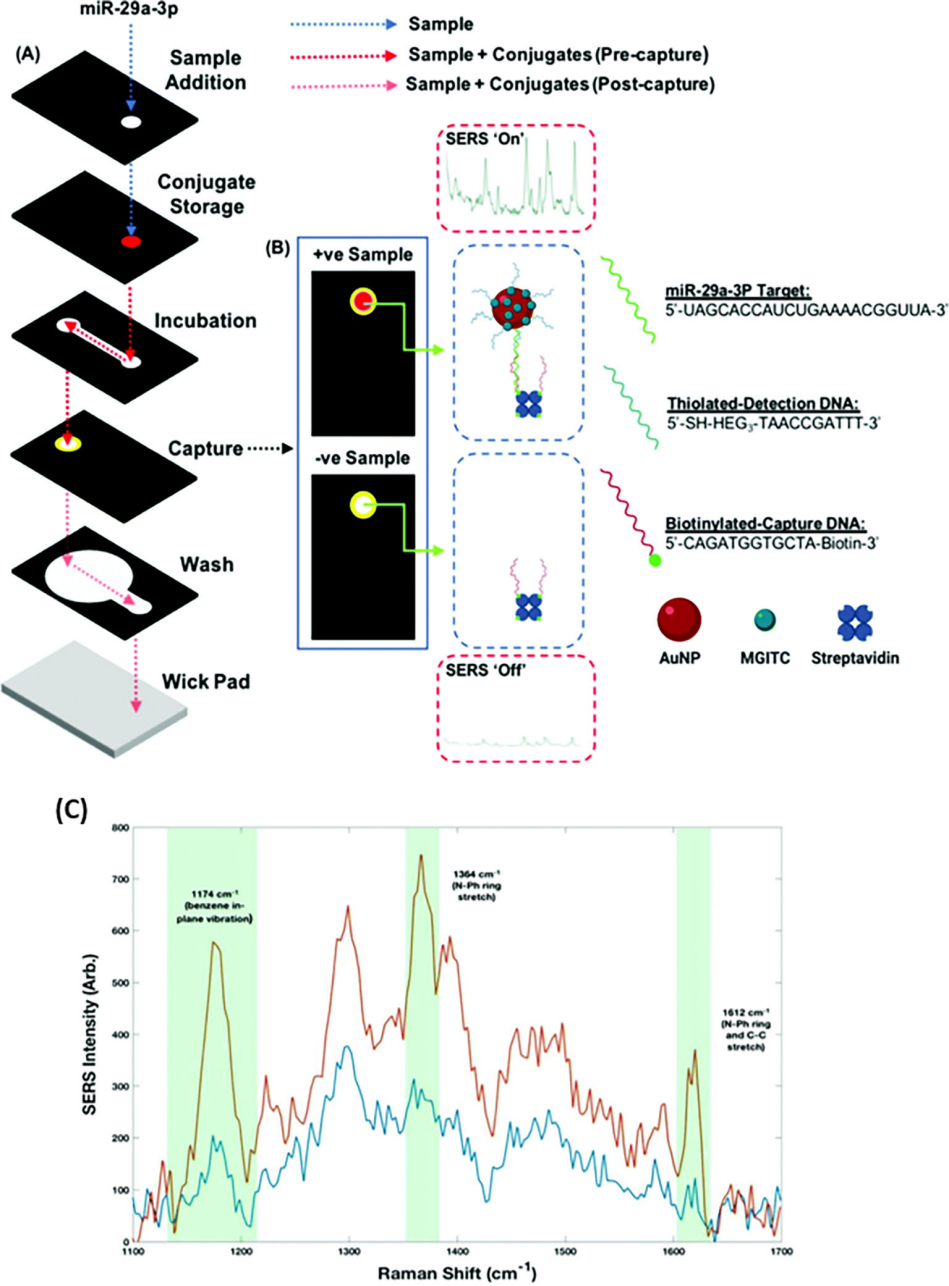
(A) Detailed schematic of the 3D paper-based microfluidic device for the detection method of miR-29a in a 3D paper-based microfluidic device; (B) Sample readout images with both colorimetric and SERS for samples with (+ve) and without miR-29a (−ve). and (C) SERS spectra representing the 18 pg μL ^−1^ (blue) and 360 pg μL ^−1^ (red) concentrations of miR-29a, highlighting three signature methyl green isothiocyanate (MGITC) peaks. (This figure has been reproduced with permission from ( Mabbott et al., 2020), Copyright from (2019) Analyst).

Nucleic acids, DNA, and RNA are genetic materials of all living beings that may be utilized as biomarkers. Standard detection methods include southern/northern blotting, reverse transcriptase-polymerase chain reaction (RT-PCR), and microarray-based methods. These methods cannot be used for POU applications because of the need for sample preprocessing, longer assay time/incubation time, high cost, and low sensitivity. Optical and electrochemical detection methods can overcome these limitations, thereby increasing the possibility of on-site nucleic acid detection using SERS-based systems. Recently, Mabbott et al. developed a 3D paper-based microfluidic platform, as shown in Figure 25. The fabricated substrate used malachite green XXX isothiocyanate (MGITC)-functionalized AuNPs coated on Whatman 4 chromatography paper for the specific detection of miR-29a in PBS running buffer. This system showed an LoD of 47 ppm using a handheld Raman spectrometer, thus proving its potential as a POU device ( Mabbott et al., 2020). Despite the POU applications, the authors used a CBEX handheld Raman spectrometer, which is highly expensive for integration into a POU-SERS system. Table XIV discusses disposable substrates for the SERS-based detection of nucleic acids.

**Table XIV.** Disposable substrates for SERS-based detection of nucleic acids.

Sl No.	Analyte	Method	(Bio) Receptor	Linker chemistry	Sample	LOD	Remarks	Ref.
1	Two target DNAs, associated with Kaposi’s sarcoma (KS) and bacillary angiomatosis (BA).	LFA-SERS for dual detection of DNA markers on filter paper via bioconjugation of AuNPs	Kaposi’s sarcoma-associated herpesvirus (KSHV) DNA and bacillary angiomatosis (BA) DNA	Streptavidin-biotin linking	-NA-	0.043 pM & 0.074 pM respectively	Sensitivity can be improved by using nanoparticles with absorption in the laser excitation range. The sensor can be identified with poor cross-sensitivity and specificity when tested with other structurally similar molecules.	( X. Wang et al., 2017)
2	Plasmodium falciparum malarial parasite RNA	SERS of glass capillary sticks by the concept of “Lab in stick.” The sticks are coated with SERS-encoded cube nanorattles functionalized with DNA reporter probes	Complementary ssRNA of the target RNA	Amine (-NH2) to thiol (-SH) cross linker	Blood lysate	2 aM	Reproducibility of this sensor substrate is crucial as the facet of the cube contributes to the absorption and raman signal due to presence of raman reporter.	( Ngo et al., 2018)
3	Mutated KRAS DNA in cancer cells	Microfluidic chip integrated with glass-based SERS substrate synthesized using Au@Ag nanorods	Molecular beacon probes	Electrostatic interaction of CTAB with phosphate groups of ssDNA	-NA-	50 fM	Stability of the bioreceptor conformation is important for specific analyte binding which can be affected by real sample testing.	( L. Wu et al., 2019a)
4	Influenza DNA	Nitrocellulose membrane coated core-shell nanotags of Ag ^MB^@ AuNPs as SERS substrate	ssDNA complementary to Influenza DNA	Aldehyde (-CHO)- Amine (-NH2) linker	Throat swab samples	0.031 pM	Multiplex detection on the NC membrane can affect the selectivity of the sensor due to the presence of a few conserved sequences.	( D. Zhang, Huang, et al., 2019a)
5	microRNA-21	Nitrocellulose membrane coated with Au@4-MBA@Ag NP and	DNA hairpin	Streptavidin-biotin linker chemistry	Diluted human serum samples	84 fM	SERS spectrum of the 4-MBA showed a linear range up to 0.1 pM, while miRNA-21 the lowest range was 0.1 nM. The bioreceptor showed excellent specificity as specified in the cross-sensitivity testing.	( W. Wang et al., 2021)

### 10.3 Proteins

**Figure 26.**
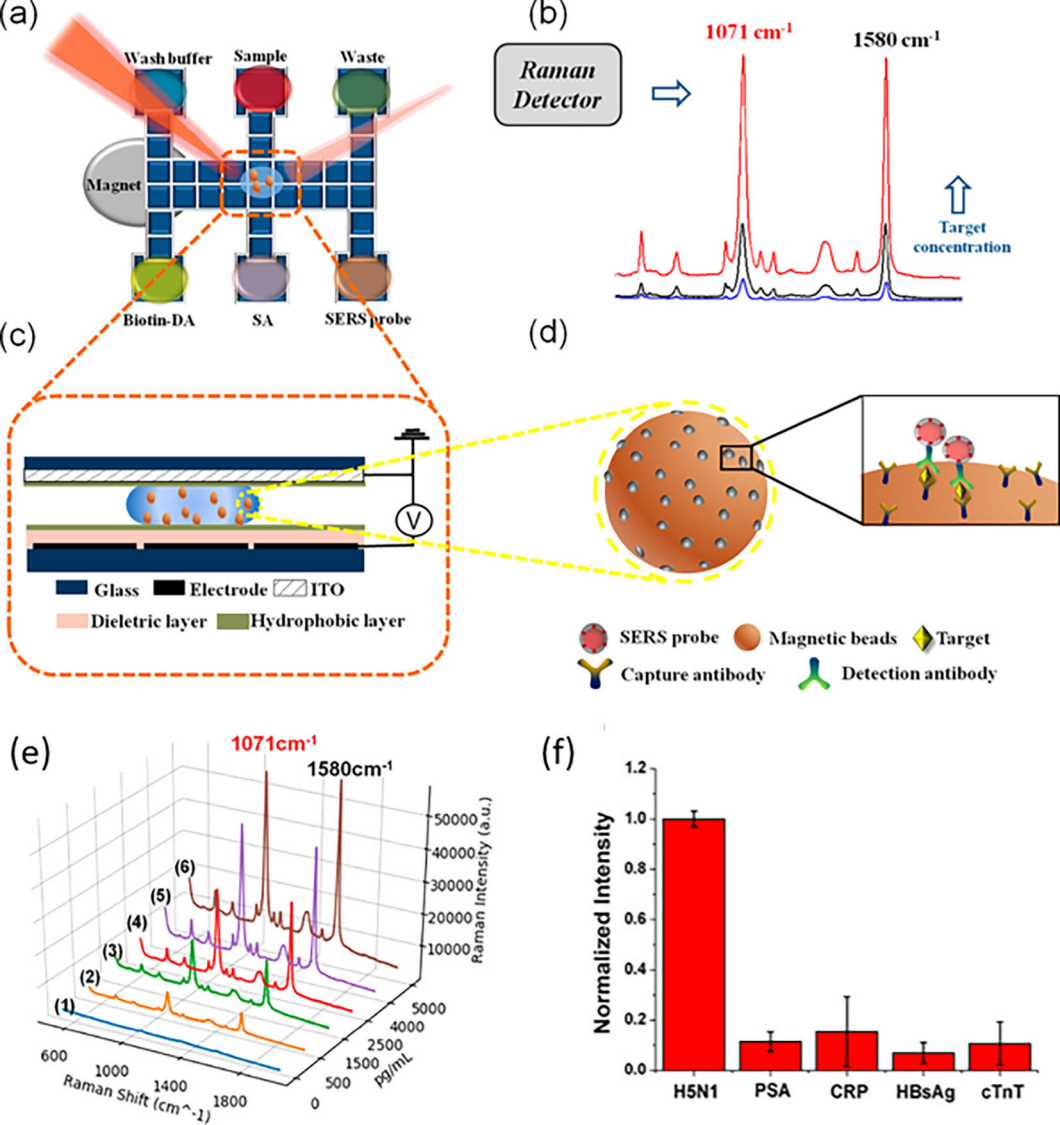
(a) Schematic illustration of DMF-SERS-based immunoassay and bottom side of the DMF chip, (b) Characteristic peaks of 4-MBA on the DMF-SERS chip, (c) DMF chip (side-view) with a droplet on the magnetic beads, (d) Immunocomplex functionalized with SERs tags on magnetic beads, (e) SERS spectra of increasing concentrations (0-5000 pg/mL) of H _5_N _1_ sample, and (f) Cross-sensitivity of the DMF-SERS method (H5N1, 0.085 nM; PSA, CRP, HbsAg, 0.85 nM; cTnT, 0.135 nM) (This figure has been reproduced with permission from ( Y. Wang, Ruan, et al., 2018b), Copyright from (2018) Analytical Chemistry).

Proteins are biomolecules that are synthesized through an *in-vivo* process called translation from genetic material, and may be used as biomarkers or indicators in disease diagnostics as efficient target analytes for SERS-based detection. A strategy was developed by Wang et al. by integrating digital microfluidics with a SERS-based immunoassay for the detection of hemagglutinin of H5N1 influenza virus, as shown in Figure 26. The surface protein was detected in both buffer and human serum samples, and the system showed an LoD of 74 ppb but required less assay time than conventional ELISA and a lower sample volume requirement. The characteristic Raman peaks of 4-MBA were observed at 1071 cm ^−1^ and 1580 cm ^–1^ ( Y. Wang, Ruan, et al., 2018c). Another study by Pinzaru et al. used hydroxylamine-reduced AgNPs to detect lipophilic marine biotoxins, namely okadaic acid (OA), Dinophysistoxin-1 (DTX-1), and Dinophysistoxin-2 (DTX-2) in shellfish tissue. A prominent SERS peak at 1017cm ^−1^ was observed for DTX-1 and DTX-2, which was not observed in the OA fingerprint. OA showed LOD of 200ppm, 71.8 ppm for DTX-1 and 32.6 ppm for DTX-1 respectively ( Pinzaru et al., 2018). The developed substrate showed good sensitivity; however, the excitation laser wavelength and absorbance range of the nanostructures were not coherent. Rather, the use of AuNPs with a typical spectral absorbance at 530 nm may improve sensitivity. Table XV illustrates some of the disposable substrates for the SERS-based detection of proteins.

**Table XV.** Disposable substrates for SERS-based detection of proteins.

Sl. No.	Analyte	Method	(Bio) Receptor	Linker chemistry	Sample	LOD	Remarks	Ref.
1	Human chorionic gonadotropin (hCG) hormone	SERS substrate was developed using AuNP (50nm)/AuNP (17nm) and further drop casted on the conjugate pad of the LFA strip.	hCG antibody	Electrostatic interaction of antibody with the SERS nanotags		1.6 mIU/mL	The absorption range of the nanoparticles do not match the range of the excitation laser, thus affecting the sensor sensitivity. The selectivity of sensor can be affected by the interfering molecules in a real sample, thus affecting the cross-sensitivity.	( Tran et al., 2019)
2	Microcystin-LR (MC-LR) toxin	Paper-based SERS substrate developed n A4 paper by functionalization of gold coated magnetic nanoparticles	MC-LR antibody	EDC-NHS linker chemistry	Skimmed milk	9.9 ppm	The use of antibody for specific detection on a paper-based substrate can result in loss of the respective bioreceptor. Besides, the use of anisotropic nanostructures can improve the sensitivity of the biosensor.	( Hassanain et al., 2017)
3	Melamine & Sudan I	Superhydrophobic-oleophobic (SHP-OP) 3D Ag nanowire mesh-like SERS substrate developed on cleaned glass	-NA-	-NA-	Milk	0.1 ppt	The analyte concentrating method on Superhydrophobic-Oleophobic surfaces is novel and can achieve lower detection limits. The cross-sensitivity studies of the sensor were not conducted, which holds significance due to the lack of bioreceptors.	( X. Li et al., 2014)
4	C-reactive protein (CRP) & Serum amyloid A (SAA)	Fe _3_O _4_@Au SERS tags-based lateral flow assay developed on nitrocellulose membrane by conjugation of	Anti-CRP antibody & Ant- SAA antibody	EDC-NHS linker chemistry	-NA-	0.01 ppb and 0.1 ppb	The selectivity of the sensor can be affected by the presence of interfering molecules while testing a real sample. As the antibodies were sprayed on the test and control line, the sensor reproducibility cannot be ensured.	( X. Liu et al., 2020a)
5	Staphylococcal Enterotoxin B (SE-B)	Hollow gold nanospheres synthesized with cobalt nanoparticles used as SERS substrate were drop casted on the nitrocellulose membrane of LFA strip.	Anti-SEB antibody	EDC-NHS linker chemistry	-NA-	0.01 ppb	Optimization of cobalt nanoparticle size may result in lesser HGN diameter and better diffusion in the test strip. Detection of SE-B in standard samples was studied, but the presence of any interferents may significantly decrease the sensitivity.	( Hwang et al., 2016)

### 10.4 Bacteria, viruses and other whole cells

**Figure 27.**
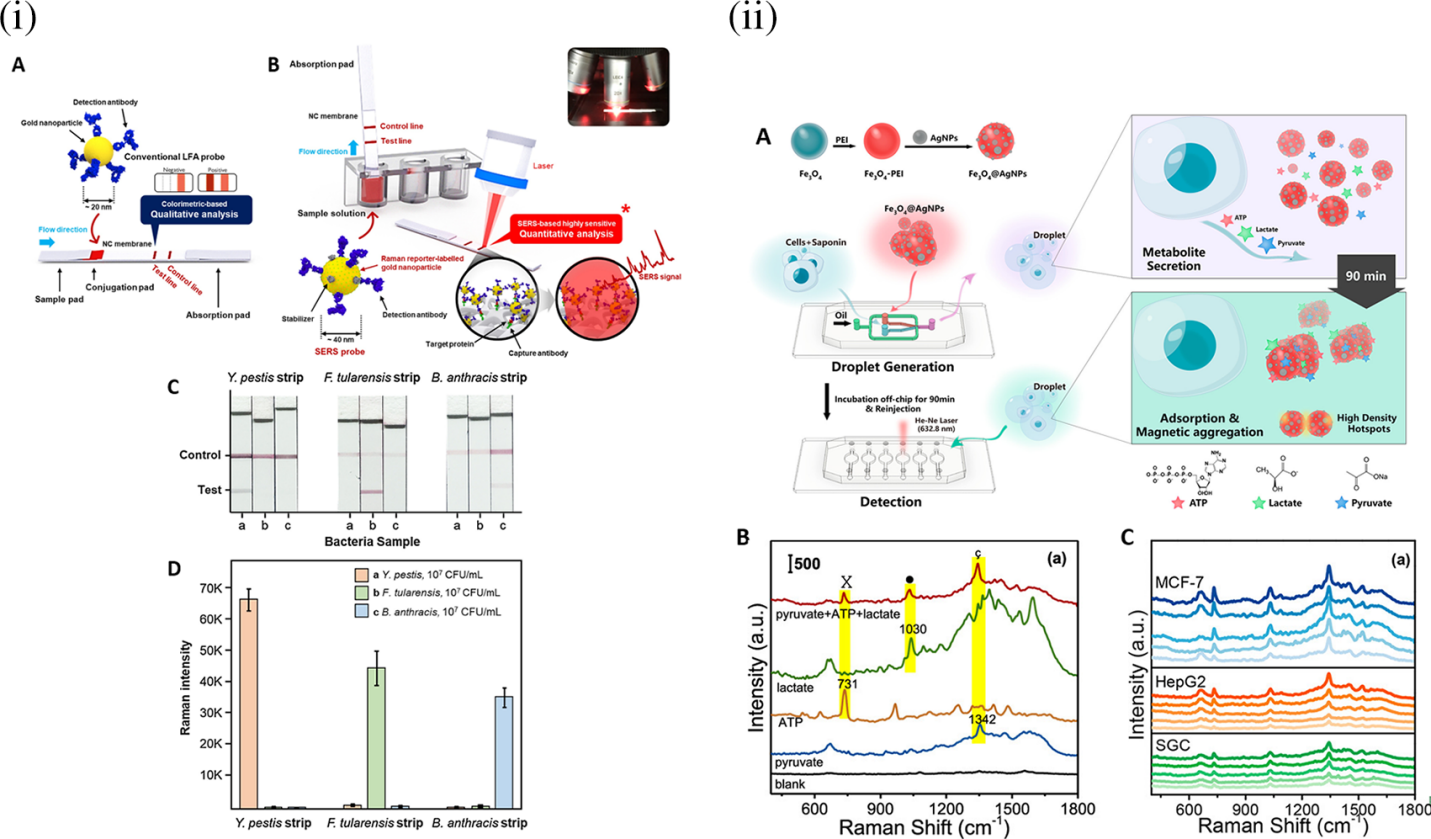
Disposable SERS systems for specific detection of whole cells (i) Schematics of (A) conventional LFA strip (B) SERS-based LFA strip. (C) Images of three strips and (D) SERS signal intensity for *Y. pestis*, *F. tularensis*, and *B. anthracis*. (This figure has been reproduced with permission from ( R. Wang, Kim, et al., 2018b), Copyright from (2018) Sensors and Actuators B: Chemical), (ii) (A) Overview of SERS-microfluidic droplet platform for single-cell encapsulation and simultaneous detection of three metabolites produced by a single cell using Fe _3_O _4_@AgNPs nanocomposite, (B) Fe _3_O _4_@AgNPs nanocomposite for solo metabolite detection (ATP (731 cm ^–1^), lactate (1030 cm ^–1^), and pyruvate (1342 cm ^–1^), respectively), (C) SERS of cellular metabolites content in SGC, HepG2, and MCF-7 cells (one to five cells from bottom to top, respectively) encapsulated in individual droplet. (This figure has been reproduced with permission from ( D. Sun et al., 2019), Copyright from (2019) ACS Analytical Chemistry).

Microbes, specifically viruses, pose a major hazard to the healthcare sector because of their rapid replication cycles and minimal access to instruments capable of ultrasensitive detection. Real-time detection from complex samples using conventional techniques cannot limit bacterial replication and improve the chances of infection after entering a live host. Thus, there is an urgent need for rapid and ultrasensitive detection in real samples for instant treatment of the host ( Sreelakshmi et al., 2024). A label-free bacterial SERS detection method was developed by Gao et al., using the selective growth of AgNPs on the bacterial outer membrane conjugated with aptamers. Improved SERS signal by aptamer@AgNPs complex resulted in exceptional LoD of 1.5CFU/mL using micro-Raman system ( W. Gao et al., 2017). Additionally, the use of lateral flow assay (LFA)-based strips for the qualitative determination of the analyte of interest is essential for POC diagnostics, which are cost-efficient, reliable, reproducible, and highly sensitive ( Ryu et al., 2010). Wang et al. tested a sandwich immunoassay using antibody-conjugated AuNPs with reporter molecules for bacterial detection, as shown in Figure 27(i). Malachite green isothiocyanate (MGITC) in the test line indicated the presence of target bacteria binding to antibodies on the AuNPs. The test concentration was maintained at 10 ^7^ CFU/mL for all studies. This rapid detection (~15 min) method showed an LoD of 357 CFU/mL for *B. anthracis* strain with a minimal sample ( R. Wang, Kim, et al., 2018b).

Most recent epidemics are caused by viruses that possess multiple surface proteins and genetic material, which are reliable biomarkers for the detection of their presence during disease prognosis and treatment. A mini microfluidic platform named VIRRION (virus capture with Raman Spectroscopy detection and identification) was developed using arrays of carbon nanotubes with different filtration permeabilities for high-throughput virus capture and rapid SERS detection. This method can be employed for multiple-virus capture and detection on a chip by SERS using a Raman system ( Yeh et al., 2020). Another method with labelled detection of the virus was developed by Shen et al., based on the immunoassay method using an LFA strip-based reaction for wild-type pseudorabies virus from clinical samples of pig tissue. This method detects glycoprotein E-specific PCR, which differentiates between the wild-type PRV and gE-deleted vaccine. This method requires approximately 15 min and has an LoD of 5ppm and linear detection range of 41-650 ppm( Shen et al., 2019).

Whole cell detection is considered a need of the hour to identify the presence of circulating tumor cells in the bloodstream, which helps in the early detection of cancer metastasis. This early diagnosis involves a liquid biopsy instead of a tissue biopsy. CTC (circulating tumor cells) detection from millions of blood cells is expensive and minute sensitivity needs advancements in the technique for rapid and sensitive detection techniques. In a study by Wu et al., a SERS method with gold nanostructures of different geometries, that is, AuNSs, AuNRs, and AuNPs, was used for CTC detection in liquid biopsy samples. 4-Mercaptobenzoic acid (4-MBA) dye was used with the nanoparticle to induce stability of bovine serum albumin (BSA) and to increase specificity towards conjugation with CTC; folic acid was used. AuNPs displayed the highest specificity, with an LoD of 1-100 cells/mL ( X. Wu et al., 2016). In another study by Sun et al., a SERS-based label-free droplet microfluidic sensor was developed for the detection of multiplex metabolites at the single-cell level, as shown in Figure 27. (ii). For the detection of excessive metabolites (lactate, pyruvate, and ATP) secreted from CTC by magnetic isolation of CTCs from complex samples, 400 nm Fe _3_O _4_ magnetic microspheres were decorated with 30 nm AgNPs ( D. Sun et al., 2019). Table XVI illustrates some of the disposable substrates used for the SERS-based detection of bacteria, viruses, and whole cells.

**Table XVI.** Disposable substrates for SERS-based detection of bacteria, viruses and whole cells.

Sl. No.	Analyte	Method	(Bio) Receptor	Linker chemistry	Sample	LOD	Remarks	Ref.
1	* E. coli* O157, *S. typhimurium* and *S. aureus*	Glass substrate for SERS substrate synthesis by the assembly of silver nanocrystals into silver nanospheres	-NA-	-NA-	-NA-	10 CFU/mL	The use of a bioreceptor with the sensor can significantly improve the specific detection of the target bacteria. The sensitivity of this sensor depends on the assembly of silver nanocrystals that further affects the reproducibility.	( Y. Wang et al., 2010)
2	* S. aureus*	POC device for multiplex bacterial detection using 3D membrane -like SERS nanosticker synthesized by graphene oxide@Au/Ag	Antibody	EDC-NHS linker chemistry	Urine and sputum samples	10 ^4^ & 10 ^2^ CFU/mL in urine & sputum samples respectively	Good sensitivity was achieved using the as -synthesized SERS substrate, but the significance of graphene oxide is not specified.	( C. Wang et al., 2022)
3	H1N1 and HAdV	SERS -based LFA strip decorated with Fe3O4@AgNPs as magnetic SERS nanotags for viral detection	Antibody	EDC-NHS linker chemistry	Sputum	50 and 10 pfu/mL in culture samples & 10 ^6^ pfu/mL	Sensitivity is affected by the uniformity of the core-shell NP uniformity further affecting the sensor reproducibility.	( C. Wang et al., 2019a)

In conclusion, despite the insignificant size and molecular weight of the analyte, these disposable material-based sensors showed higher sensitivity when anisotropic nanomaterials were used, which improved the nanostructure’s local electric field due to the edge effect and thus contributed to the amplification of the Raman signal.

## 11. Hybrid SERS systems

Traditional analytical techniques typically involve elaborate or complex procedures, sample pretreatment steps, and costly and sophisticated instrumentation. Consequently, there is a greater need for analytical methods that go beyond centralized laboratory-based methods to enable POU testing. In the recent past, SERS and electrochemical (EC) sensors have been considered standard techniques in this regard. The EC-SERS characterization tool was expected to address the shortcomings of the two analytical techniques and strive to improve specific features by integration. It has gained rapid attention for qualitative sensing applications and has enabled notable advancements in the domains of advanced materials and life sciences. Integration of EC sensors with SERS may improve sensitivity by manipulating the target analyte adsorption on the SERS-substrate surface, substrate surface activation by oxidation/reduction cycles (ORC), and synthesis of SERS-active nanostructures or nanocomposites with various geometries.

An interesting method proposed by Ibáez et al. for the electrochemical activation of gold SPEs allows the detection of trace-level pesticides. The proposed sensing strategy involves preconcentration of the drop solution on the electrode surface for 15 min at 34 °C, followed by electrochemical activation with SERS-active nanostructures. The obtained SERS spectra showed prominent peaks at 1380 cm ^−1^ for thiram and 1107 cm ^−1^ for imidacloprid. These distinct bands enabled improved detection ability with an LoD of 2.4 mgL ^−1^ thiram and 25 mgL ^−1^ imidacloprid. Tap water samples were also analyzed and appropriate results were obtained to demonstrate the capabilities of the proposed method ( Ibáñez et al., 2021). Another interesting SERS system, developed by Zhao et al., synthesized multilayered EC-SERS active substrates that can potentially increase the localized plasmonic field during SERS measurements. The authors used a controlled growth citrate reduction method to synthesize monodisperse Au/Ag nanoparticles. Further, these nanoparticles were multilayer-deposited on the carbon-based working electrode of commercially available screen-printed electrodes to obtain an EC-SERS active substrate (Au/Ag substrate). Multilayered Au/Ag substrates were used for the quantitative detection of uric acid in a 0.1 M NaF solution and synthetic urine. A good linear relationship was observed between the uric acid concentration and the EC-SERS signal intensity in the clinically relevant range (0.1-1.0 mM), and it can be employed in routine testing of early stage diagnosis of preeclampsia ( L. Zhao et al., 2015). However, this method requires multiple depositions of monodisperse nanoparticles to fabricate the Au/Ag substrate, which may result in irregularly stacked SERS-active substrates.

Despite the optimization of the geometry and properties of the SERS-active substrate, the adsorption of the analyte on the substrate surface is a significant factor for efficient SERS analysis. In an interesting method proposed by Zhu et al., a miniaturized spectroelectrochemical system was developed, which relies on the adsorption of the target analyte on the SERS active screen-printed electrode with a coin-sized chip and obtained the Raman spectra of aminoglutethimide (AGI) on a silver-sputtered electrode. The EC-SERS spectra of the AGI molecules revealed different adsorption modes on the substrate at different potentials. With an applied potential of -400 mV, the EC-SERS peak intensities of the aniline and glutarimide moieties were substantially improved, indicating a bidentate interaction between the AGI molecule and the substrate. The defined applied potential showed intense peaks at 1147 cm ^−1^ (C-H/CH _2_ in-plane bending), 1302 cm ^−1^ (CH _2_ wagging), and 1566 cm ^−1^ (NH _3_^+^ rocking), while the 1147 cm ^−1^ peak intensity was 30-fold higher than that of the standard SERS signal; therefore, it was considered for further experimentation. The developed system showed an LoD of 40 ng/mL and a linear detection range of 1 x 10 ^−5^ M to 2 x 10 ^−7^ M with a linear curve R ^2^ of 0.98. ( Z. Zhu et al., 2018). Another novel hybrid SERS system for assessing real-time interactions based on EC-SERS was demonstrated by Hernandez et al. in 2020. Time-resolved Raman spectroscopy was used for quantitative analysis using in situ-synthesized EC-SERS substrates. This detection technique used a sample volume of 50l and provided direct quantification results within 50 seconds, for nicotinamide detection in complex samples. This strategy possesses the benefit of examining the analyte-substrate interaction during the complex formation, thereby assisting in optimizing the conditions for the target analyte detection, and producing well-defined and repeatable Raman spectra. Selective detection of nicotinamide from a multivitamin complex, yielded impressive results (R ^2^ = 0.99; %RSD < 9%) ( Hernandez et al., 2020). Therefore, the development of Hybrid-SERS systems can open new avenues for improved, rapid and robust sensing. These integrated systems may essentially combine their specific benefits and aid in detection of trace analytes. However, these systems are limited with challenges such as reproducibility and electrode fouling. Future advancements, in synthesis of new electrode materials that can aid in simultaneous signal amplification and development of integrated microfluidics for real-time monitoring can translate these systems for POU sensing.

## 12. Portable SERS-based sensory systems

**Figure 28.**
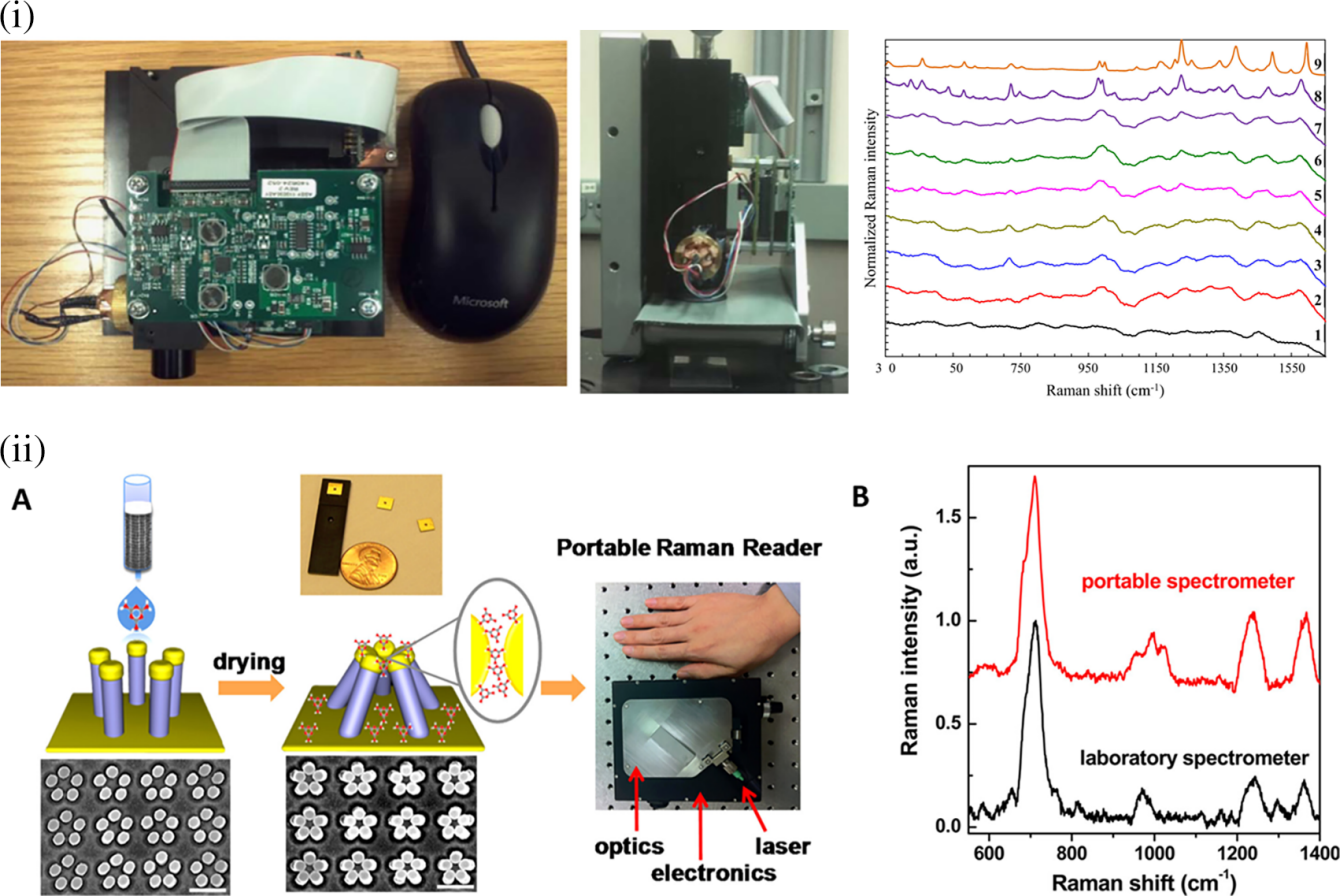
Portable SERS-based sensing systems (i) Schematic representation of SERS measurements with portable SERS system (left) and corresponding SERS spectra of Sudan I in paprika extracts (right) (This figure has been reproduced with permission from ( F. Gao et al., 2015), Copyright (2015) Talanta) (ii) (A) Melamine detection in milk on nanofinger substrate chips using the custom-built Raman spectrometer, (B) SERS spectra comparison of 1 ppm melamine in milk using custom-built Raman spectrometer (black) and portable SERS system (red). (This figure has been reproduced with permission from ( A. Kim et al., 2012), Copyright (2012) Analytical Chemistry).

Portable SERS-based systems are essential for on-site detection of whole cells, biomolecules, pharmaceuticals, and trace chemicals. Their portable and compact design enables rapid, efficient, sensitive, and selective on-site sensing, in the domains of food quality control, environmental monitoring, and biomedical diagnostics. SERS-based sensing of small or large molecules has evolved significantly over the past decades. Despite these advancements, the sensing of any analyte by Raman spectroscopy requires extensive resources and trained personnel for use on a commercial scale. Therefore, the portability, user friendliness, and robustness of the developed sensor are of great concern. In a study by Gao. F et al. developed and tested a custom handheld SERS-based sensor for the sensing of Sudan I in paprika extracts. The developed biosensor combines thin-layer chromatography (TLC), molecularly imprinted polymers (MIPs), and SERS using a Sudan I template on methacrylic acid monomers. This portable custom-made SERS spectrometer was equipped with a backscattering probe for the collection of Raman spectra. The light source used was a 785 nm laser diode with 100-mW laser power and holographic grating for efficient light dispersion. Figure 28. (i) depicts a schematic representation of the custom-made portable SERS system for Sudan I detection in the spectral range of 350-1650 cm ^−1^ ( F. Gao et al., 2015). Another study by Leiber et al. developed a portable SERS system for dermatological applications. The developed SERS system was equipped with NIR-optimized objective lens with < 0.1μm resolution, a 100 μm-core fiber optic probe connected to 825 nm laser diode, and diffracting grating for light dispersion. All dermatological tests were optimized with respect to the cellophane Raman spectra intensities. Prior to testing the dermatological samples, the Raman spectra of acetamidophenol were measured and showed significant equivalence with a benchtop Raman microspectrometer with similar instrumentation ( Lieber & Mahadevan-Jansen, 2007). In another study by Kim et al., a custom-built SERS system was developed for melamine detection in filtered milk samples. Gold nanofinger chips were fabricated by nanoimprint lithography (NIL) on a Si wafer and further diced to 5 mm × 5 mm chips, which were used as SERS substrates. The developed SERS system was equipped with a 785 nm laser diode for a specific illumination of 100 μm area on the developed SERS substrate and nitrogen-cooled CCD detector. Figure 28. (ii) (A) shows the size of the custom-built Raman reader with pentameric nanofinger substrate chips, and (B) illustrates the comparative Raman spectra of the custom-built Raman reader with an upright confocal Raman microspectrometer ( A. Kim et al., 2012).

## 13. AI enabled SERS systems: A way forward

The integration of artificial intelligence (AI) with SERS-based sensing systems is predominantly increasing because of the improved accuracy of analyte(s) detection from a SERS spectrum or for specific detection from complex specimens. Machine learning (ML) uses algorithms for data analysis, pattern identification, and forecast of outcomes ( Y. Liu et al., 1993). However, these AI and ML algorithms are extensively used in data interpretation and process optimization in complex experimental processes, design of SERS-active substrate and its fabrication for signal enhancement ( Banaei et al., 2019; Nguyen et al., 2020). The assimilation of computational processing with experimental data can signify the optimal morphology of the nanostructures for the improvement of Raman signals.

Classification of SERS spectral data is possible with supervised learning algorithms, such as support vector machines (SVM) ( Widjaja et al., 2008), random forests (RF) ( Ham et al., 2005), and neural networks ( Sigurdsson et al., 2004) for real-time analyte detection from complex specimens. In addition, data classification and neural networks, such as convolutional neural networks (CNNs) and recurrent neural networks (RNNs), can also learn the classification of hierarchical patterns from the spectra ( Acquarelli et al., 2017) and temporal classifications from sequential data, ( Boydston-White et al., 2006) which is crucial for real-time analysis. Typically, spectral patterns are identified with clustered algorithms, such as K-means for data partitioning ( Lopez-Reyes et al., 2014), mean shift for shifting the data points to the nearest peak, and Gaussian mixture models (GMM) that estimate the distribution parameters to allocate data points to the clusters ( Hsu et al., 2012). Additionally, pattern identification and clustered algorithms, such as k-means clustering and hierarchical clustering, are utilized in anomaly detection ( Chawla & Gionis, 2013), along with spectral grouping and visualization as dendrograms ( Sander et al., 2003). Quantitative analysis of the target analyte concentration in a sample is predicted using an artificial neural network (ANN) ( Lopez-Reyes et al., 2014) and partial least square regression (PLSR) ( Kachrimanis et al., 2007). Feature extraction and selection from multidimensional data are detected using principal component analysis (PCA) ( Kuncheva & Faithfull, 2014) and autoencoders ( Pyo et al., 2020) for low-dimensionality representation. Experimental parameters, such as the laser wavelength, laser power, and morphology of the substrate, can be optimized using genetic algorithms ( Hennessy & Kelley, 2004). Furthermore, the robustness and reproducibility of the system may be optimized for improved efficiency under varying conditions by quantification of uncertainty with probabilistic modelling using Bayesian inference. ( Han & Ram, 2020; S. Li et al., 2011).

The integration of AI with SERS may simplify the design of SERS systems and make them relevant for point-of-use. ML algorithms can conduct automatic spectral analyses, enabling precise analyte identification. These are promising avenues of research for advancements in analytical chemistry and design of portable systems.

## 14. Conclusion

This article provides a comprehensive overview of recent advances in the design of SERS substrates and their applications in sensing, medical diagnostics, food quality control domains, and environmental monitoring with its characteristic fingerprinting ability. A notable development in the use of commercial-grade disposable materials for point-of-use SERS-based systems offers a quick, sensitive, and affordable real-time analysis. Disposable substrates assist in single-use applications as they are made of inexpensive materials, such as paper or polymers, and potentially surpass the need for elaborate cleaning protocols. These disposable materials can assist in the development of POU sensing devices that require robust equipment design, compactness, user-friendly operation, and minimal sample processing. Tailoring the substrate’s surface by customizing the physical properties, generating regularly ordered periodicity and surface hydrophobicity, or chemical properties by coating, doping, or embedding the surfaces with plasmonic nanoparticles provides improved sensitivity and selectivity, thereby ensuring efficient target analyte detection. Analyte capture and specificity are improved by surface functionalization, and scalable production is made possible using techniques such as inkjet printing. SERS based detection also shows a great promise in integration with point-of-care technologies for the development of wearable devices and POU devices for deployment in resource-limited settings. The use of statistical methods, such as correlation analysis and multivariate approaches, and machine learning algorithms, such as supervised, unsupervised and hybrid learning algorithms, facilitate the analysis of complex SERS data sets to decipher intricate signals from biological samples. The integration of microfluidic channels and sample preconcentration may further improve the analytical performance. In summary, disposable material-based plasmonic substrates used for POU SERS-based sensing represent a viable method for quick, accurate, and useful analyses in a variety of biological, environmental, and security applications. Further investigation and advancement will augment the comprehensibility and expand the applicability of this technology across several domains.

## Ethical approval and consent

Ethical approval and consent were not required.

## Data Availability

No data are associated with this article.
