# Supplementary material for: Recent advances in the design of SERS substrates and sensing systems for (bio)sensing applications: Systems from single cell to single molecule detection
Source: F1000Res. 2025 Jun 24;13:670. Originally published 2024 Jun 21. [Version 3] doi: 10.12688/f1000research.149263.3 (PMC12009482; doi:10.12688/f1000research.149263.3)
Supplement: Peer review history

# Reviewer report: Reviewer response for version 2

**DOI:** 10.5256/f1000research.179149.r371534

**Published:** 2025-04-24

**Authors:** Nabadweep Chamuah

**Affiliations:** 1 Department of Electronics, Digboi College, Assam, India

The authors addressed all the comments effectively. The revised article improved its readability and clarity. It is expected to provide readers with a clear understanding of SERS and its recent advancements.

Is the review written in accessible language?

Yes

Are all factual statements correct and adequately supported by citations?

Partly

Are the conclusions drawn appropriate in the context of the current research literature?

Partly

Is the topic of the review discussed comprehensively in the context of the current literature?

Yes

Reviewer Expertise:

SERS, Bio sensing, Smartphone Based sensing, Optical Sensing.

I confirm that I have read this submission and believe that I have an appropriate level of expertise to confirm that it is of an acceptable scientific standard.

# Reviewer report: Reviewer response for version 2

**DOI:** 10.5256/f1000research.179149.r374420

**Published:** 2025-04-19

**Authors:** Ranjan Ganguly

**Affiliations:** 1 Department of Power Engineering, Jadavpur University, Kolkata, West Bengal, India

This is a well-written review article describing the hallmarks of Surface enhanced Raman spectroscopy and how the technique can be harnessed for biosensing at different length-scales. The article provides a sound coverage of the fundamental precept of Raman scattering, making it an excellent starting document for any researcher in the field. The basic building blocks for point-of-use SERS is discussed very thoroughly so that one can develop a system based on the information presented here. The literature search has not only been meticulous, but the insightful figures, tables and critical remarks are meant to serve the community at different levels of expertise. Salient challenges in the use of different substrates, and the state of art to overcome these bottlenecks have been discussed at a significant depth. The requirements of SERS systems for various point-of-use applications and the necessary optimization strategies for improved sensitivity, specificity, selectivity and scalability for mass-deployment for POC applications have been nicely deliberated with abundant supporting literature. The review puts the SERS-based detection strategy nicely in the overall landscape of future biosensing. Overall, the review has been a highly comprehensive one and I recommend indexing it in the current form.

 Minor modifications suggested:

1. The third sentence of the Abstract could be modified as “However, the phenomenon do not hold potential for its direct use in sensing applications, since the generation of the Raman scattered photons is inherently low.”
2. field deployable should be field-deployable.
3. The line above Eq 1 is repeated. Delete the one immediately above the equation.
4. The paragraph above Eq. 2 has repetition. For example, the sentence “Hence, the Stokes process results in a lower vibration energy (hωv), while the anti-Stokes process is associated with a higher vibration energy (hωv)” may be deleted.
5. Define E _incident_ in Eq 3
6. Please expand captions of Figure 21(ii); 22(i-f,g), (ii B,C), 23, 25, 26 and 27(ii). Even if the original articles did not have the expanded caption, you can provide it. The copyright permission is, I guess, valid for replicating the figure, but not the caption. 

Is the review written in accessible language?

Yes

Are all factual statements correct and adequately supported by citations?

Yes

Are the conclusions drawn appropriate in the context of the current research literature?

Yes

Is the topic of the review discussed comprehensively in the context of the current literature?

Yes

Reviewer Expertise:

Microfluidics; surface science

I confirm that I have read this submission and believe that I have an appropriate level of expertise to confirm that it is of an acceptable scientific standard.

# Reviewer report: Reviewer response for version 1

**DOI:** 10.5256/f1000research.163708.r300697

**Published:** 2024-08-21

**Authors:** Debasmita Mondal

**Affiliations:** 1 Department of Electrical and Instrumentation Engineering, Thapar Institute of Engineering and Technology, Patiala, Punjab, India

The review article "Recent advances in the design of SERS substrates and sensing systems for (bio)sensing applications: Systems from single cell to single molecule detection" by Tadi et al. provides an extensive review of the surface enhanced raman spectroscopy technique and its application in the area of biosensing. The manuscript is well written and comprehensively discusses SERS starting from the basics of the mechanism, its applications and sensing substrates to the gradual advancements in the field, finally the current trends. This review article provides a broad overview and understanding of SERS for readers seeking general overview as well as in-depth understanding of the topic.

 Some minor points which can improve the readability of the paper are:

 1. Some of the variables are not defined before use; such as λ _S_, λ _L_, E _S_, E _L_ etc. in page 3 of the manuscript.

 2. Resolution of Figure 1 is not good.

 3. *et al. *should always be in italics.

Is the review written in accessible language?

Yes

Are all factual statements correct and adequately supported by citations?

Yes

Are the conclusions drawn appropriate in the context of the current research literature?

Yes

Is the topic of the review discussed comprehensively in the context of the current literature?

Yes

Reviewer Expertise:

Biosensors

I confirm that I have read this submission and believe that I have an appropriate level of expertise to confirm that it is of an acceptable scientific standard.

# Reviewer report: Reviewer response for version 1

**DOI:** 10.5256/f1000research.163708.r295700

**Published:** 2024-08-16

**Authors:** Nabadweep Chamuah

**Affiliations:** 1 Department of Electronics, Digboi College, Assam, India

The article entitled “ **Recent advances in the design of SERS substrates and sensing systems for (bio)sensing applications: Systems from single cell to single molecule detection**” by Sai et.al provides a comprehensive overview of recent advances in Surface-Enhanced Raman Spectroscopy (SERS) substrates and sensing systems, highlighting their applications in biosensing. It effectively summarizes the mechanisms of SERS, the design of substrates, and their applications in detecting single cells and molecules. Few noticeable strength of the article are-

 1. The article covers a wide range of topics related to SERS, including the historical background, mechanisms, and recent advancements in substrate design and applications. This provides readers with a broad understanding of the field.

 2. It includes detailed discussions on the mechanisms of Raman scattering and the factors influencing SERS enhancement, which are crucial for readers seeking an in-depth technical understanding.

 3. The paper highlights recent advancements and research trends in SERS, particularly in the design of substrates and the use of SERS in biosensing applications.

 However, there are several areas where the article can be improved in terms of clarity, depth, and critical analysis. The authors can consider the following points to improve the impact of the article.

  

1. The abstract should be more focused, clearly stating the main objectives, findings, and implications of the article. It currently reads more like an introduction and could benefit from summarizing key results or conclusions.
2. The sentence “Plasmonic nanoparticles, called hotspots, exhibit localized surface plasmon resonance (LSPR).” Is somewhat confusing and can be rephrased as Plasmonic nanoparticles exhibit localized surface plasmon resonance (LSPR), and when they are closely spaced, they create 'hotspots' where the electromagnetic field is significantly enhanced.
3. The EF of SERS has been reported up to 10 ^14^. Authors can follow this article for more detail. https://doi.org/10.1038/s41598-018-36491-0
4. While the introduction provides historical context, it could be more focused on framing the current challenges and the specific advancements the article will discuss. A clearer statement of purpose and scope would help guide the reader.
5. The article primarily summarizes existing research but shows insufficient critical analysis or evaluation of the studies discussed. Including a discussion on the limitations or challenges associated with current SERS substrates and sensing systems would provide a more balanced view.
6. Discussing the potential pitfalls or limitations of SERS, such as reproducibility issues or limitations in real-world applications, would add depth to the analysis.

 

1.  The discussion on SERS mechanisms could be expanded to include more detailed explanations of the electromagnetic and chemical enhancement processes and their implications for substrate design.

 

1. While the article mentions various substrate designs, it could delve deeper into the specific materials and nanostructures used, as well as their advantages and limitations.

 

1. The section 5.1 need to restructure and should be supported by proper references.

 

1. The table I, II and IV should be supported by proper references. Change the heading of figure IV considering plasmonic nanostructures are not hotspot.  

 

1. In “One of the widely accepted theories for CE is the charge transfer (CT) mechanism proposed by the SERS pioneer Andreas Otto, where the molecule is chemically adsorbed on the substrate, which subsequently changes the molecular polarizability, thereby enhancing the Raman spectrum  Check if it is Raman spectrum or Raman scattering.

 

1. In the discussion of paper substrate the authors can also include following papers

https://doi.org/10.1016/j.saa.2023.122610,

**DOI** 10.1088/1361-6463/aa8fef

  

1. Consider rephrasing of following sentences for better readability-

 

1. **Table I 5 ^th^ row-** They don't fluoresce, but poor Raman scattering efficiency as compared to visible range lasers.
2. **Section 3.3 last paragraph- **The cooling of the photodetector in a Raman system reduces the dark noise, which can be attributed to the dark current  These dark currents influence the photodetector temperature, which requires intermediate cooling. Cooling the photodetector also aids in reducing background noise and improving the SNR of the sensing system.
3. **Section 10 last paragraph-** Thus, the measured Raman spectra should be free of trace impurities to avoid the background signal and provide a Raman signal.

 

1. The article should conclude with a discussion on future research directions and emerging trends in SERS. Identifying areas where further research is needed would provide valuable insights for researchers in the field.

While the article offers a solid overview of recent advances in SERS substrates and sensing systems, it would benefit from greater focus, critical analysis, and depth in discussing specific advancements and challenges. Enhancing the clarity, organization, and critical evaluation of the content will strengthen the article's impact and usefulness to researchers and practitioners in the field.

Is the review written in accessible language?

Yes

Are all factual statements correct and adequately supported by citations?

Partly

Are the conclusions drawn appropriate in the context of the current research literature?

Partly

Is the topic of the review discussed comprehensively in the context of the current literature?

Yes

Reviewer Expertise:

SERS, Bio sensing, Smartphone Based sensing, Optical Sensing.

I confirm that I have read this submission and believe that I have an appropriate level of expertise to confirm that it is of an acceptable scientific standard, however I have significant reservations, as outlined above.

## References

[1] : Detection and analysis of rotavirus in clinical stool samples using silver nanoparticle functionalized paper as SERS substrate. *Spectrochim Acta A Mol Biomol Spectrosc* .2023;295: 10.1016/j.saa.2023.122610 122610 10.1016/j.saa.2023.122610 36921516

[2] : Phase controlled SERS enhancement. *Sci Rep* .2019;9(1) : 10.1038/s41598-018-36491-0 744 10.1038/s41598-018-36491-0 30679465 PMC6346009

[3] : SERS on paper: an extremely low cost technique to measure Raman signal. *Journal of Physics D: Applied Physics* .2017;50(48) : 10.1088/1361-6463/aa8fef 10.1088/1361-6463/aa8fef

# Response

**Published:** 2025-03-06

**Authors:** Sai Ratnakar Tadi

**Affiliations:** Mechatronics, Manipal Academy of Higher Education, Manipal, Karnataka, India

**Response to reviewer comments**

 Thank you for the critical review of the manuscript, we have tried to incorporate all the suggestions wherever possible.

**Q 1. The abstract should be more focused, clearly stating the main objectives, findings, and implications of the article. It currently reads more like an introduction and could benefit from summarizing key results or conclusions.**

 Response:

 The abstract has been modified to a technically precise form incorporating the current state of work and future directions.

 The revised abstract is as follows:

 The Raman effect originates from spontaneous inelastic scattering of photons by matter.  These scattered photons provide a characteristic fingerprint of this matter, and are extensively utilized for chemical and biological sensing. The inherently lower generation of these Raman scattered photons, do not hold potential for their direct use in sensing applications. Surface-enhanced Raman spectroscopy (SERS) overcomes the low sensitivity associated with Raman spectroscopy (SERS) and assists the sensing of diverse analytes, including ions, small molecules, inorganics, organics, radionucleotides, and cells. Plasmonic nanoparticles exhibit localized surface plasmon resonance (LSPR), and when they are closely spaced, they create hotspots where the electromagnetic field is significantly enhanced. ￼This amplifies the Raman signal and may offer up to a 10 ^14^-fold SERS signal enhancement. The development of SERS active substrates requires optimization of critical features such as surface periodicity, hotspots, mitigation of autofluorescence, tuning of surface hydrophilicities, use of specific (bio) recognition elements with suitable linkers and bioconjugation chemistries, and use of appropriate optics to obtain relevant sensing outcomes in terms of sensitivity, cross-sensitivity, limit of detection, signal-to-noise ratio (SNR), stability, shelf-life, and disposability. This article comprehensively reviews recent advancements on the use of disposable materials such as commercial grades of paper, textiles, glasses, polymers, and some specific substrates such as blue-ray digital versatile discs (DVDs) for use as SERS-active substrates for point-of-use (POU) sensing applications. The advancements in these technologies have been reviewed and critiqued for analyte detection in resource-limited settings, highlighting the prospects of applications ranging from single-molecule to single-cell. We conclude by highlighting the prospects and possible avenues for developing viable field deployable sensors holding great potential in environmental monitoring, food safety, and biomedical diagnostics.

**Q 2. The sentence “Plasmonic nanoparticles, called hotspots, exhibit localized surface plasmon resonance (LSPR).” Is somewhat confusing and can be rephrased as Plasmonic nanoparticles exhibit localized surface plasmon resonance (LSPR), and when they are closely spaced, they create 'hotspots' where the electromagnetic field is significantly enhanced.**

 Response:

 The specified statement has been rephrased as ‘Plasmonic nanoparticles exhibit localized surface plasmon resonance (LSPR), and when they are closely spaced, they create 'hotspots' where the electromagnetic field is significantly enhanced’ in the abstract of the modified manuscript.

**Q 3. The EF of SERS has been reported up to 10 ^14^. Authors can follow this article for more detail. https://doi.org/10.1038/s41598-018-36491-0**

 Response:

 The said article has been cited in the abstract of the revised manuscript and the typical range of EF has been modified.

**Q 4. While the introduction provides historical context, it could be more focused on framing the current challenges and the specific advancements the article will discuss. A clearer statement of purpose and scope would help guide the reader.**

 Response:

 The suggestions have been incorporated as follows:

1. Current challenges with existing fabrication strategies are discussed in section 5 (Considerations in design of disposable SERS substrates).

Section 5.1 reviews the SERS hotspots, with a comprehensive discussion on the materials, strategies used in hotspot engineering with 0D, 1D, 2D and 3D materials, and a critical analysis of generating homogeneous hotspots to effectively improve the EF.

 Section 5.2 discusses the recent advancements in strategies for generating tailored hydrophobic substrates that minimize the need for large sample volume and standard errors.

 Section 5.3 comprehensively reviews the advancements in generating substrate periodicity and their significance to impart hydrophobicity, in addition to enhanced plasmonic field density.

  

1. The specific advancements are comprehensively discussed in section 4 (SERS systems for point-of-use sensing) and section 11 (Hybrid SERS systems).

Section 4 summarizes the recent specific advancements in the development of point-of-use SERS sensing systems, such as integration of the transduction platforms for complementary data acquisition (Choi et al., 2017), laser induced catalysis (C. Song et al., 2020), and enzyme-induced digestion of bioreceptor for specific detection (Gracie et al., 2014).

 Section 11 reviews the recent developments of hybrid SERS systems, such as electrochemical-SERS (EC-SERS) that improve the sensitivity and activate the substrate by oxidation/ reduction cycles (ORC) (Ibáñez et al., 2021) and lateral flow assay (LFA) based-SERS with regulated sample flow with great potential for on-site use (C. Wang et al., 2019a)

 We hope that in its revised form presents better clarity to the readers.

**Q 5. The article primarily summarizes existing research but shows insufficient critical analysis or evaluation of the studies discussed. Including a discussion on the limitations or challenges associated with current SERS substrates and sensing systems would provide a more balanced view.**

 Response:

 The limitations associated with current SERS substrates and sensing systems are comprehensively discussed herein:

 A study by Dong-Jin Lee et al *., *in 2019, detected thiram using paper as the sensor substrate (Dong-Jin Lee & Dae Yu Kim, 2019). However, the study did not include real sample testing, as the presence of interferents or other structurally analogous molecules can result in false positives. This limitation can be overcome by using a bioreceptor that retains the specificity even in complex organic samples. In addition, cellulose-based paper substrates are associated with a major limitation of autofluorescence because of the presence of organic materials, which may be mitigated using higher-wavelength lasers and optics that block fluorescence.

 Robinson et al. conducted a study in 2014 for the detection of 4, 4’-Bipyridine (4, 4’-BiPy) using novel fabric chips made of Zari fabric (metal coated over silk fabric). However, the calibration data suggests higher relative standard deviation (RSD) with large standard errors at lower analyte concentrations. In addition, no real sample testing was conducted, which can significantly alter the results owing to pH, the presence of other biomolecules, and other organic loads (Robinson et al., 2015).

 Another study on the detection of malachite green (MG) on fish scales was reported by Zhao et al. in 2018 (X. Zhao et al., 2018). However, the detection limit of the developed sensor varied significantly with sensing MG on the real sample; therefore, efficient and specific detection of MG in a complex organic specimen can be challenging. The use of a specific bioreceptor may improve the sensitivity and specificity of a sensor. A similar study by Chamuah et al. in 2019, using blue ray-digital versatile discs (BR-DVDs) as SERS substrate for the detection of albumin, creatinine, and urea in urine samples (Chamuah et al., 2019). However, the coherence AuNP absorbance maxima with the 785 nm laser may not be prominent for efficient light scattering from the synthesized nanostructure. The substrate exhibited an exceptional shelf life of 45 days. However, no cross-sensitivity testing has been conducted to ensure specificity in the presence of structurally analogous molecules or other interferents in a complex sample.

 A summary of challenges associated with current SERS substrates and sensing systems are briefly summarized here:

 Limitations of paper-based substrates are:

1. Effect of higher standard error on the substrate’s reproducibility and the effect of aptamer length on specificity of the biosensor ( Hu et al., 2018)
2. Poor specificity for tyrosine detection, due to no bioreceptor conjugation with AgNPs, thus generating Raman signatures of other analytes as well ( M.-L. Cheng et al., 2011)
3. The effect of optical transmittance of the filter paper is only 1% and that of the BNCP is 50%, the SERS spectra of the bottom-side- up cannot be as high as the top-side- up spectra of the substrate ( Parnsubsakul et al., 2020)
4. The improvement in sensitivity with the use of anisotropic noble metal nanoparticles on the ZnO paper discs ( M. Chen et al., 2016)
5. Inconsistent performance of SERS substrate due to AgNPs penetration into the bulk of paper that can be overcome by modifying to hydrophobic surface (Chamuah. N et al., 2017)

Challenges with fabric-based substrates:

1. Non-specific Raman signals of interferents in real matrices due to absence of bioreceptor (D. Cheng et al., 2018)
2. Imprecise quantification at lower analyte concentrations (Lili Kong et al., 2020)
3. Limited reusability and rapid contamination of the cotton swab (Z. Gong et al., 2014)
4. Need for use of anisotropic nanoparticles to improve sensitivity (Cai et al., 2017)

Challenges with polymer-based substrates:

1. Non-specific aptamer resulting in poor specificity for *V. parahaemolyticus *(S. Wu et al., 2019b)
2. Higher probability of false positives with structurally analogous molecules by considering one vibrational peak ( Yao et al., 2018).
3. Lower specificity of molecularly imprinted polymers (MIPs) resulting in high cross-sensitivity ( Y. Xu, Hassan, et al., 2020c).

A comprehensive review of the challenges associated with existing SERS sensing strategies were further discussed in the Remarks column of table V, VI, and VII, in the modified manuscript. All the cited research papers in the mentioned tables were provided with at least one critical remark

**Q 6. Discussing the potential pitfalls or limitations of SERS, such as reproducibility issues or limitations in real-world applications, would add depth to the analysis.**

 Response:

 The potential limitations of SERS-based sensing, such as reproducibility and limitations in real-world applications were comprehensively discussed in section 9 (Some critical design aspects for SERS Biosensors). 

 Another important consideration in SERS biosensor design is the reproducibility of the substrate. Reproducibility was defined as the closeness between individual test results obtained with same material under different testing conditions (IUPAC-Reproducbility,2014). A study suggested the use of a flow system for sample mixing with colloidal substrates to optimize nanoparticle aggregation for SERS-based detection. A sensor designed by integration of a lab-on-chip setup with a microfluidic system for quantification of mitoxantrone showed a good concentration range of 2.5 x 10 ^—9^M to 1 x 10 ^—6^M for plasma and serum. Despite achieving good reproducibility by integrating the flow system into the substrate surface, the sensor requires a complex and expensive setup (McLaughlin et al., 2002). Surface modification of substrates by chemical functionalization of metal surfaces allows for improved reproducibility.

 Additional limitations with real-world applicability of SERS-based biosensors includes the complexity of sample matrix that affects the analyte Raman signal and its sensitivity (Terry et al., 2022a), the substrate shelf-life based on their susceptibility for oxidation or degradation, sensitivity to environmental conditions such as temperature and light that significantly affects bioreceptor performance (Pérez-Jiménez et al., 2020), imprecise quantification due to variability in the signal amplification and unreliable calibrations (Terry et al., 2022b), integration with standard techniques for enhanced analytical performance, and real-time monitoring (Pilot et al., 2019). In summary, though SERS-based sensing offers exceptional sensitivity and specificity, addressing these challenges is crucial for expanding the practical utility of SERS-based sensing systems.

 A comprehensive discussion on the potential limitations, such as reproducibility and real-world applicability were discussed in page 43, section 9 (Some critical design aspects for SERS Biosensors) of the revised manuscript.

**Q 7. The discussion on SERS mechanisms could be expanded to include more detailed explanations of the electromagnetic and chemical enhancement processes and their implications for substrate design.**

 Response:

 The discussion on SERS mechanism was expanded with detailed explanation of the enhancement processes from page 9-12 of section 2 (Surface Enhanced Raman Spectroscopy), as follows:

 Chemical enhancement

 Chemical enhancement (CE) is defined as the amplified Raman scatter signal of a target analyte resulting from the interaction between the adsorbed analyte molecule and the plasmonic nanostructure. It is calculated as the sum of differences in Raman polarizability due to the molecule adsorption onto the metal surface and the charge transfer mechanism, given by equation (4). However, the basis of CE has been the subject of debate for decades, particularly the science that governs the SERS chemical enhancement factor (EF) (Eric C. Le Ru & Pablo G. Etchegoin, 2009). Molecular adsorption can be physisorption or chemisorption with a bond energy of ~40 kJ/mol (Aroca, 2006). Charge transfer in the metal- molecule complex is due to the incident light energy corresponding to the electronic transitions of the molecule with the underlying phenomenon of Resonance Raman Scattering.

 I _Raman_∝1/λ ^4^ |∂α/∂Q| ^2^                                                                                                                                 (4)

 where I _Raman_ is the intensity of the Raman scattering, λ is the wavelength of the incident laser used, α is the polarizability of the molecule, and Q is the normal mode coordinate.

 One of the widely accepted theories for chemical enhancement is the Charge Transfer (CT) mechanism proposed by the SERS pioneer Andreas Otto, where an adsorbed molecule subsequently changes the molecular polarizability, thereby enhancing the Raman scattering (Otto et al., 1992). For example, an incident photon with a frequency ν _inc_, in resonance with the surface-adsorbate complex, causes excitation and return of the metal electron to its ground state because of the CT mechanism. However, if the excited electron resides in the lowest unoccupied molecular orbital (LUMO) for a time duration shorter than the absorbed photons, it will be scattered with dissimilar energy levels than the incident photons (ω _s_ = ω _inc_ — ω _vib_).

 Chemical enhancement can be due to intramolecular resonance (Creighton, 1983), ground-state charge transfer, (Lippitsch, 1984) or resonant charge transfer (Lombardi et al., 1986). Intramolecular resonance assists in improving Raman scattering due to coherence of analyte’s molecular vibrations with the frequency of the selected excitation wavelength. However, intramolecular resonance is affected by the area of the Raman cross-section, as larger cross- sections assist in the greater scattering of some weak scattering molecules (Tauber & Mathies, 2002). Other key parameters include the chemical structure of the analyte (Ryder, 2005) and the shape, size, and dielectric properties of the plasmonic nanostructure (Jensen et al., 2000) (Notingher & Elfick, 2005). Another cause of chemical enhancement is ground-state charge transfer, a process of electron transfer between the metal surface and the adsorbed molecules to generate charged species, which typically assist the formation of coordination compounds (Flamigni et al., n.d.) and redox reactions (Fukuzumi, 1997). However, the ground state charge transfer is associated with specific limitations, such as the selective enhancement of certain molecular vibrations of the analyte molecule, (Rurack et al., 2000) thereby limiting the uniform enhancement. Additionally, the charge transfer dynamics are affected by environmental factors (Fleming et al., 1988), the spectral overlap with other vibrational modes of the analyte (Blandamer & Fox, 1970) can limit the selectivity, and ground state saturation with the electrons of the analyte can limit any further enhancement. In the other chemical enhancement mode, resonant charge transfer was observed to overcome the limitations of ground-state charge transfer. This is the electron transfer from the resonating energy levels between the plasmonic metal and analyte molecule by charge transfer between the highest occupied molecular orbital (HOMO) and lowest unoccupied molecular orbital (LUMO), which alters the local electromagnetic field of the analyte molecule, resulting in greater Raman scattering.

 The improvement in resonant excitation enhances the plasmonic metal nanostructure and analyte interaction (McNay et al., 2011) and facilitates enhanced Localized Surface Plasmon Resonance (LSPR) of the plasmonic substrates (Kleinman et al., 2013), thereby maximizing the enhancement effect. Despite these modes of signal improvement associated with chemical enhancement, their significance to the overall average enhancement factor was marginal. Hence, the use of noble metal substrates is also being explored to achieve better signal enhancement by altering the surface chemistry of substrates and modifiers

 However, the contribution of the CE to the average SERS enhancement was not significant. Therefore, the choice of materials used for SERS substrate synthesis requires meticulous consideration of the nanostructure morphology, light absorbance range, stability, (Yamada et al., 1987) and hydrophobicity, which may be tailored to improve plasmonic resonance and electromagnetic enhancement.

 Electromagnetic enhancement

 Electromagnetic (EM) enhancement refers to the amplification of Raman-scattered photons in the proximity of a resonating electron cloud at the metal-dielectric interface. Surface plasmon resonance (SPR) is generated by the cumulative resonance on the metal surface by the coherence of the electron cloud oscillation frequency at the metal- dielectric interface with the frequency of the excitation laser. In contrast, resonance localized at a position with plasmonic nanoparticles is termed LSPR. The LSPR effect can absorb or scatter the incident laser and potentially enhance the local electromagnetic field. Local field enhancement requires the molecule to be in proximity (within ~ 100 nm) to the metal surface, that is physisorption or chemisorption. The field enhancement factor is also prominently dependent on the laser power and Stokes and anti-Stokes effects (Ding et al., 2016). Similarly, EM enhancement is a coupling effect of the local field and the re-radiated (Raman) field of the SERS substrate, as shown in Figure 3. As previously discussed, the localized electromagnetic field for metals is higher if the excitation wavelength (λ _L_) is close to the electromagnetic resonance of the system. Hence, the localized electric field (E _loc_) is dependent on light polarization and its excitation wavelength. SERS hotspots are generated if the magnitude of |E _loc_| is greater than |E _inc_|. The local field intensity enhancement factor (M _loc_ (w _L_)) would be increased by a factor of:

*M* *loc* *ω* *L* *=* *E* *loc* *ω* *L* *2* *E* *loc* *2* 

 where, (ω _L_) is the frequency of the local electric field, M _Loc_ (ω _L_) is the local field enhancement, |E _loc_ (ω _L_)| ^2^ is the magnitude of the local electric field amplitude, and |E _loc_| ^2^ is the magnitude of the incident electric field amplitude.

 In addition to local field enhancement, re-radiated field enhancement is also prominent for EM enhancement. In the electromagnetic model, the molecule is considered a dipole that responds to a greater local field near the surface (Kerker, 1984). The interaction of a metal nanostructure with light generates an LSPR effect on the nanostructure, which amplifies the incident EM field and scattered Raman field. Under SERS conditions, the radiation of a molecule’s Raman dipole in proximity to the metal surface alters the excitation field, termed modified spontaneous emission (MSE). The enhancement of the EM field is a combination of the excitation field and the Raman scattered field, which is proportional to the fourth power of the field enhancement given by equation (7), where M _Loc_ (ω _L_) is the local field enhancement, M _Loc_ (ω _R_) is the re-radiated field enhancement, |E _loc_ (ω _L_)| ^4^ is the magnitude of the local electric field amplitude, and |E _loc_| ^4^ is the magnitude of the incident electric field amplitude.

*M* *Loc* *ω* *L* *.* *M* *Loc* *ω* *R* *=* *E* *Loc* *ω* *L* *4* *E* *Inc* *4* 

 The effect of enhancement mechanism on the implications for substrate design are summarized here and also discussed in third paragraph of section 2.2 (Electromagnetic enhancement).

 The maximum EM enhancement for isolated silver or gold nanostructures/nanoparticles was observed in the range of 10 ^9^- 10 ^10^ (Le Ru et al., 2007). This was further improved to approximately 10 ^11^ by roughening the substrate surface (Camden et al., 2008). However, some significant limitations associated with electromagnetic enhancement include the near-field effect of isolated plasmonic nanomaterials that require close presence of the analyte on the substrate (Chou et al., 2012), heterogeneous signal enhancement (Schlücker, 2014) on the substrate surface that can alter the reproducibility, and amplification of background signals that obscure the weak Raman signals (Larmour et al., 2012), thereby reducing the signal-to-noise ratio (SNR) of the SERS system. A low SNR was associated with low sensitivity, poor selectivity, fluorescence, higher Rayleigh signal scattering, and low reproducibility and reliability. Thus, some considerations for transcending the low SNR of a SERS system include the synthesis of plasmonic nanoparticles with uniform shape, size, and morphology to ensure sharp absorption peaks for signal enhancement (Berciaud et al., 2005), use of pulsed lasers with short duration that minimize photobleaching of the sample (H. F. Zhang et al., 2007), use of longer-wavelength lasers (Liu et al., 2020) and spectral filters (T. Murphy et al., 2000) to reduce fluorescence of biological samples, and minimizing photobleaching effects by optimizing the laser exposure time and laser power.

**Q 8. While the article mentions various substrate designs, it could delve deeper into the specific materials and nanostructures used, as well as their advantages and limitations.**

 Response:

 The paper encompasses a discussion on the use of specific disposable grade materials such as cellulosic paper, nitrocellulosic paper, and bacterial cellulosic paper, cotton fabric, zari fabric, polyacrylonitrile fabric, PVP polymer, PET polymer, PMMA polymer, and glass-based substrates, mentioned in section VI.

 Paper-based substrates are gaining attention owing to their customizable, biodegradable, and biocompatible properties, and their scalable use in the development of consumer-oriented products. Inherent properties of cellulosic paper, such as porosity, hydrophilicity, and mechanical strength, were largely explored to fabricate SERS-based substrates. They are affordable, highly useful in resource-limited settings, and user-friendly alternatives with considerable sensitivity and specificity. Additionally, the ease of loading liquid samples will further improve sensitivity by restricting the sample flow to a small sensing region, which will aid in lateral flow assay (LFA)-based sensing techniques. However, all cellulose-based paper substrates are associated with a major limitation of autofluorescence because of the presence of organic materials. Autofluorescence may be mitigated by the use of higher-wavelength lasers and optics that block fluorescence or baseline autofluorescence. Other limitations include moisture sensitivity, flammability, and lower chemical and temperature resistance, which can be surpassed by other disposable substrates such as polymer-based and silica-based substrates.

 Fabrics serve as excellent substrates for SERS-based detection owing to their uniform interwoven fibers, durability, and resistance to wear and tear. They possess excellent porosity, microfluidic behavior, and enhanced surface area; thus, they make ideal materials for efficient contact with samples. In addition, the 300–500 μm coarse fibrous structure in plant fibers can be modified by coating or trapping plasmonic metal nanoparticles for the development of a SERS-active substrate. However, porosity may be a limitation as it may lead to the requirement of large sample volumes, which can be overcome by optimizing the sample microfluidic flow, which is regulated by the capillary pressure and wicking force of the material. However, the use of fabric-based substrates is limited due to limited durability, rapid absorption of nanomaterial or analyte into the fabric bulk, wettability control, non-uniformity of hotspots, background interference, susceptibility to environmental conditions.

 Polymer-based substrates possess characteristic features, such as durability, flexibility, and versatility, enabling their broad-spectrum applications, including medical diagnostics and environmental monitoring. Recently, the increased use of polymer-based substrates has been observed due to their chemical inertness, disposability, cost-effectiveness, and optical transparency, which facilitate the fabrication of disposable SERS substrates. However, some major considerations in the fabrication of polymer-based SERS substrates are the flexibility and optical transparency of the polymers. Silica-based substrates are beneficial due to high-temperature resistance, chemical inertness, rigidity, transparency, electrical insulation, and biocompatibility. However, glass-based substrates are not extensively used for the fabrication of disposable substrates owing to their characteristic features such as fragile nature, optical transparency, high photoluminescence. In conclusion, the use of all disposable substrates for SERS-based sensing at the small-scale or industrial level was beneficial because of its cost-effectiveness, ease of customizability, portability, and scalability, despite some challenges in substrate performance. Therefore, disposable SERS substrates are promising for sensing target analytes in food safety, biomedical diagnostics, and environmental monitoring, to address real-world challenges.

 A review of specific nanostructures used in the substrate design was discussed in section X.

 0D materials such as silver nanoparticles and gold nanoparticles grown in-situ (Verma et al., 2021) or decorated (Geng et al., 2020),  or printed on the substrate material (W. Wu et al., 2015) were used in the development of plasmonic substrates. These 0D materials display strong LSPR with excellent colloidal stability and size-tunable optical properties. However, their extensive use is limited due to uncontrolled aggregation, limited hotspot density and thus lower EF.

 1D materials such as silver nanorods embedded into PDMS film (Kumar et al., 2017) gold nanorods on polystyrene microspheres (H. Yang et al., 2017), gold@silver nanorods decorated on glass (L. Wu et al., 2019a) were used in the development of plasmonic substrates. These materials offer advantages such as anisotropic optical properties, directional plasmonic enhancement, and good surface area to volume ratio. However, the synthesis of these materials is elaborate and tedious with low repeatability, in addition to complex alignment startegies on the surface materials.

 2D materials such as silver nanodisks decorated paper (Pagano et al., 2021) were used in the development of plasmonic substrates. These materials display greater hotspot density at edges and tip curvatures, high signal uniformity and great hybridization capability due to larger active surface area. However, these materials are difficult to fabricate owing to their precise deposition and structuring, in addition to the inhomogeneous distribution of hotpots affecting their signal enhancement.

 3D materials such as gold nanostars embedded into PDMS film (X. Ma et al., 2022), 3D silver nanowire mesh on glass (X. Li et al., 2014), gold nanostars embedded into PMMA film (Riswana Barveen et al., 2022) were used in the development of plasmonic substrates. These materials show great potential for trace analyte detection, due to strong plasmonic coupling and highest hotspots density resulting in exceptional signal amplification. However, the scalability of these materials for commercial use is limited due to poor stability, low reproducibility for consistent structures, difficult surface functionalization, and limited control of hotspot distribution.

**Q 9. The section 5.1 needs to restructure and should be supported by proper references.**

 Response:

 The section 5.1 (Hotspots) was restructured for better readability and supported with proper references from page 30-32 in the revised manuscript, as follows:

 5.1 Hotspots

 SERS hotspots are localized nanozones with intense plasmonic fields that enhance the resonance and thus the Raman scattering. The interaction of incident light with plasmonic nanostructures results in a highly concentrated and localized EM field, and an exceptional enhancement in Raman scattering, termed as hotspot (Freeman et al., 1995). SERS hotspots assist in the amplification of Raman scattering by multiplicative enhancement of the plasmonic field of nanostructures , resulting in synergistic effects with spatial localization facilitating selective enhancement of signals (Qin et al., 2006) from the hotspot and reduces the background interference (Le Ru & Etchegoin, 2004). Dynamics of the structure and orientation (Mulvaney & Keating, 2000) of the plasmonic nanostructure and their aggregation affects the dimensionality of the nanosubstrate (Futamata et al., 2003) and thus the signal enhancement. The SERS enhancement factor is calculated as the ratio of the SERS intensity (I _SERS_) to the Raman intensity (I _Raman_) (Vo-Dinh, 1998b). The Raman scattering intensity is mathematically expressed as the square of the electric field energy of the incident light (|E| ^2^) and expressed as (|E| ^4^) in the proximity of a plasmonic nanoparticle (Tian et al., 2002). The generation of hotspots is crucial for the design of SERS materials and platforms to improve nanoscale light confinement (Hao & Schatz, 2004). Hence, extensive research is being pursued on the synthesis and incorporation of nanomaterials ranging from 0D to 3D for engineering hotspots. Table IV provides an overview of the common nanostructures used in hotspot generation, and some unique properties that assist in higher signal enhancement.

 Hotspot engineering is crucial for the development of SERS-active substrates with nanoscale light confinement in specific localized regions of 0D to 3D materials. In the case of 0D materials, such as quantum dots, a plasmonic field structure with confined dimensionality is not possible (H. P. Lu, 2005). However, quantum confinement of the discrete energy levels can generate localized field that can be engineered to generate hotspots (Johnson, 1995), and the near-field effect can tailor the plasmonic field magnitude and structure  (Pereyra & Ulloa, 2000) . Common domains of applications include the detection of small molecules (Weisbuch et al., 2000) and biomolecules (Chan & Nie, 1998). In the case of 1D materials, such as nanorods and nanowires, a greater plasmonic field is observed at the tip curvature, which contributes to longitudinal plasmon absorbance (Clapp et al., 2004). Regularly ordered alignment (Nikoobakht et al., 2002), surface roughness (Fan et al., 2004), and tip engineering (Fan & Zhao, 2005) of 1D materials can be used to tailor plasmonic field structures. These materials are mostly employed for the detection of small molecules (Kneipp et al., 1998), inorganic molecules (Christof M. Niemeyer, 2001), and heavy metal ions (Muniz-Miranda & Sbrana, 2001) in biological or non-biological samples. For 2D materials such as nanoparticle films, graphene, and graphene oxide, the plasmonic field structure is determined by the nanogaps or crevices of adjacent nanosheets or nanoparticles (Grigorenko et al., 2012). Cumulative surface plasmon polaritons (SPP) can enhance the local field by coupling with 1D and 3D materials to tailor the plasmonic-field structure (Zayats et al., 2005). 2D materials are commonly used for the label- free detection of biomolecules. In the case of 3D materials such as microfabricated nanoholes, nanopillars, and nanopyramids, the plasmonic field structure is generated by bulk plasmon resonance (Wei, 2004). Optimization of the 3D material geometry and customizing the dielectric environment can tailor the plasmonic field structure, and thus assist in hotspot engineering. Three-dimensional (3D) materials are commonly used in the detection of biological and non-biological organic molecules, food sample analysis, and forensic analysis.

**Q 10. The table I, II and IV should be supported by proper references. Change the heading of figure IV considering plasmonic nanostructures are not hotspot.**

 Response:

 Table I, II, IV briefly summarizes the list of commonly used laser diodes, their emission wavelengths, and the commonly detected target analytes, the list of commonly used photodiodes, their detection range, characteristics and commonly detected target analytes, the list of common plasmonic nanostructures used as SERS substrates and their unique features for signal enhancement respectively, and supported with proper references.

 The heading of section IV has been rephrased as “List of common plasmonic nanostructures used as SERS substrates and their unique features for signal enhancement” in page 30, section 5.1.

**Q 11. In “One of the widely accepted theories for CE is the charge transfer (CT) mechanism proposed by the SERS pioneer Andreas Otto, where the molecule is chemically adsorbed on the substrate, which subsequently changes the molecular polarizability, thereby enhancing the Raman spectrum. Check if it is Raman spectrum or Raman scattering.**

 Response:

 The statement has been rephrased as “One of the widely accepted theories for CE is the charge transfer (CT) mechanism proposed by the SERS pioneer Andreas Otto, where the molecule is chemically adsorbed on the substrate, which subsequently changes the molecular polarizability, thereby enhancing the Raman scattering.” in second paragraph of section 2.1 (Chemical Enhancement)

 Q 12. In the discussion of paper substrate, the authors can also include following papers

https://doi.org/10.1016/j.saa.2023.122610,

**DOI** 10.1088/1361-6463/aa8fef

 Response:

 The mentioned research papers were identified to be relevant and cited in table V (List of paper-based substrates for SERS-based sensors).

1.
**Spectrochimica Acta Part A: Molecular and Biomolecular Spectroscopy,** **295**

**(2023), 122610**: This study explores the use of silver nanoparticles functionalized paper for the detection of rotavirus in clinical stool samples. The performance of the developed SERS-active substrate was equivalent with standard rotavirus detection methods. The high sensitivity and robustness of this paper-based sensor make them an appropriate choice for use in biomedical diagnostics.

1.
**Journal of Physics D: Applied Physics,** **50 (2017), 485601**: This study demonstrates
  Polyvinylpyrrolidone (PVP) capped silver nanoparticles imprinted paper for detection of 1, 2-bis(4-pyridyl) ethylene in artificial urine samples. This low-cost paper-based sensor showed high sensitivity requiring minimal volume of plasmonic nanostructures, showing their potential for use in on-site applications.

 

**Q 13. Consider rephrasing of following sentences for better readability-**

1. **Table I 5 ^th^ row-** They don't fluoresce, but poor Raman scattering efficiency as compared to visible range lasers.
2. **Section 3.3 last paragraph- **The cooling of the photodetector in a Raman system reduces the dark noise, which can be attributed to the dark current. These dark currents influence the photodetector temperature, which requires intermediate cooling. Cooling the photodetector also aids in reducing background noise and improving the SNR of the sensing system.
3. **Section 10 last paragraph-** Thus, the measured Raman spectra should be free of trace impurities to avoid the background signal and provide a Raman signal.

Response:

 The mentioned sentences were rephrased for better readability, as follows:

1. The statement “They don't fluoresce, but poor Raman scattering efficiency as compared to visible range lasers,” has been rephrased as “These lasers do not fluoresce, but generate poor Raman scattering than visible range lasers” in page 14, table I 5 ^th^ row.
2. The statement “The cooling of the photodetector in a Raman system reduces the dark noise, which can be attributed to the dark current. These dark currents influence the photodetector temperature, which requires intermediate cooling. Cooling the photodetector also aids in reducing background noise and improving the SNR of the sensing system” has been rephrased as “The cooling of the photodetector in a Raman system reduces the dark noise, which originates from the dark current. As, these dark currents are influenced by the photodetector temperature, which requires intermediate cooling, it can aid in reducing background noise and improving the SNR of the sensing system” in page 19, last paragraph, section 3.3.
3. The statement “Thus, the measured Raman spectra should be free of trace impurities to avoid the background signal and provide a Raman signal” has been rephrased as “Therefore, to ensure a sensitive and analyte-specific Raman signal with minimum background noise, the measured Raman spectra should be free of trace impurities” in section 9 last paragraph.

**Q 14. The article should conclude with a discussion on future research directions and emerging trends in SERS. Identifying areas where further research is needed would provide valuable insights for researchers in the field.**

 Response:

  The emerging trends in SERS are briefly summarized here:

 Portable SERS-based systems are crucial for on-site detection of diverse range of analytes, with these systems offering rapid, efficient, sensitive, and selective sensing capabilities, making them valuable in food quality control, environmental monitoring, and biomedical diagnostics. Despite advancements in SERS technology, commercial-scale applications require user-friendly and robust sensors. Recent studies have developed custom portable SERS systems for detecting Sudan I in paprika extracts and melamine in milk samples. These systems combine techniques like thin-layer chromatography and molecularly imprinted polymers with SERS, utilizing portable spectrometers equipped with laser diodes and fiber optic probes. The portability and user-friendliness of these systems enhance their practicality for real-world applications, though they still require trained personnel for optimal use. A comprehensive review of recent advancements in portable-SERS sensing systems was included in page 99, section 12 of the modified manuscript.

 In addition to the development of portable sensing systems, a promising avenue for exploration is the integration with intelligence enabled technologies **. **The integration of AI with SERS-based sensing systems enhances detection accuracy employing machine learning (ML) algorithms for data analysis and pattern identification. Supervised learning algorithms like support vector machines (SVM), random forests (RF), and neural networks enable real-time analyte detection from complex specimens. Techniques such as convolutional neural networks (CNNs) and recurrent neural networks (RNNs) classify hierarchical patterns and temporal data, crucial for real-time analysis. Clustering algorithms like K-means and Gaussian mixture models facilitate spectral pattern identification and anomaly detection. For quantitative analysis, artificial neural networks (ANN) and partial least squares regression (PLSR) predict target analyte concentrations. Principal component analysis (PCA) and autoencoders are used for feature extraction and dimensionality reduction. Additionally, genetic algorithms optimize experimental parameters, while probabilistic modeling with Bayesian inference enhances system robustness and reproducibility. This integration significantly improves the efficiency and reliability of SERS-based sensing systems. A comprehensive review of recent advancements in portable-SERS sensing systems was included in page 99-100, section 13 of the modified manuscript.

 The future research directions in SERS were discussed in the conclusion, as follows: 

 SERS based detection also shows a great promise in integration with point-of-care technologies for the development of wearable devices and POU devices for deployment in resource-limited settings. The use of statistical methods, such as correlation analysis and multivariate approaches, and machine learning algorithms, such as supervised, unsupervised and hybrid learning algorithms, facilitate the analysis of complex SERS data sets to decipher intricate signals from biological samples. The integration of microfluidic channels and sample preconcentration may further improve the analytical performance. In summary, disposable material-based plasmonic substrates used for POU SERS- based sensing represent a viable method for quick, accurate, and useful analyses in a variety of biological, environmental, and security applications. Further investigation and advancement will augment the comprehensibility and expand the applicability of this technology across several domains.
